# Rethinking Pediatric Asthma Education Through Large Language Model Generation and Simplification: Randomized Double-Blind Study

**DOI:** 10.2196/98118

**Published:** 2026-08-26

**Authors:** Tianyi Xu, Yong Yin, Xi Zhang, Wei Wei, Jiajun Yuan, Hansong Wang, Guodong Ding, Wenjie Xue, Ziwei Chen, Sixin Xie, Huiqin Niu, Jie Xi, Shuzhu Lin, Xiaoli Tang, Liebin Zhao

**Affiliations:** 1 Songjiang Research Institute, Songjiang District Central Hospital, No. 746, Zhongshan Middle Road, Songjiang District, Shanghai, 201600, China, 86 18930830660; 2 School of Public Health, School of Medicine, Shanghai Jiao Tong University, Shanghai, China; 3 Shanghai Children's Medical Center, Shanghai, China; 4 Shanghai Engineering Research Center of Intelligence Pediatrics, Shanghai, China; 5 XinHua Hospital, Shanghai, China; 6 Yuyuan Subdistrict Community Health Center, Huangpu District, Shanghai, China; 7 Shanghai Second People's Hospital, Shanghai, China; 8 Bansongyuan Subdistrict Community Health Center, Huangpu District, Shanghai, China; 9 Nanjing East Road Subdistrict Community Health Center, Shanghai, China; 10 Laoximen Subdistrict Community Health Center, Shanghai, China; 11 Wuliqiao Subdistrict Community Health Center, Huangpu District, Shanghai, China; 12 Fengcheng Hospital, Shanghai, China; 13 School of Health Management, Southern Medical University, Guangzhou, China

**Keywords:** large language model, LLM, health education materials, information adoption model, IAM, ethics

## Abstract

**Background:**

Large language models (LLMs) are increasingly used to generate health education materials, yet questions remain about whether LLM-generated content can balance professional accuracy with public accessibility and what ethical challenges may arise during deployment.

**Objective:**

This study aimed to evaluate Chinese pediatric asthma educational materials generated using LLMs, comparing AI-generated responses (AI-GRs) against published expert-authored responses (PEARs) on total scores, adoption intent among health care professionals, perceived usefulness among family members, and the effect of AI-simplified responses (AI-SRs), as well as to assess participants’ ability to correctly identify the source of materials.

**Methods:**

In this randomized, double-blind evaluation study, participants (medical professionals and patient family members) were randomly assigned (1:1:1) to evaluate PEARs, AI-GRs, or AI-SRs. Each participant assessed 5 randomly selected items from their assigned set using a 19-item, 5-point Likert scale based on the information adoption model. Secondary outcomes included dimension-specific scores, source identification accuracy, and readability indices.

**Results:**

AI-GRs had numerically higher mean total scores on the 19-item questionnaire than PEARs among both medical professionals (76.17, SD 12.59 vs 72.97, SD 14.88; Holm-adjusted *P*=.53) and pediatric patient families (68.77, SD 16.47 vs 64.18, SD 15.76; Holm-adjusted *P*=.15). The corresponding mean scores for AI-simplified responses were 72.98 (SD 12.64) and 65.47 (SD 16.52), respectively, with no statistically significant differences from PEARs after Holm adjustment (both adjusted *P*>.99). No statistically significant differences were detected between either LLM group and PEARs in any of the 4 questionnaire dimensions after Holm adjustment. The material group–population interaction was also not statistically significant (*F*_2,513_=0.116; *P*=.89; partial η^2^<0.001). Regarding source identification, 80.6% (141/175) of participants assigned to PEARs identified the material as human-authored, whereas only 7.3% (25/344) of those assigned to the LLM groups identified the material as LLM-generated.

**Conclusions:**

This randomized, double-blind evaluation study provides preliminary participant-level evidence regarding the perceived quality and acceptance of LLM-assisted pediatric asthma education among medical professionals and pediatric patient families under controlled conditions. Additional language simplification did not improve acceptance in this predominantly highly educated sample, and the strong tendency to attribute materials to human authors highlights the importance of source transparency. Professional review and dedicated assessments of factual accuracy, clinical safety, comprehension, and behavioral outcomes remain necessary before practical implementation.

## Introduction

Traditional health education materials face systemic challenges, including limited resources, poor accessibility of specialized content, and health equity gaps. Evidence confirms that professional medical content does not equate to effective health education materials, which must balance scientific rigor with public comprehensibility and acceptability [1]. However, more than 40% of the public struggles with medical terminology [2], while excessive simplification often omits clinically important context. Across health care, AI capabilities span diagnosis, prediction, clinical decision support, workflow assistance, monitoring, and communication [3]. Large language models (LLMs), with their advanced reasoning and multitask capabilities, offer a technological foundation for precise and personalized medical communication [4,5]. Their effectiveness in improving care, streamlining communication, and reducing costs has been documented [6-9].

Asthma, the most common chronic respiratory disease in children, requires effective management that heavily depends on caregivers’ knowledge of symptoms, treatment, medication, device use, and environmental triggers. While some studies have compared LLM-generated responses to asthma questions, the direct application of LLMs for creating patient education materials remains underexplored in real-world settings [10-12]. Existing evaluations primarily focus on content-level metrics such as accuracy and comprehensiveness among medical professionals [13,14], with limited extension to how both professionals and intended lay users perceive the quality, credibility, usefulness, and adoption of such materials.

Therefore, this study used the 19-item scale based on the information adoption model to evaluate pediatric asthma materials comprising published expert-authored responses (PEARs), GPT-4o–generated responses, and GPT-4o–simplified responses from the dual perspectives of medical professionals and pediatric patient families. We assessed key dimensions including quality, credibility, usefulness, and adoption intention, aiming to (1) compare participant ratings of the 3 material types across these dimensions and examine whether additional linguistic simplification changed the ratings and (2) assess source identification and practical considerations relevant to the responsible use of LLM-generated health education materials.

## Methods

### Study Design

**Figure 1.**
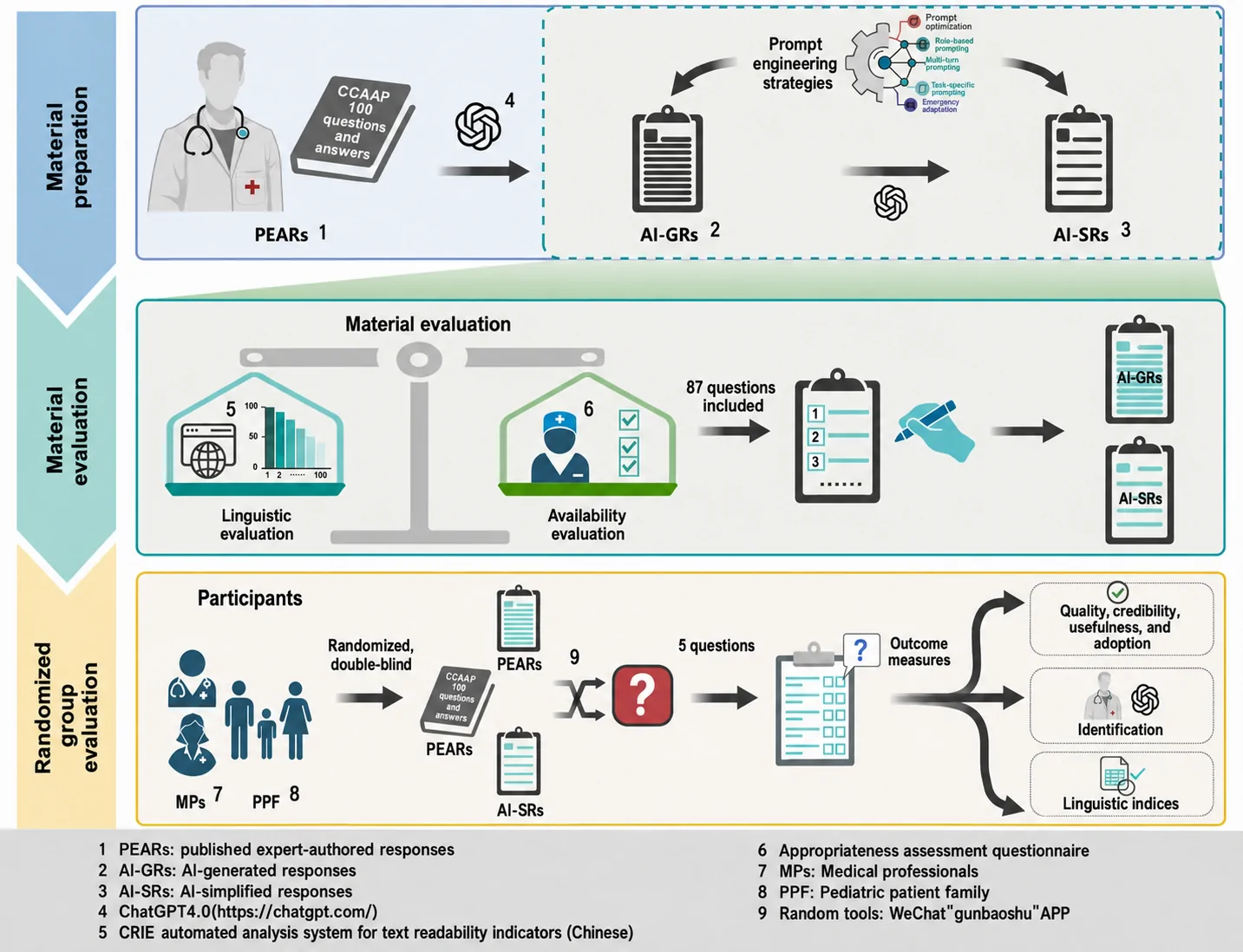
Schematic diagram of the study design. (1) PEARs: published expert-authored responses; (2) AI-GRs: AI-generated responses; (3) AI-SRs: AI-simplified responses; (4) GPT-4o [15]; (5) CRIE: Chinese Readability Index Explorer (automated analysis system for text readability indicators); (6) appropriateness assessment questionnaire; (7) MPs: medical professionals; (8) PPF: pediatric patient family members; and (9) WeChat “qunbaoshu” app, randomization tool.

This multicenter, randomized, parallel-group, double-blind evaluation was conducted in Shanghai. Medical professionals and pediatric patient family members were enrolled and randomly assigned to 1 of 3 groups to evaluate the quality of health education materials from different sources via questionnaire (Figure 1). Participants did not receive any clinical, therapeutic, preventive, or behavioral intervention intended to modify a health-related outcome, and no biomedical, clinical, or patient health outcomes were assessed. The study was registered with the China Clinical Trial Registry under an observational study classification and was retrospectively registered on November 9, 2025 (ChiCTR2500112000). The study design, randomized allocation scheme, evaluation instrument, and primary outcome were determined before participant enrollment and before examination of the study results. No outcome was added, removed, or reclassified on the basis of the observed results.

### Ethical Considerations

This study was approved by the Ethics Committee of Shanghai Children’s Medical Center, Shanghai Jiao Tong University School of Medicine (SCMCIRB-K2024201-1) before participant recruitment began. Written informed consent was obtained from all participants.

### Study Population

Participants were recruited between December 2024 and August 2025. A purposive sampling strategy was used to ensure representation across diverse health care settings. Health care professionals were recruited from 2 tertiary hospitals, 1 secondary hospital, and 5 community health service centers. Family members of patients were recruited from 1 tertiary hospital and 1 secondary hospital.

Eligibility criteria for medical professionals included: (1) meeting professional requirements, (2) willingness to participate, and (3) ability to independently complete the questionnaire. Eligibility criteria for pediatric patient family members included (1) being the primary caregiver of a child with asthma, (2) voluntary participation, (3) ability to complete the questionnaire independently, and (4) no self-reported history of conflicts with health care providers.

Data collection was conducted by 9 trained and briefed medical students from Shanghai Jiao Tong University School of Medicine.

### Blinding and Quality Control

Both investigators and participants remained blinded to the source of the materials throughout the study. To ensure data quality, 2 attention-check questions were embedded in the electronic questionnaire. A final question assessed whether participants perceived the materials as LLM-generated. All materials were standardized in font and format to prevent identification based on textual characteristics.

### Material Generation and Randomization

**Figure 2.**
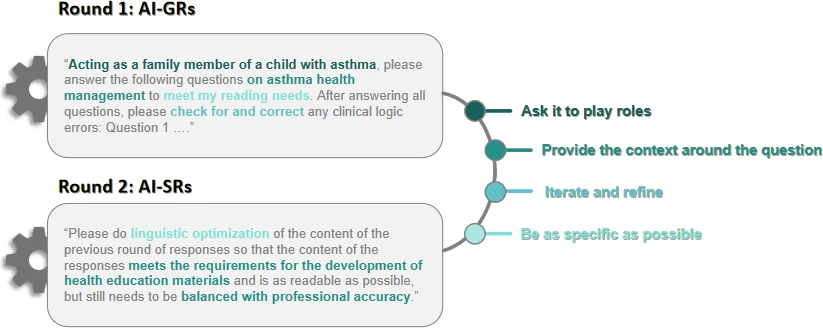
Case study of generating AI-generated responses (AI-GRs) and AI-simplified responses (AI-SRs) using prompt engineering.

All source questions and educational responses used in this study were presented in simplified Chinese. The PEARs were sourced from the validated Chinese Children’s Asthma Action Plan: 100 Questions and Answers [16]. All AI-generated materials were produced using GPT-4o (OpenAI) [17] through the ChatGPT web interface between August 13 and August 29, 2024. Materials were generated using the default interface settings without additional parameter adjustments by the researchers. For each question, the prompt was submitted once to generate one AI-generated response (AI-GR). Using prompt engineering strategies based on established methodologies [18], simplification instructions were applied to create AI-simplified responses (AI-SRs), aiming to enhance readability while preserving core meaning (Figure 2). Subsequently, each simplification prompt was submitted once to generate the corresponding AI-SR. No multiple candidate responses were generated, compared, or selectively retained. The complete prompts and detailed generation workflow are provided in Multimedia Appendix 1.

A pediatric pulmonologist with >10 years of experience screened all generated content using a 5-point Likert scale (Multimedia Appendix 2). In total, 87 items (excluding 13 problematic ones) scoring ≥2 points were retained for the final item banks. Additionally, GPT-4o generated AI-GRs with a total response time of 357 seconds and AI-SRs with a total response time of 341 seconds.

Participants were randomly assigned in a 1:1:1 ratio to 1 of 3 parallel material groups: PEARs, AI-GRs, and AI-SRs. The 1:1:1 ratio referred to participant-level assignment across the 3 groups; each participant evaluated only the type of material corresponding to the assigned group. Within each parallel group, each participant was presented with 5 question-response pairs randomly selected from the corresponding material pool (via a random number generator). After reviewing the 5 pairs, each participant completed the 19-item questionnaire based on the information adoption model once to provide an overall evaluation of the material set and contributed one total scale score. Randomization was used solely to determine the material bank evaluated by each participant; no clinical or health-related intervention intended to modify a health outcome was administered.

### Outcome Measures

The primary outcome was the total score of a 19-item, 5-point Likert scale (1=strongly disagree to 5=strongly agree) based on the information adoption model, assessing 4 dimensions: information quality [19,20], credibility [21-23], usefulness [24,25], and adoption intention [26]. The scale demonstrated good internal consistency (Cronbach α >0.8 for total and subscale scores) and satisfactory construct validity (all factor loadings >0.5).

Secondary outcomes included individual dimension scores (quality: 4 items; credibility: 6 items; usefulness: 6 items; adoption intention: 3 items). Linguistic features (total words, sentences, difficult words, and mean sentence length) were analyzed using the Chinese Readability Index Explorer [27-30]. Each participant evaluated the source of the selected assessment materials at the end of the questionnaire (whether authored by medical professionals or generated by other tools). Demographic data (age, gender, education, work experience, residence, income, etc) were collected.

### Statistical Analysis

Sample size was calculated using G*Power (version 3.1.9.2) based on a 3-group comparison of total scores. With an effect size Cohen *d*=0.5, α=.025 (2-sided), and power (1–β)=0.80, 192 participants were required. Accounting for 20% attrition, 240 participants (80 per group) were targeted.

Scale scores were analyzed using general linear models including material source (PEARs, AI-GRs, and AI-SRs), participant population (medical professionals and pediatric patient families), and their interaction. *F* statistics, df, *P* values, and partial η^2^ were reported for each model effect. On the basis of estimated marginal means, prespecified comparisons of AI-GRs versus PEARs and AI-SRs versus PEARs were conducted within each participant population. The 4 prespecified comparisons for the total scale score were adjusted collectively using the Holm procedure. Model-estimated mean differences, 95% CIs, unadjusted *P* values, and Holm-adjusted *P* values were reported. Statistical significance was defined as a 2-sided Holm-adjusted *P* value of <.05. The same models were used for secondary analyses of the 4 questionnaire dimensions. The 16 comparisons arising from the 2 participant populations, 4 dimensions, and 2 prespecified comparisons per dimension were treated as a separate family and adjusted using the Holm procedure. Results were interpreted according to the adjusted *P* values. Subgroup analyses were restricted to the total score and were considered exploratory. Demographic data were summarized using medians (IQRs) for continuous variables and percentages for categorical variables. Linguistic measures were summarized as medians and IQRs and compared across the 3 matched versions using Friedman tests, followed by paired Wilcoxon signed-rank tests. The 12 pairwise comparisons across the 4 linguistic measures were collectively adjusted using the Holm method.

## Results

### Participant Characteristics

Among 267 approached medical professionals and 330 patients’ family members, 243 (91%) professionals (PEARs: n=79, 32.5%; AI-GRs: n=81, 33.3%; AI-SRs: n=83, 34.2%) and 276 (83.6%) patients’ family members (PEARs: n=96, 34.8%; AI-GRs: n=90, 32.6%; AI-SRs: n=90, 32.6%) completed the evaluation and passed attention checks.

The median age of medical professionals was 37 (range 30‐46) years. Most were women (194/243, 79.8%), and highly educated (231/243, 95% held bachelor’s degrees or higher). More than half (129/243, 53.1%) had 10 or more years of experience, with 40.7% (99/243) pediatricians, 36.2% (88/243) general practitioners, and 23% (56/243) pediatric nurses. Patients’ family members were primarily parents (265/276, 96%), with a median age of 38 (range 35‐41) years, and 71.4% (197/276) were women. Most (235/276, 85.6%) of them had a bachelor’s degree or higher, 77.2% (213/276) resided in urban areas, and 58.3% (161/276) reported monthly household incomes exceeding ¥15,000 (¥1=US $0.15 as of August 17, 2026). The children under their care were predominantly boys (177/276, 64.1%), with a mean age of 8.1 years; 81.2% (224/276) were receiving medication, and 59.8% (165/276) had asthma for more than 1 year. Nearly all participants (233/243, 95.9% professionals; 262/276, 94.9% caregivers) had prior experience using electronic devices (Table 1).

**Table 1.** Characteristics of participants.

Variables	Overall	PEARs^a^	AI-GRs^b^	AI-SRs^c^
Medical professionals^d^				
Gender, n (%)				
Women	194 (79.8)	59 (74.7)	64 (79)	71 (85.5)
Men	49 (20.2)	20 (25.3)	17 (21)	12 (14.5)
Age (y), n (%)				
<37	114 (46.9)	38 (48.1)	39 (48.1)	37 (44.6)
≥37	129 (53.1)	41 (51.9)	42 (51.9)	46 (55.4)
Education, n (%)				
High school and below	12 (4.9)	4 (5.1)	6 (7.4)	2 (2.4)
Bachelor’s degree	140 (57.6)	47 (59.5)	39 (48.2)	54 (65.1)
Master’s degree and above	91 (37.4)	28 (35.4)	36 (44.4)	27 (32.5)
Profession, n (%)				
Pediatrician	99 (40.7)	30 (38)	36 (44.4)	33 (39.8)
General practitioner	88 (36.2)	27 (34.2)	30 (37)	31 (37.3)
Pediatric nurse	56 (23)	22 (27.8)	15 (18.5)	19 (22.9)
Years of work experience, n (%)				
<10	114 (46.9)	38 (48.1)	39 (48.1)	37 (44.6)
≥10	129 (53.1)	41 (51.9)	42 (51.9)	46 (55.4)
Pediatric patient family^e^				
Gender, n (%)				
Women	197 (71.4)	70 (72.9)	60 (66.7)	67 (74.4)
Men	79 (28.6)	26 (27.1)	30 (33.3)	23 (25.6)
Age (y), n (%)				
<37	137 (49.6)	53 (55.2)	40 (44.4)	44 (48.9)
≥37	139 (50.4)	43 (44.8)	50 (55.6)	46 (51.1)
Education, n (%)				
High school and below	41 (14.9)	15 (15.6)	12 (13.3)	14 (15.6)
Bachelor’s degree	181 (65.6)	63 (65.6)	60 (66.7)	58 (64.4)
Master’s degree and above	54 (19.6)	18 (18.8)	18 (20)	18 (20)
Residence, n (%)				
Urban	213 (77.2)	78 (81.3)	64 (71.1)	71 (78.9)
Rural	63 (22.8)	18 (18.8)	26 (28.9)	19 (21.1)
Income (¥; ¥1=US $0.15 as of August 17, 2026), n (%)				
<10,000	57 (20.7)	13 (13.5)	23 (25.6)	21 (23.3)
10,000‐20,000	116 (42)	41 (42.7)	33 (36.7)	42 (46.7)
≥20,000	103 (37.3)	42 (43.8)	34 (37.8)	27 (30)

a PEAR: published expert-authored responses.

b AI-GR: AI-generated responses.

c AI-SR: AI-simplified responses.

d Overall: n=243; PEAR: n=79; AI-GR: n=81; AI-SR: n=83.

e Overall: n=276; PEAR: n=96; AI-GR: n=90; AI-SR: n=90.

### Overall Evaluation

In the general linear model for the overall scores, the main effect of material source was statistically significant (*F*_2,513_=3.330; *P*=.04; partial η^2^=0.013), and the effect size was small. A statistically significant main effect of participant population was also observed (*F*_1,513_=35.984; *P*<.001; partial η^2^=0.066), indicating a difference in overall scores between medical professionals and caregivers. The material source-by-participant population interaction was not statistically significant (*F*_2,513_=0.116; *P*=.89; partial η^2^<0.001), providing no statistical evidence that differences among the material sources varied according to participant population (Table 2).

**Table 2.** General linear model results for overall and four-dimensional scores.

Outcome and model effect	*F* test (*df*)	*P* value	Partial η^2^
Overall			
Material source	3.330 (2, 513)	.04	0.013
Participant population	35.984 (1, 513)	<.001	0.066
Material source-by-participant population	0.116 (2, 513)	.89	<0.001
Information quality			
Material source	2.144 (2, 513)	.12	0.008
Participant population	42.238 (1, 513)	<.001	0.076
Material source-by-participant population	0.081 (2, 513)	.92	<0.001
Information credibility			
Material source	1.232 (2, 513)	.29	0.005
Participant population	30.237 (1, 513)	<.001	0.056
Material source-by-participant population	0.449 (2, 513)	.64	0.002
Information usefulness			
Material source	3.499 (2, 513)	.03	0.013
Participant population	16.088 (1, 513)	<.001	0.030
Material source-by-participant population	0.390 (2, 513)	.68	0.002
Information adoption			
Material source	3.513 (2, 513)	.03	0.014
Participant population	13.962 (1, 513)	<.001	0.026
Material source-by-participant population	0.073 (2, 513)	.93	<0.001

Among medical professionals, mean total evaluation scores were 72.97 (SD 14.88) for PEARs, 76.17 (SD 12.59) for AI-GRs, and 72.98 (SD 12.64) for AI-SRs. The model-estimated mean difference was 3.20 for AI-GRs (95% CI −0.99 to 7.39; unadjusted *P*=.13; Holm-adjusted *P*=.53) and 0.01 for AI-SRs (95% CI −4.18 to 4.20; unadjusted *P*>.99; Holm-adjusted *P*>.99). Corresponding mean scores among pediatric patient family were 64.18 (SD 15.76), 68.77 (SD 16.47), and 65.47 (SD 16.52). AI-GRs had a numerically higher score than PEARs, with a model-estimated mean difference of 4.59 (95% CI 0.28-8.90) and an unadjusted *P* value of .037. However, this difference was no longer statistically significant after Holm adjustment across the 4 predefined total score comparisons (adjusted *P*=.15). The model-estimated mean difference between AI-SRs and PEARs was 1.29 (95% CI −3.03 to 5.61; unadjusted *P*=.56; Holm-adjusted *P*>.99; Table 3).

**Table 3.** Model-estimated comparisons with Holm adjustment for overall and 4-dimensional scores.

Comparison	PEARs^a^, mean (SD)	AI group, mean (SD)	Mean difference (95% CI)	*P* value	Holm-adjusted *P* value
Medical professional					
Overall					
AI-GRs^b^ vs PEARs	72.97 (14.88)	76.17 (12.59)	3.20 (−0.99 to 7.39)	.13	.53
AI-SRs^c^ vs PEARs	72.97 (14.88)	72.98 (12.64)	0.01 (−4.18 to 4.20)	>.99	>.99
Information quality					
AI-GRs vs PEARs	15.92 (3.60)	16.72 (3.06)	0.79 (−0.36 to 1.94)	.18	>.99
AI-SRs vs PEARs	15.92 (3.60)	15.99 (3.30)	0.06 (−1.08 to 1.21)	.91	>.99
Information credibility					
AI-GRs vs PEARs	23.54 (5.41)	23.77 (5.47)	0.22 (−1.58 to 2.02)	.81	>.99
AI-SRs vs PEARs	23.54 (5.41)	23.36 (4.91)	−0.18 (−1.97 to 1.60)	.84	>.99
Information usefulness					
AI-GRs vs PEARs	22.56 (5.67)	23.88 (4.96)	1.32 (−0.48 to 3.12)	.15	>.99
AI-SRs vs PEARs	22.56 (5.67)	22.53 (5.29)	−0.03 (−1.82 to 1.77)	.98	>.99
Information adoption					
AI-GRs vs PEARs	10.95 (2.64)	11.81 (2.48)	0.87 (−0.02 to 1.75)	.06	.82
AI-SRs vs PEARs	10.95 (2.64)	11.10 (2.76)	0.15 (−0.73 to 1.03)	.74	>.99
Pediatric patient family					
Overall					
AI-GRs vs PEARs	64.18 (15.76)	68.77 (16.47)	4.59 (0.28 to 8.90)	.04	.15
AI-SRs vs PEARs	64.18 (15.76)	65.47 (16.52)	1.29 (−3.03 to 5.61)	.56	>.99
Information quality					
AI-GRs vs PEARs	13.71 (3.97)	14.51 (4.02)	0.80 (−0.26 to 1.87)	.14	>.99
AI-SRs vs PEARs	13.71 (3.97)	14.06 (4.03)	0.35 (−0.72 to 1.42)	.52	>.99
Information credibility					
AI-GRs vs PEARs	20.47 (5.82)	21.64 (6.37)	1.17 (−0.49 to 2.84)	.17	>.99
AI-SRs vs PEARs	20.47 (5.82)	20.16 (6.46)	−0.31 (−1.98 to 1.36)	.71	>.99
Information usefulness					
AI-GRs vs PEARs	19.94 (6.20)	21.87 (6.24)	1.93 (0.26 to 3.60)	.02	.38
AI-SRs vs PEARs	19.94 (6.20)	21.01 (6.18)	1.07 (−0.60 to 2.75)	.21	>.99
Information adoption					
AI-GRs vs PEARs	10.06 (2.98)	10.74 (2.97)	0.68 (−0.14 to 1.50)	.10	>.99
AI-SRs vs PEARs	10.06 (2.98)	10.24 (3.14)	0.18 (−0.64 to 1.00)	.66	>.99

a PEAR: published expert-authored response.

b AI-GR: AI-generated material.

c AI-SR: AI-simplified version.

General linear model analyses showed significant main effects of participant population across all 4 dimensions, with medical professionals generally providing higher ratings than pediatric patient families (all *P*<.001). Material source had significant overall main effects on the information usefulness and information adoption dimensions (both *P*=.03), but not on the information quality or information credibility dimensions. No significant interactions between material source and participant population were observed for any dimension, indicating that the effects of material source did not differ significantly between the two participant populations (Table 2).

In the prespecified comparisons, the only nominally significant unadjusted difference was observed for information usefulness among pediatric patient families, with AI-GRs receiving higher scores than PEARs (mean difference 1.93, 95% CI 0.26-3.60; *P*=.02). However, this difference did not remain statistically significant after Holm adjustment across the 16 dimension-specific comparisons (adjusted *P*=.38). None of the remaining comparisons were statistically significant either before or after adjustment. Thus, the adjusted analyses provided no evidence of significant differences between AI-GRs or AI-SRs and PEARs on any individual scale dimension (Table 3).

**Figure 3.**
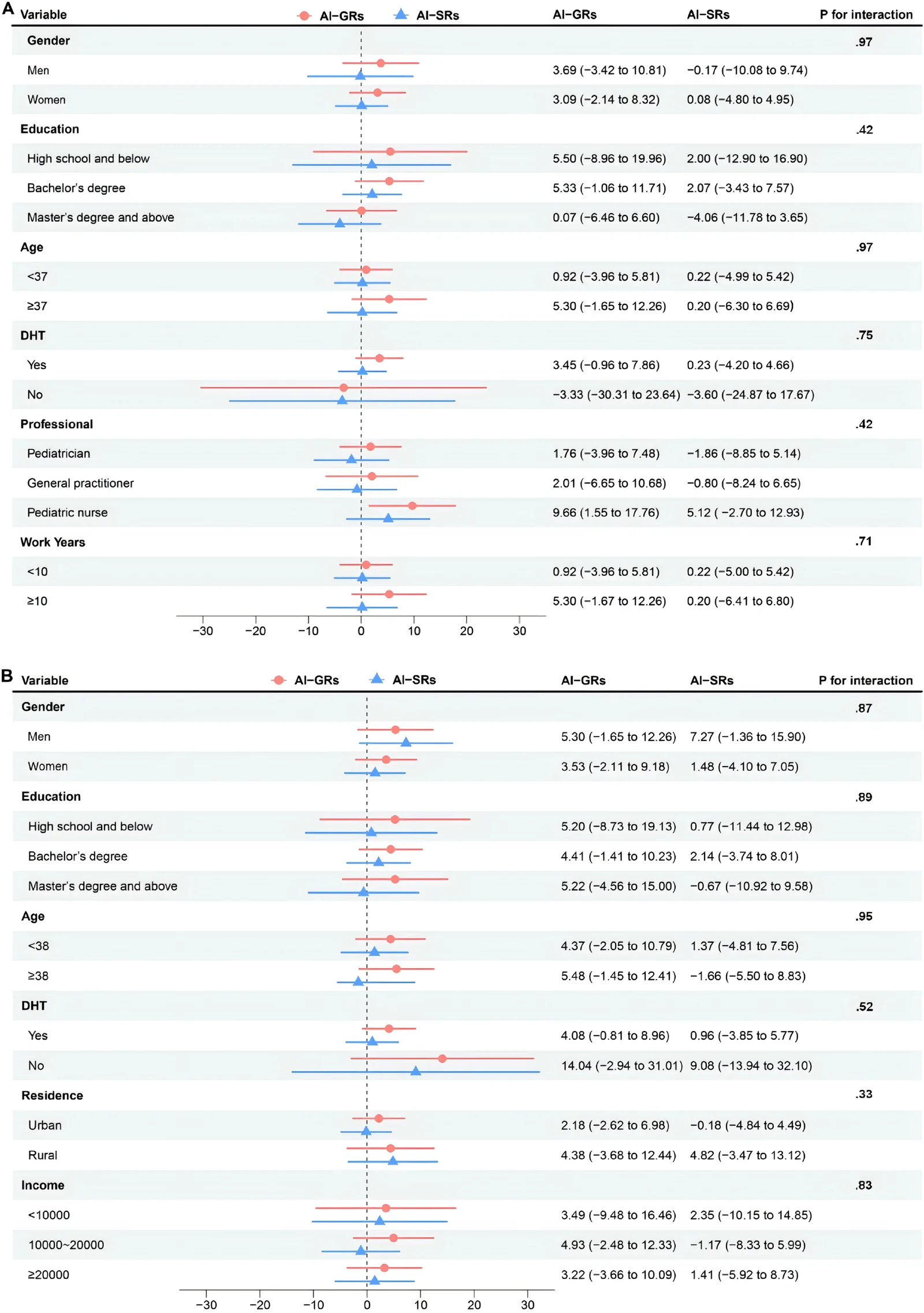
Subgroup analysis of characteristics of respondents and evaluation of 3 health education materials. (A) Subgroup analysis of demographic characteristics of medical professionals and evaluation of 3 health education materials, (B) subgroup analysis of demographic characteristics of pediatric patient families and evaluation of 3 health education materials; “professional” and “work years” refer to medical professionals; “residence” and “income” refer to caregivers. AI-GR: AI-generated material; AI-SR: AI-simplified version; DHT: digital technology use experience; PEAR: published expert-authored response.

Subgroup analyses demonstrated generally balanced overall ratings across demographic strata (Figure 3). Although point estimates suggested potentially higher ratings among respiratory specialty nurses, no significant interaction effects were detected across any subgroups in either population (medical professionals: gender, *P*=.97; education, *P*=.42; age, *P*>.99; digital technology use experience, *P*=.75; profession, *P*=.42; and work years, *P*=.71; pediatric patient families: gender, *P*=.87; education, *P*=.89; age, *P*=.95; digital technology use experience, *P*=.52; residence, *P*=.33; and income, *P*=.83).

### Source Identification

**Figure 4.**
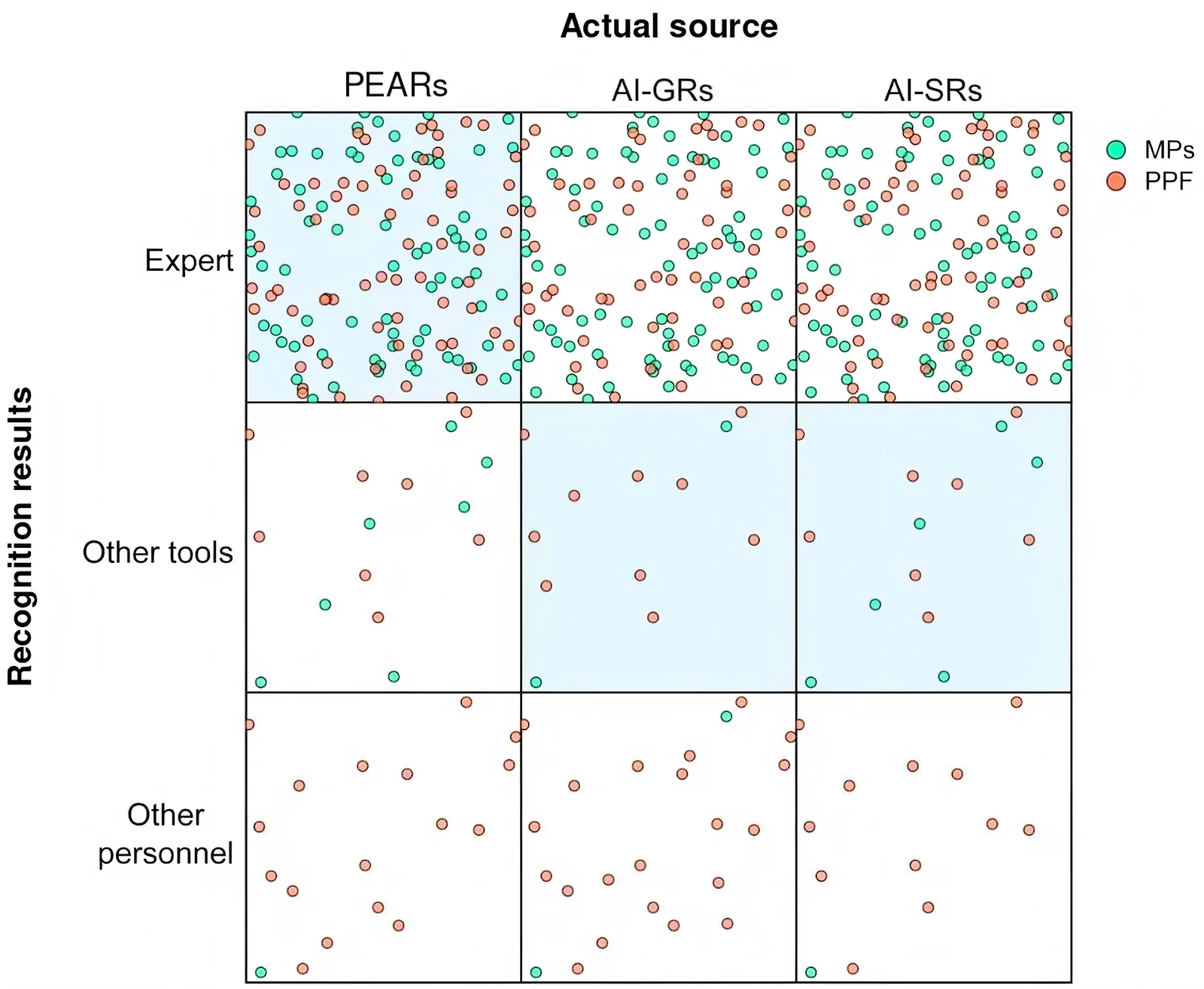
Respondents’ identification of 3 sources of health education materials. Each point represents 1 participant (green: medical professionals [MPs]; orange: pediatric patient families [PPFs]). The horizontal axis represents the actual material source groups, and the vertical axis indicates participants’ selected sources. The light blue squares indicate the correct-response regions: “expert” for published expert-authored responses and “other tools” for AI-generated materials (AI-GRs) and AI-simplified responses (AI-SRs). Those selecting “other personnel” are considered incorrect.

Only 32% (166/519) of all participants correctly identified the material sources. While 80.6% (141/175) of participants in the PEARs group accurately recognized published expert-authored content, merely 7.3% (25/344) in the AI-generated groups correctly identified the content as LLM-generated. Medical professionals (79/243, 32.5%) and patient’s family members (88/276, 31.9%) demonstrated similar correct identification rates (Figure 4).

### Linguistic Evaluation

**Figure 5.**
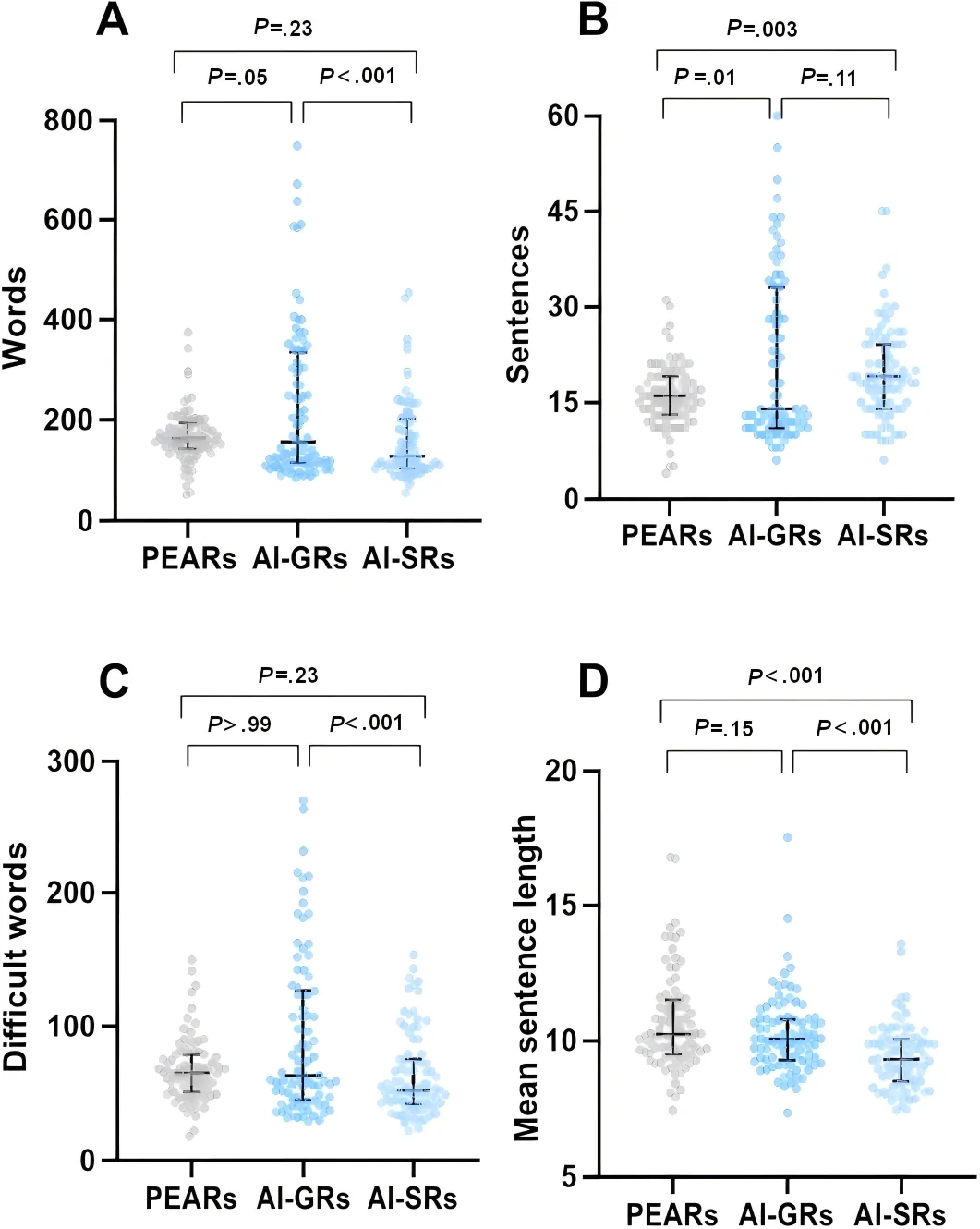
Comparison of linguistic evaluation indicators for 3 sets of health education materials. The panels show (A) word count, (B) difficult word count, (C) sentence count, and (D) mean sentence length. Data are expressed as median (IQR). The *P* values presented are Holm-adjusted *P* values.

Compared with AI-GRs, AI-SRs had fewer words (median 128, IQR 106‐203 vs median 157, IQR 116‐335), fewer difficult words (median 52, IQR 42‐75 vs median 63, IQR 45‐126), and a shorter average sentence length (median 9.33, IQR 8.53‐10.05 vs median 10.07, IQR 9.28‐10.82; all Holm-adjusted *P*<.001). Compared with PEARs, AI-SRs had more sentences (median 19, IQR 14‐24 vs median 16, IQR 13‐19; Holm-adjusted *P*<.001) and a shorter average sentence length (median 9.33, IQR 8.53‐10.05 vs median 10.24, IQR 9.52‐11.50; Holm-adjusted *P*<.001). Between AI-GRs and PEARs, only the difference in sentence count remained significant after Holm adjustment, with AI-GRs having fewer sentences (median 14, IQR 11‐33 vs median 16, IQR 13‐19; Holm-adjusted *P*=.01). No other pairwise differences remained significant after Holm adjustment (Figure 5).

## Discussion

### Principal Findings

This multicenter, randomized, double-blind evaluation study compared Chinese PEARs with AI-GRs and AI-SRs across health care professionals and caregivers. The primary outcome captured participants’ perceived quality, credibility, usefulness, adoption, and overall scores through the information adoption model. AI-GRs received numerically higher overall scores than PEARs in both participant populations, whereas AI-SRs and PEARs had similar numerical scores. After Holm correction, none of the prespecified comparisons between either AI group and PEARs was statistically significant for the overall score or its 4 dimensions. The results showed no statistically detectable differences in the planned comparisons, while the numerical patterns provide a basis for more targeted investigation.

The favorable reception of AI-GRs accords with earlier studies in which LLM responses to asthma and rheumatology education materials were positively evaluated [31,32], as well as with broader evidence identifying patient education as a growing use of general-purpose LLMs [33]. By incorporating blinded assessments from both medical professionals and pediatric patient family members and comparing the materials with an established expert-authored resource, this study extends previous work beyond expert scoring to include the perspectives of intended users.

The modest numerical advantage of AI-GRs may be related to fluency, organization, and perceived accessibility, which can shape readers’ impressions of health information, although these attributes were not separately measured in this study [34-38]. The nominal difference in perceived usefulness among pediatric patient family members was also attenuated after correction across the 16 dimension-specific comparisons (adjusted *P*=.38), suggesting that this pattern should be interpreted cautiously. Overall, the findings are most informative about perceived credibility, relevance, usefulness, and willingness to use the materials. They provide preliminary support for the acceptability of LLM-assisted health education, while objective accuracy, clinical safety, comprehension, and behavioral effects remain complementary domains for purpose-designed evaluation [39].

The AI-SR findings further suggest that linguistic simplification is not captured fully by reductions in word count, difficult words, or sentence length. Although all 3 indices decreased, the scores on the overall scale and 4 dimensions did not increase. Similar work has shown that LLMs can lower the estimated reading level of health information but has also emphasized the importance of outcomes beyond readability scores and continued human oversight [40]. A ceiling effect offers one plausible explanation for the present pattern. When ratings cluster near the upper end of a scale, the limited remaining response range makes additional improvement more difficult to detect [41]. The pediatric patient family sample was predominantly highly educated, urban, and relatively affluent and may therefore have found the original materials readily accessible; if initial acceptance was already high, ratings concentrated near the upper end of the scale may have left limited room for simplification to produce further improvement. At the same time, shortening language may remove contextual detail or weaken coherence, both of which contribute to text difficulty but are rarely incorporated into Chinese readability assessment systems [42-44]. Future studies should examine more diverse health literacy and socioeconomic backgrounds and evaluate readability together with comprehension, factual retention, and the ability to apply information in realistic tasks.

Participants’ judgments of authorship add a further dimension to the interpretation of perceived credibility. Although overall source identification accuracy was 32%, responses were markedly asymmetric: 80.6% (141/175) of participants assigned to PEARs selected a human source, whereas only 7.3% (25/344) of those assigned to the two LLM groups selected an LLM source. This pattern may be consistent with a directional tendency to attribute polished health information to a human expert. Fluent, credible presentation may shape trust before the underlying accuracy and safety have been independently evaluated. Work on transparency and explainability similarly distinguishes subjective trust from appropriately calibrated reliance on an AI system [45]. In patient education, the observed acceptability therefore points most plausibly toward an LLM-assisted workflow in which generated or simplified materials undergo qualified professional review, objective accuracy and safety assessment, clear provenance disclosure, and monitoring tied to the model version and prompt process [46-49].

### Limitations

Several limitations should be considered. PEARs were taken from a polished, committee-validated public resource, whereas the LLM materials were generated for this study and prescreened; these production differences may have influenced the evaluations. The prescreening was conducted by a single pediatric pulmonologist, which may have introduced reviewer-dependent judgment into content selection. Because the source resource was publicly available, possible prior model exposure to it or related material cannot be excluded. The predominantly highly educated, urban, and relatively affluent family sample may limit generalizability to populations with lower health literacy or fewer socioeconomic resources, while the asymmetric source judgments may partly reflect directional response anchoring. The use of 1 GPT-4o version, 1 language, and 1 prompt strategy further limits generalizability to other models and workflows. Finally, although the study received ethics approval before participant recruitment and the study design and outcomes were specified before enrollment, the registration was not formally completed and publicly posted until after participant recruitment had ended. The study was therefore retrospectively registered. Nevertheless, no outcome was added, removed, or reclassified on the basis of the observed results. Future randomized evaluation studies will be registered, where applicable, before enrollment of the first participant.

### Conclusions

This randomized, double-blind study examined medical professionals’ and caregivers’ perceptions of LLM-generated, LLM-simplified, and published expert-authored pediatric asthma education responses. After Holm correction, the prespecified analyses did not identify statistically significant differences in overall information adoption model scores or the 4 information adoption model dimensions. Linguistic analyses indicated that AI-based simplification reduced word count, difficult-word count, and average sentence length. These findings provide preliminary evidence regarding the use of generative AI for developing and simplifying pediatric asthma education materials. Further research is needed to assess their clinical accuracy, accessibility, real-world effectiveness, and applicability across languages, populations, and health conditions.

## Supplementary material

10.2196/98118 Multimedia Appendix 1 Generation procedure and exact prompts.

10.2196/98118 Multimedia Appendix 2 Pre-evaluation screening of the AI response.

## Abbreviations

AI-GR: AI-generated response
AI-SR: AI-simplified response
LLM: large language model
PEAR: published expert-authored response
