# Supplementary material for: Rethinking Pediatric Asthma Education Through Large Language Model Generation and Simplification: Randomized Double-Blind Study
Source: J Med Internet Res. 2026 Aug 26;28:e98118. doi: 10.2196/98118 (PMC13514414; doi:10.2196/98118)
Supplement: Supplementary file 1 [file jmir-v28-e98118-s001.docx]

### Generation Procedure and Exact Prompts

All materials were generated in Chinese through the ChatGPT([https://openai.com/blog/chatgpt](https://openai.com/blog/chatgpt)）.web interface using GPT-4o between August 13 and August 29, 2024. A separate new conversation was initiated for each asthma health management question to prevent contextual information from previous questions from influencing subsequent outputs. For each question, the AI-generated response (AI-GRs) prompt was submitted only once. After the AI-GRs was produced, the AI-simplified response (AI-SRs) prompt was submitted once as a follow-up instruction within the same conversation, so that the AI-SRs was generated directly from the corresponding AI-GRs. All prompts and all corresponding outputs were entered and generated entirely in Chinese.

**Prompt for AI-Generated Responses (AI-GRs)**

【我是一名哮喘儿童患者家属，请回答以下关于哮喘健康管理的问题，以满足我的阅读需求。回答完所有问题后，请检查并纠正任何临床逻辑错误，问题：...】

[Acting as a family member of a child with asthma,please answer the following questions on asthma healthmanagement to meet my reading needs.After answering allquestions, please check for and correct any clinical logicerrors]

**Prompt for AI-Simplified Responses (AI-SRs)**

【请对上一轮回复的内容进行语言优化，使其符合健康教育材料开发的要求，并尽可能易读，同时仍需保持专业准确性。】

[Please do linguistic optimization of the content of the previous round of responses so that the content of the responses meets the requirements for the development of health education materials and is as readable as possible,but still needs to be balanced with professional accuracy.]

**AI-Generated Responses, AI-GRs：**

**问题1什么是CCAAP？为什么要使用CCAAP？**

CCAAP（Chronic Care Action and Assessment Plan）是一种针对慢性病患者（如哮喘）的综合管理工具，旨在改善患者的健康结果。它通常包括详细的评估、个体化的护理计划、监测策略和患者教育内容，以帮助患者和家庭更好地管理病情。

为什么要使用CCAAP？

①个体化管理：CCAAP根据患者的具体病情、生活方式和需求制定个性化计划，从而提高治疗效果。

②系统化监测：通过标准化的评估和监测工具，医生和患者可以更有效地追踪病情变化，及时调整治疗方案。

③增强患者参与：CCAAP强调患者和家庭在健康管理中的积极参与，帮助患者了解自身病情、管理症状和使用药物。

④预防并发症：通过系统化的健康管理，CCAAP有助于识别潜在的健康风险，减少急性发作和住院的可能性。

⑤提升健康教育：CCAAP通常包括教育材料和指导，帮助患者理解哮喘的发病机制、诱因及日常管理技巧。

⑥跨专业合作：CCAAP促进医生、护士、药师和其他医疗专业人员之间的合作，确保患者获得全面的支持。

使用CCAAP能够为哮喘患者提供系统化、个体化的健康管理，增强患者的自我管理能力，降低疾病急性发作的风险，从而改善整体生活质量。

**问题2 CCAAP手机版应用程序有哪些优点？**

CCAAP 是“中国儿童哮喘行动计划”（China Children’s Asthma Action Plan）。手机版将纸质行动计划、症状记录和呼气峰流量监测等功能整合到手机中，主要有以下优点：

随时查看个体化方案：家长可以在手机上查看医生制定的日常控制及急性发作处理方案，不易丢失。

①“红—黄—绿区”简单直观：根据孩子的症状和呼气峰流量判断当前状态。绿区通常表示控制良好，黄区提示病情可能加重，红区提示危险、需要尽快就医。

②便于连续监测：可记录症状、呼气峰流量以及可能的诱发因素，帮助家长发现病情变化，而不只是等到明显喘息后再处理。

③辅助规范用药：根据医生预先制定的行动计划提示相应处理措施，有助于减少漏药、随意停药或处理不及时。

④方便复诊沟通：连续记录的数据可以帮助医生了解孩子一段时间内的控制情况，从而更有依据地评估和调整治疗。

⑤提供健康教育：家长可以更方便地学习哮喘、吸入治疗和日常管理知识，提高家庭自我管理能力。

⑥携带和记录更方便：与纸质计划相比，手机通常随身携带，数据也更容易长期保存和回顾。

需要特别注意：CCAAP 手机版是辅助管理工具，不能代替医生诊断，也不应根据 App 提示自行长期增减控制药物。出现明显呼吸困难、说话或进食困难、口唇发紫、精神状态异常，或者使用缓解药后仍无改善时，应立即就医或呼叫急救。孩子的红黄绿区标准和具体用药必须由医生结合其病情预先制定。

**问题3 什么是哮喘？**

哮喘是一种慢性炎症性疾病，主要影响气道，导致气道对多种刺激的反应性增加。其特征包括反复发作的喘息、气促、胸闷和咳嗽，尤其是在夜间或清晨症状更为明显。哮喘的发作通常与气道的炎症和收缩有关，可能由过敏原、污染物、感染、运动、冷空气或情绪波动等诱因触发。

①病因：哮喘的病因复杂，既包括遗传因素，也包括环境因素，如空气污染、过敏原（如尘螨、花粉、宠物毛发）和感染。

②症状：常见症状包括喘息、胸闷、咳嗽（尤其在夜间）和呼吸困难。不同患者的症状和严重程度可能有所不同。

③诊断：诊断通常基于病史、临床症状、体格检查以及肺功能测试（如峰流速计和肺活量测定）。

④管理：哮喘管理包括避免触发因素、定期监测症状和肺功能、使用吸入性药物（如类固醇和支气管扩张剂）以及制定应急计划。

⑤预防：教育患者及其家属如何识别并管理哮喘诱因，保持良好的生活习惯，以减少发作风险。

**问题4 哮喘可以遗传吗？**

①家族史：如果父母或兄弟姐妹中有人患有哮喘，孩子的风险会显著增加。这个风险可能与遗传因素有关，如基因突变和免疫系统反应。

②环境因素：虽然遗传因素在哮喘的发病中起到重要作用，但环境因素也不可忽视。过敏原（如花粉、尘螨）、空气污染、烟草烟雾等都可能诱发或加重哮喘症状。

③基因与环境交互作用：哮喘的发生往往是遗传因素与环境因素共同作用的结果。例如，某些基因可能使个体对特定过敏原更加敏感，尤其在接触这些过敏原时，哮喘的风险更高。

④预防与管理：虽然遗传因素不能改变，但通过早期识别、避免已知诱因和进行适当的治疗，可以有效管理哮喘，提高生活质量。

综上所述，哮喘确实具有一定的遗传性，但其发生和发展还与环境因素密切相关。对于有家族史的个体，及早的健康监测和积极的管理尤为重要。

**问题5儿童哮喘有哪些类型？**

①过敏性哮喘：这是最常见的类型，通常与过敏原（如花粉、宠物毛发、尘螨等）相关联。在接触这些过敏原后，儿童会出现哮喘症状。

②非过敏性哮喘：此类型不主要由过敏原引发，可能与病毒感染、空气污染、运动、天气变化等因素有关。许多儿童在呼吸道感染后会出现哮喘症状。

③运动诱发性哮喘：这种哮喘通常在运动后发作，尤其是在冷空气或干燥空气中运动时更为明显。

④夜间哮喘：一些儿童在夜间或清晨时会出现哮喘症状，这可能与生理节律变化或过敏反应有关。

⑤职业性哮喘：虽然在儿童中相对少见，但某些情况下儿童可能因接触特定的职业性刺激物（如化学品、粉尘等）而引发哮喘症状。

⑥难治性哮喘：一些儿童的哮喘症状难以通过常规治疗控制，可能需要更复杂的治疗方案。

**问题6 儿童哮喘与成人哮喘有何不同和联系？**

儿童哮喘与成人哮喘在病理生理、临床表现、治疗方法和管理策略上存在一些不同，同时也有很多联系。以下是它们之间的主要区别和联系：

区别

（1)发病机制：

①儿童哮喘：通常与过敏反应有关，常见的过敏源包括花粉、尘螨、宠物毛发等。儿童的气道较小，易于出现气道阻塞和炎症。

②成人哮喘：可能与环境因素、职业暴露、吸烟等有关。成人的哮喘症状往往与气道重塑（长期变化）相关。

（2）临床表现：

①儿童哮喘：症状多为喘息、咳嗽（尤其是夜间或运动后）、胸闷等，可能会表现出更明显的周期性。

②成人哮喘：症状可以更复杂，包括持续的咳嗽和气短，可能伴随其他呼吸系统疾病。

（3）药物治疗：

①儿童哮喘：药物剂量通常根据体重调整，且需特别注意药物对儿童生长的影响。

②成人哮喘：治疗方案较为灵活，可能包括更强效的抗炎药物，且患者通常有更多的自主权和管理能力。

（4）心理社会因素：

①儿童哮喘：可能会影响儿童的学校表现和社交活动，家长的管理和教育至关重要。

②成人哮喘：心理因素如焦虑和抑郁可能影响疾病的管理和治疗效果。

联系

（1）病理生理：儿童和成人的哮喘均涉及气道的慢性炎症、气道高反应性和气流受限等基本机制。

（2）治疗目标：无论是儿童还是成人，治疗的主要目标是控制症状、减少急性发作的频率、改善生活质量，并维持正常的肺功能。

（3）急性发作：无论是儿童还是成人，哮喘急性发作的管理原则相似，均需使用支气管扩张剂和可能的糖皮质激素治疗。

（4）教育和自我管理：对患者及其家属进行教育、制定行动计划以及使用吸入器等自我管理工具对于两者均非常重要。

**问题7儿童哮喘有哪些临床特点？**

（1）症状表现：

喘息：经常性出现的喘息声，尤其在夜间或清晨加重。

咳嗽：通常是干咳，可能在活动后、夜间或接触过敏源时加重。

呼吸困难：活动时易感到呼吸急促，重度发作时可能影响日常生活。

胸闷感：部分儿童可能会有胸部紧缩感。

（2）发作特征：

周期性：哮喘症状往往呈周期性发作，有时与过敏源、病毒感染或运动相关。

夜间加重：许多儿童在夜间症状加重，可能影响睡眠。

（3）诱发因素：

过敏源：如尘螨、花粉、动物皮屑等。

环境因素：寒冷空气、烟雾、空气污染等。

感染：上呼吸道感染，尤其是病毒感染。

情绪波动：剧烈情绪波动或过度兴奋也可能诱发哮喘发作。

（4）生理特点：

肺功能变化：通常表现为可逆性气道阻塞，通过支气管扩张剂可改善。

过度反应性：儿童气道对刺激物的反应性增强，表现为气道的高反应性。

（5）家族史：

很多儿童有过敏性疾病或哮喘的家族史，遗传因素在哮喘的发生中起着重要作用。

（6）合并症：

儿童哮喘常与过敏性鼻炎、湿疹等过敏性疾病共存。

在管理儿童哮喘时，家长和医务人员应重视以上临床特点，以便于早期识别和有效控制疾病，确保儿童的生活质量。

**问题8儿童哮喘如何诊断？**

儿童哮喘的诊断通常包括几个关键步骤。首先，医生会进行详细的病史询问，了解孩子的症状（如喘息、咳嗽、呼吸急促和胸闷）以及发作频率和诱因。其次，进行体检以评估呼吸道的状态。接着，可能会进行肺功能测试，如呼吸峰流速测量或肺活量测试，尤其是在孩子能够配合的情况下。此外，医生可能会使用过敏原测试和影像学检查来排除其他疾病。最终，结合症状、病史和测试结果，医生会作出诊断。确保定期随访和评估，调整管理方案。请确认以上信息是否符合您的需求。

**问题9婴幼儿期的反复喘息会发展为哮喘吗？**

婴幼儿期的反复喘息确实可能与后续哮喘的发生相关。许多研究表明，早期的喘息症状，尤其是在婴幼儿时期，可能是哮喘发展的一个风险因素。然而，并不是所有有喘息症状的婴幼儿都会发展为哮喘，影响因素包括：

（1）喘息的性质：如果喘息是由病毒感染引起的（如感冒），而不是由过敏或其他因素引起的，婴儿可能会在长大后不再有喘息问题。

（2）家族史：有家族哮喘或过敏史的婴幼儿更有可能发展为哮喘。

（3）环境因素：如接触过敏原（尘螨、宠物毛发、烟雾等）可能增加哮喘的风险。

（4）其他健康问题：如过敏性鼻炎、湿疹等也可能与哮喘的发生相关。

为了有效管理婴幼儿的喘息，建议：

定期就医：与专业医生合作，监测症状的发展。

避免触发因素：尽量减少接触已知的过敏原和刺激物。

健康教育：家长应了解哮喘的早期迹象和管理策略。

总的来说，反复喘息可能预示着未来哮喘的风险，但个体情况因人而异。定期监测和适当的健康管理非常重要。

**问题10 6岁以下儿童的哮喘如何诊断？**

（1）病史收集：询问孩子的症状，包括喘息、咳嗽、呼吸急促和胸闷，尤其是在夜间或运动后加重的情况。了解症状的频率和持续时间，以及是否有家庭哮喘或过敏病史。

（2）体格检查：医生会检查孩子的呼吸音，寻找喘鸣音或其他异常音。观察是否有呼吸困难的迹象，例如鼻翼扇动或胸部凹陷。

（3）症状监测：使用日记记录孩子的症状、使用的药物以及潜在的触发因素（如过敏原、运动或感染）。

（4）肺功能测试：对于6岁以下的儿童，肺功能测试可能有限，但可以通过呼吸流量测定或使用儿童专用的肺功能仪器来评估气道的通畅度。

在某些情况下，医生可能会使用支气管舒张剂来评估气道反应性。

（5）过敏原检测：可考虑进行皮肤点刺测试或血液检查，以评估是否存在过敏原。

（6）排除其他疾病：鉴别其他可能导致类似症状的疾病，如感染、支气管炎、异物吸入等，确保哮喘的诊断准确。

（7）试用哮喘药物：在某些情况下，医生可能会在观察期内给予支气管扩张剂，并评估症状是否改善，以支持哮喘的诊断。

在诊断过程中，与儿童的家长或监护人沟通非常重要，以确保对症状的描述准确，并制定适当的管理计划。诊断哮喘需要综合考虑多个因素，并在专业医生的指导下进行。

**问题11什么是CVA？**

CVA（Chronic Variable Asthma）是一种特殊类型的哮喘，其特点是患者在症状发作时不伴有典型的喘息声。诊断标准通常包括：1) 反复的呼吸困难和胸闷，2) 肺功能检查显示可逆性气流受限，3) 排除其他呼吸系统疾病。治疗规范包括使用吸入性糖皮质激素（ICS）和长效β2-激动剂（LABA）进行控制，必要时加用白三烯受体拮抗剂和生物制剂。患者还需定期监测症状及肺功能，确保治疗的有效性与调整。

**问题12儿童哮喘要注意与哪些疾病相鉴别？**

儿童哮喘需要与以下疾病进行鉴别：

（1）过敏性鼻炎：常伴有鼻塞、流涕等症状，可能导致误诊。

（2）支气管炎：急性或慢性支气管炎的咳嗽和喘息可能与哮喘相似。

（3）肺炎：细菌或病毒性肺炎可引起呼吸急促和咳嗽，需通过影像学检查确认。

（4）慢性咳嗽综合征：主要症状为持续咳嗽，可能由其他病因引起。

（5）气道异物：特别是在小儿童中，需考虑气道阻塞的可能性。

（6）心脏病：某些心脏病可导致喘息和呼吸困难，需综合评估。

（7）胃食管反流病（GERD）：可导致咳嗽和喘息，需要注意症状的关联性。

在临床评估时，了解病史、症状特征和进行必要的检查是关键，以确保准确诊断和有效管理。

**问题13哮喘的并存疾病有哪些？**

哮喘常见的并存疾病包括过敏性鼻炎、食物过敏、湿疹、鼻息肉、慢性支气管炎和胃食管反流病。研究显示，约60%至80%的哮喘患者同时患有过敏性鼻炎，此外，湿疹在儿童哮喘患者中的发生率高达30%至50%。这些并存疾病不仅影响患者的生活质量，还可能加重哮喘症状，因此综合管理非常重要。对于临床决策，应考虑这些并存疾病，以制定更有效的治疗方案。

**问题14 AR对哮喘有什么影响？**

变应性鼻炎（AR）与哮喘之间有密切的关系。AR可导致上呼吸道的炎症，从而促进气道的过敏反应，增加哮喘发作的风险。此外，AR患者常常会有气道高反应性，这可能导致哮喘症状加重。因此，控制AR是管理哮喘的关键，通常需要使用抗组胺药、鼻用类固醇等治疗手段。有效管理AR不仅能改善鼻部症状，还能减少哮喘的频率和严重程度。

**问题15儿童哮喘的常见检查有哪些？**

儿童哮喘的常见检查包括以下几种：

（1）病史评估：详细询问患者的症状、发作频率、诱因、家族史等，了解哮喘的严重程度和控制情况。

（2）体格检查：检查儿童的呼吸音、呼吸频率、心率及有无喘息、呼吸困难等体征。

（3）肺功能测试：

①峰流速测定（PEF）：儿童在家中可以使用峰流速计定期监测呼吸功能。

②spirometry（肺活量测定）**：通常在儿童年龄大于5岁时进行，评估用力呼气时的肺活量和呼气流量。

（4）支气管激发试验：用于评估气道反应性，通常在肺功能测试结果正常但临床高度怀疑哮喘的情况下进行。

（5）过敏原检测：皮肤点刺试验或血液检查，以识别可能引发哮喘的过敏原，如花粉、尘螨、动物皮屑等。

（6）影像学检查：在某些情况下，可能会进行胸部X光片检查，以排除其他可能的呼吸系统疾病。

（7）呼气一氧化氮（FeNO）检测：测量呼气中一氧化氮的水平，评估气道炎症情况，帮助判断哮喘控制情况。

这些检查结合病史和临床表现，可以帮助医生准确诊断和管理儿童哮喘。

**问题16为什么要测PEF？**

测量峰流速（PEF，Peak Expiratory Flow）在儿童哮喘的健康管理中具有重要意义，主要原因包括以下几点：

（1）早期发现疾病变化：PEF可以帮助监测哮喘的控制情况。当儿童的气道状况变化时，PEF值可能会下降，提示可能出现了哮喘加重的风险。及早发现这些变化可以帮助及时调整治疗方案，防止症状恶化。

（2）个体化治疗调整：通过定期测量PEF，医生和患者家庭可以更好地了解病情变化，制定个体化的管理计划。这有助于确定何时需要增加药物剂量或采取其他治疗措施。

（3）提高患者依从性：让患者及其家庭参与到PEF的监测中，可以增强他们对哮喘管理的理解和责任感。这种参与感可以提高患者遵循治疗方案的意愿，帮助他们更好地控制病情。

（4）识别诱因：通过记录PEF变化与环境因素、活动或其他诱因之间的关系，患者和家属可以更好地识别可能的触发因素，从而采取预防措施。

（5）评估治疗效果：PEF的变化可以作为治疗效果的重要指标，帮助医生和患者评估当前治疗方案是否有效，并在需要时进行调整。

（6）教育和自我管理：PEF测量提供了一个可量化的指标，使得患者和家庭能够在日常生活中更好地理解和管理哮喘，提高自我管理能力。

总之，测量PEF是一种简单、有效的工具，有助于监测和管理儿童哮喘，确保患者在日常生活中能够保持良好的健康状态。

**问题17什么是PEF的预测值和最佳值？**

在儿童哮喘的健康管理中，PEF（峰流速）是一个重要的监测指标，帮助评估气道的通畅程度和哮喘控制情况。以下是关于PEF的预测值和最佳值的详细解释，适合临床专家和患者家属参考。

（1）PEF的预测值：预测值是根据儿童的年龄、性别和身高计算得出的理想峰流速值。它反映了该儿童在没有哮喘或其他呼吸道疾病时可能达到的最佳肺功能水平。通常，使用标准的预测公式（如Nunn和Jenkins的公式）来确定预测值。

（2）PEF的最佳值：最佳值是指该儿童在特定时间段内（通常是在哮喘稳定控制期间）实际测量到的最高峰流速值。这个值通常会高于或等于预测值，代表儿童在管理良好的情况下的肺功能状态。最佳值可以用作后续监测的基准，以评估哮喘控制的效果。

（3）如何使用PEF值

①监测趋势：定期测量PEF值，并与预测值和最佳值进行比较，观察是否有下降趋势。

②调整治疗：如果PEF值低于预计的最佳值，可能需要调整哮喘管理计划，包括药物治疗、避免诱因等。

③紧急情况：PEF值显著下降（通常低于60%预测值）可能预示着哮喘急性加重，需及时就医。

总结：了解PEF的预测值和最佳值对儿童哮喘的管理至关重要。家属和患者应与医疗提供者密切合作，定期监测PEF，以便及时调整治疗方案，保持儿童的健康状态。

**问题18如何使用PEF仪？**

使用峰流速仪（PEF仪）是一种监测儿童哮喘的重要工具，能够帮助评估肺功能和管理哮喘症状。以下是使用PEF仪的步骤和注意事项，旨在满足临床专家和患者家属的需求：

使用PEF仪的步骤

（1）选择合适的设备：确保峰流速仪适合儿童使用，并且已经经过校准。

（2）准备工作：确保设备清洁，并在使用前检查仪器是否完好无损。

（3）站立或坐直：鼓励孩子保持站立或坐直的姿势，这样有助于更好地呼吸。

（4）深吸气：让孩子深吸一口气，尽量让肺部充满空气。

（5）用力呼气：孩子应立即用尽全力通过PEF仪快速呼气，确保气流尽可能强劲和快速。

（6）记录数据：读取并记录仪器显示的峰流速值（L/min）。建议记录至少三次，并取最高值以确保准确。

（7）定期监测：建立每日监测的时间表，尤其是在早晨和晚上，这样可以更好地了解肺功能的变化。

（8）与医务人员沟通：将监测结果记录下来，定期与医生或哮喘管理团队分享，以便评估病情和调整治疗方案。

注意事项

（1）使用前咨询医生：在开始使用PEF仪之前，建议咨询医生，确保掌握正确的使用方法。

（2）保持一致性：尽量在相同的时间和条件下测量，以获得可靠的数据。

（3）了解个人最佳峰流速：与医生一起确定个体的最佳峰流速值（个人最佳值），用于日常监测。

结论：通过定期使用PEF仪，家长和医生可以更好地掌握儿童的哮喘管理情况，及时发现潜在的急性发作风险，从而采取必要的预防和治疗措施。

**问题19如何判断PEF仪测定的结果？**

判断PEF（峰值呼气流量）仪测定的结果是儿童哮喘健康管理的重要环节，通常可以通过以下步骤进行：

（1）了解PEF的基本概念

①PEF是指呼吸道在强烈呼气时的最大流速，反映了气道的通畅程度。

②PEF的正常值因年龄、性别和身高而异，儿童的PEF标准值通常可以参考相应的生长曲线。

（2）测量准备

①确保PEF仪器已正确校准。

②让儿童保持镇静，确保测量时气道畅通，避免使用影响结果的药物（如吸入性支气管扩张剂）在测量前立即使用。

（3）进行测量

①让儿童站立或坐直，深吸气，然后迅速用力呼气，持续到仪器发出提示音。

②每次测量应重复至少三次，取最大值以提高准确性。

（4）评估结果

①个人最佳PEF值：每个儿童应建立自己的最佳PEF值（通常是健康状态下的PEF记录），作为日常监测的参考。

②绿色区（≥80%）：表示控制良好，儿童的症状较少。

③黄色区（50%-79%）：提示可能存在气道阻塞，需注意症状并考虑调整治疗方案。

④红色区（<50%）：表示严重的气道阻塞，应立即采取措施（如使用救援药物）并寻求医疗帮助。

（5）记录与趋势分析

定期记录PEF结果，观察其变化趋势。

与医生讨论这些数据，以便及时调整哮喘管理计划。

（6）注意事项

①并非所有儿童的PEF波动都意味着哮喘加重，需结合临床症状和其他检查结果综合判断。

②如果出现频繁的黄色区或红色区结果，应考虑可能的触发因素（如过敏原、天气变化等）并与医疗团队沟通。

结论：通过定期监测PEF并结合临床表现，可以有效管理儿童哮喘，及时发现病情变化，调整治疗策略，提高生活质量。

**问题20常用的儿童哮喘临床评估工具有哪些？如何选择使用？**

儿童哮喘的临床评估工具对于诊断、管理和监测哮喘的严重程度及控制情况至关重要。常用的评估工具主要包括以下几种：

（1）哮喘控制问卷（ACQ）：适用于5岁以上的儿童，通过评估过去一周的症状和使用的药物来判断哮喘控制水平。

特点：简便易用，适合日常监测。

（2）儿童哮喘控制测试（ACT）：适用于4至11岁儿童的评估工具，通常由家长和儿童共同完成。

特点：帮助判断哮喘控制情况，并可用于评估治疗效果。

（3）哮喘严重性评分（Asthma Severity Classification）：评估儿童哮喘的严重程度，包括症状频率、夜间觉醒、急救药物使用频率等。

特点：可以帮助医生制定个体化治疗方案。

（4）峰流速计（Peak Expiratory Flow Meter）：通过测量呼气峰流速来监测气道通畅程度，适合5岁以上的儿童。

特点：可用于自我监测，帮助识别哮喘恶化的早期迹象。

（5）哮喘管理计划（Asthma Action Plan）：虽然不是传统的评估工具，但它可以结合上述评估结果，帮助患者和家属了解如何应对不同的症状和急性发作。

特点：个性化的管理策略，促进患者的自我管理。

（6）如何选择使用

选择合适的评估工具时，需要考虑以下几个因素：

①年龄：不同的工具适用于不同年龄段的儿童，确保选择与儿童年龄相符的工具。

症状控制的需求：如果需要长期监测，可以选择哮喘控制问卷或儿童哮喘控制测试；如果急性症状频繁，可以重点使用峰流速计。

②家庭参与：工具的选择应考虑到家长的参与程度，易于理解和操作的工具更有利于家庭的参与。

③医疗资源的可用性：在资源有限的情况下，选择简单易用的工具可能更为实用。

结论：综合运用这些工具能够更全面地评估儿童哮喘的控制情况和管理效果。医务人员应根据儿童的具体情况和家庭的需求，灵活选择和调整评估工具，以达到最佳的哮喘管理效果。

**问题21儿童哮喘如何分期、分级？**

儿童哮喘的分期和分级主要基于症状的频率和严重程度。根据国际哮喘和过敏协会的建议，儿童哮喘可分为以下四个阶段：

（1）间歇性哮喘：症状少于每周两次，夜间症状少于每月两次，肺功能正常。

（2）轻度持续性哮喘：症状每周超过两次但不每日，夜间症状每月出现1-2次，肺功能正常或轻度下降。

（3）中度持续性哮喘：症状每天出现，夜间症状每周一次，肺功能轻度至中度下降。

（4）重度持续性哮喘：症状持续存在，夜间症状频繁发生，肺功能显著下降。

分级管理可以帮助制定个性化的治疗方案，从而改善儿童的生活质量。确保定期评估和监测症状变化是关键。

**问题22儿童哮喘的防治原则是什么？**

儿童哮喘的防治原则主要包括以下几个方面：

（1）识别和避免诱因

①过敏源：如尘螨、花粉、霉菌、动物皮屑等，应尽量减少孩子接触。

②环境因素：避免吸烟、空气污染、冷空气和强烈的气味等可能诱发哮喘的环境。

③运动：在运动前应做好热身，并根据医生建议选择适合的运动方式。

（2）规范用药

①长期控制药物：如吸入性糖皮质激素（ICS），用于日常管理，减少哮喘发作的频率和严重程度。

②急救药物：如短效β2激动剂（SABA），用于缓解急性哮喘症状，需随身携带。

（3）监测病情

①定期监测孩子的肺功能，使用峰流速仪（PEF）评估呼吸状况，记录症状变化。

②了解并教育家长识别早期症状和发作的信号，以便及时采取措施。

（4）教育和支持

①教育患者及其家庭关于哮喘的知识，包括病因、症状、药物使用和应对措施。

②与医生保持良好沟通，定期复诊，及时调整治疗方案。

（5）制定应急计划

①制定并遵循个性化的哮喘管理计划，包含日常管理和急性发作时的处理步骤。

②确保家庭和学校人员了解该计划，以便在紧急情况下采取正确措施。

（6）心理支持

注意孩子的心理健康，必要时提供心理支持和咨询，帮助他们应对疾病带来的情绪压力。

**问题23儿童哮喘可以治愈吗？**

儿童哮喘是一种常见的慢性呼吸道疾病，目前尚无根治的方法。尽管哮喘的症状可能在不同年龄段有所变化，甚至在青春期时有些儿童的症状可能减轻或消失，但这并不意味着哮喘被治愈。

目前的管理方法

（1）长期控制药物：如吸入性糖皮质激素（ICS）是儿童哮喘管理的基础，用于减少气道炎症，控制症状，防止哮喘发作。

（2）急救药物：如短效β2-激动剂（SABA），用于快速缓解急性症状和哮喘发作。

（3）避免诱因：识别并避免可能诱发哮喘发作的过敏原和刺激物，如烟雾、粉尘、花粉、宠物毛发等。

（4）定期随访：儿童应定期就医，监测病情和治疗效果，必要时调整药物。

（5）教育和自我管理：家长和儿童需接受哮喘管理的教育，包括如何使用吸入器、识别发作早期症状、制定应急计划等。

结论：虽然目前无法完全治愈儿童哮喘，但通过有效的管理和治疗，大多数儿童能够控制症状，过上正常生活。定期与医疗专业人士沟通，积极参与管理，对儿童的哮喘病情控制至关重要。

**问题24哮喘控制药物有哪几类？**

哮喘控制药物主要分为两大类：长期控制药物和急救药物。以下是详细分类：

（1）长期控制药物：这些药物旨在减少哮喘症状的发生频率和严重程度，通常需要每天服用。

①吸入性糖皮质激素（ICS）：如氟氯噻吨、布地奈德，能够减轻气道炎症。

②长效β2-激动剂（LABA）：如沙美特罗，通常与ICS联合使用，帮助放松气道平滑肌，延长效果。

③白三烯受体拮抗剂：如孟鲁司特，减少气道炎症并改善症状。

④抗IgE抗体：如奥马珠单抗，适用于特定过敏性哮喘患者，减少过敏反应。

⑤抗IL-5生物制剂：如美克单抗，针对特定类型的哮喘，减少嗜酸性粒细胞。

（2）急救药物：这些药物主要用于缓解突发的哮喘症状，通常在发作时使用。

（1）短效β2-激动剂（SABA）：如沙丁胺醇，迅速舒张气道，缓解喘息和呼吸困难。

使用建议：长期控制药物应遵循医生的处方，定期评估疗效和副作用。急救药物应随时携带，以备急需，尤其是在运动或过敏原暴露前。

**问题25哮喘缓解药物有哪几类？**

哮喘缓解药物主要分为两大类：短效β2-激动剂和抗胆碱药物。这些药物通常用于迅速缓解哮喘症状，特别是在急性发作时。

（1）短效β2-激动剂（SABAs）：

①作用机制：通过激活气道平滑肌上的β2受体，导致支气管扩张，迅速缓解喘息、呼吸急促等症状。

②常见药物：沙丁胺醇（Albuterol）、特布他林（Terbutaline）。

③使用方式：通常通过吸入器使用，效果一般在几分钟内显现，持续时间约4-6小时。

（2）抗胆碱药物：

①作用机制：通过抑制支气管平滑肌的胆碱能神经支配，促进支气管扩张。

②常见药物：异丙托溴铵（Ipratropium bromide）。

③使用方式：通常也通过吸入器使用，主要用于急性发作时的辅助治疗。

除了以上两类药物，患者可能还会使用长期控制药物来预防哮喘发作，比如吸入性糖皮质激素（ICS）和长效β2-激动剂（LABAs），但这些不属于缓解药物的范畴。

**问题26 ICS包括哪些药物？适应证是什么？有哪些不良反应？**

（1）吸入性皮质类固醇是管理儿童哮喘的重要药物，常见的ICS药物包括：

①布地奈德（Budesonide）②氟替卡松（Fluticasone）③氟氯噻噻（Flunisolide）

④美氟氯噻（Mometasone）⑤倍氯米松（Beclomethasone）

（2）ICS主要用于以下适应证：

①长期控制哮喘：用于管理持续性哮喘，降低急性发作频率和严重性。

②减少气道炎症：有效降低气道内的炎症反应，提高肺功能。

③预防运动诱发的哮喘：在运动前使用某些ICS可以预防运动后哮喘症状的发生。

（3）使用ICS时，可能出现的副作用包括：

①局部反应：咽喉刺激或痛感；口腔念珠菌感染（口腔真菌感染）

②全身反应（多见于长期大剂量使用）：生长抑制（在某些儿童中可能出现）；骨密度下降；皮质醇水平抑制；体重增加

③其他：情绪和行为变化（如焦虑、抑郁）；视力模糊或白内障风险增加

在临床使用中，应定期评估儿童的生长和症状控制，并根据具体情况调整药物剂量。

**问题27长期ICS治疗对儿童生长发育有无影响？**

长期使用吸入性糖皮质激素（ICS）治疗儿童哮喘的潜在影响是一个常见的关注点。根据目前的研究和临床观察，长期ICS治疗对儿童的生长发育有以下几点影响：

（1）生长速度：一些研究显示，长期使用ICS可能会轻微影响儿童的生长速度，尤其是在使用较高剂量的情况下。最初的影响可能表现为生长速率的轻微下降，但大多数儿童在停药后通常会恢复正常的生长速率。

（2）剂量依赖性：影响生长的风险通常与ICS的使用剂量相关。低剂量ICS的使用一般不会显著影响儿童的生长，而高剂量可能会导致更明显的影响。因此，医生通常会根据儿童的具体情况选择合适的剂量，以平衡哮喘控制与生长发育的需求。

（3）监测与评估：在长期ICS治疗过程中，医生应定期监测儿童的生长情况，包括身高和体重，以确保及时发现任何潜在的生长问题。同时，医生会根据患者的病情和生长情况调整治疗方案。

（4）临床指南建议：许多临床指南建议在哮喘控制良好的情况下，使用最低有效剂量的ICS，以减少对生长的影响。家长和监护人应与医生密切合作，确保治疗方案的个体化，以优化儿童的哮喘管理和生长发育。

总的来说，尽管长期ICS治疗可能会对儿童的生长有轻微的影响，但大多数情况下，这种影响是可控的，合理使用ICS并进行监测可以确保儿童在良好的哮喘控制下正常生长发育。

**问题28β2受体激动剂包括哪些药物？适应证是什么？有哪些不良反应？**

β2受体激动剂是用于治疗哮喘的常见药物，分为短效和长效两类。以下是详细信息：

（1）β2受体激动剂的分类

①短效β2受体激动剂（SABA）

常见药物：沙丁胺醇（Salbutamol）、特布他林（Terbutaline）。

适应证：用于急性哮喘发作时的快速缓解，通常在运动前预防运动诱发的哮喘。

②长效β2受体激动剂（LABA）

常见药物：福莫特罗（Formoterol）、沙美特罗（Salmeterol）。

适应证：用于长期控制哮喘症状，通常与吸入性皮质类固醇联合使用，防止夜间哮喘发作。

（2）不良反应

β2受体激动剂的一些常见不良反应包括：

①心血管反应：心悸、心率增快（尤其在高剂量或使用短效药物时）。

②颤抖：手抖、肌肉震颤，特别是在开始治疗或调整剂量时。

③头痛：某些患者可能出现头痛。

④低钾血症：高剂量或长期使用可能导致血钾水平下降。

⑤耐药性：长期使用短效β2受体激动剂可能导致效果减弱，增加使用频率。

（3）临床管理注意事项

①监测：定期评估患者的症状控制情况和药物副作用，必要时调整治疗方案。

②联合使用：对于长期控制，应优先考虑与吸入性皮质类固醇联合使用LABA，以降低哮喘急性发作的风险。

③教育：患者及其家属应了解何时使用短效β2受体激动剂，以及何时寻求医疗帮助。

**问题29抗胆碱能药物包括哪些药物？适应证是什么？有哪些不良反应？**

抗胆碱能药物是一类通过阻断迷走神经对支气管平滑肌的影响来帮助扩张气道的药物。以下是抗胆碱能药物的相关信息：

（1）主要抗胆碱能药物：①阿托品（Atropine）②溴化伊帕特罗（Ipratropium Bromide）③噻托溴铵（Tiotropium）④乌美溴铵（Umeclidinium）

（2）适应证

抗胆碱能药物主要用于以下情况：

①慢性阻塞性肺病（COPD）：用于改善呼吸功能和减轻症状。

②哮喘：作为支气管扩张剂，尤其在急性发作或急救中使用（如溴化伊帕特罗）。

③急性支气管痉挛：帮助快速缓解气道狭窄。

④某些类型的呼吸道疾病：如肺炎或其他合并症状的情况下。

（3）不良反应

抗胆碱能药物可能导致以下不良反应：

①口干：由于抑制唾液腺分泌。

②喉咙不适：可能会感到喉咙干燥或不适。

③眼部不适：可能引起瞳孔扩大或眼压升高，尤其在阿托品使用时。

④心率加快：少数患者可能出现心悸或心率异常。

⑤便秘：因肠道平滑肌活动减弱。

⑥尿潴留：在某些情况下可能出现尿流不畅。

**问题30半胱氨酰LTRA适应证是什么？有哪些不良反应？**

半胱氨酰LTRA（如蒙脱石、白三烯受体拮抗剂）在儿童哮喘的健康管理中具有重要的应用。以下是其适应证及不良反应的详细信息：

（1）适应证

①支气管哮喘：适用于患有轻度至中度持续性哮喘的儿童，尤其是当常规吸入型糖皮质激素（ICS）效果不佳或不能耐受时。

②运动诱发性哮喘：可用于预防运动诱发的支气管痉挛。

③过敏性鼻炎：在有过敏性鼻炎的同时患有哮喘的儿童，半胱氨酰LTRA可作为联合治疗的一部分。

④慢性咳嗽：对于因哮喘引起的慢性咳嗽症状，使用半胱氨酰LTRA可改善症状。

（2）不良反应

①常见不良反应：

消化系统症状：如腹痛、恶心、呕吐、腹泻。

中枢神经系统：如头痛、嗜睡或失眠。

皮肤反应：如皮疹、瘙痒。

②少见但严重的不良反应：

精神心理变化：如情绪波动、焦虑或抑郁症状。

过敏反应：如呼吸困难、喉部肿胀、荨麻疹等。

**问题31哪些哮喘患儿可以使用变应原特异性免疫治疗（allergen specific immune therapy，AIT）？**

变应原特异性免疫治疗（AIT）是一种有效的治疗手段，适用于某些儿童哮喘患者。以下是适合使用AIT的儿童哮喘患者的主要标准：

（1）适用人群

①明确的过敏原诱发：患者需经过过敏原检测，确认对特定过敏原（如花粉、尘螨、动物皮屑、霉菌等）存在IgE介导的过敏反应。

②哮喘类型：主要适用于过敏性哮喘，尤其是那些症状与特定过敏原暴露密切相关的患者。

③哮喘控制不佳：AIT适用于那些常规药物治疗（如吸入性皮质类固醇、支气管扩张剂）控制不佳的患者，尤其是症状频繁或严重影响生活质量的儿童。

④年纪要求：AIT通常在5岁及以上的儿童中使用，具体年龄取决于所用的治疗方案和相关指南。

⑤意愿和遵从性：患者和家属需对治疗有良好的理解，并愿意遵循治疗计划，定期就诊和接受监测。

（2）不适用人群

①非过敏性哮喘：对于没有明确过敏原或非IgE介导的哮喘患者，AIT通常不适用。

②严重合并症：存在严重心脏疾病、严重免疫缺陷等合并症的患者应谨慎使用AIT。

③近期重度过敏反应史：有过严重过敏反应史的儿童在开始AIT前需仔细评估风险。

**问题32哪些哮喘患儿可以使用单克隆抗体等生物治疗？**

在儿童哮喘的管理中，生物治疗（如单克隆抗体）通常适用于特定类型和严重程度的哮喘患儿。以下是可以考虑使用单克隆抗体等生物治疗的儿童哮喘患者的一些标准：

（1）过敏性哮喘：对于经过确诊的过敏性哮喘患儿，尤其是那些对特定过敏原（如尘螨、花粉、动物皮屑等）有明显反应的患者，可以考虑使用生物治疗。

（2）持续性中重度哮喘：那些症状频繁且使用常规控制药物（如吸入性糖皮质激素、长效β2激动剂等）仍难以控制的儿童，尤其是需要口服糖皮质激素的患者。

（3）特定生物标志物：例如，血液中的嗜酸细胞计数增加、IgE水平升高等，提示可能适合使用如omalizumab（针对IgE的单克隆抗体）或其他生物制剂。

（4）伴随其他过敏疾病：如特应性皮炎、过敏性鼻炎等，且这些症状影响了哮喘控制的儿童。

（5）年龄限制：目前许多生物制剂对年龄有要求，通常在6岁及以上的儿童中进行使用。

（6）未能达到控制目标：即便接受了适当的药物治疗，症状依然不能得到有效控制的患者。

在决定使用生物治疗时，需结合患者的病史、临床表现和实验室检查结果，由专业医生进行全面评估。生物治疗可以显著改善某些患者的病情，但也需考虑到潜在的副作用和经济成本。

**问题33哮喘发作时应首先使用哪些药物？如何选择给药方式？**

哮喘发作时，首先应使用短效β2-肾上腺素激动剂（SABA），例如沙丁胺醇（Albuterol）。这些药物能够迅速扩张气道，缓解急性症状。具体的给药方式和选择主要取决于患者的年龄、病情严重程度以及可用设备。

（1）药物选择

①短效β2-肾上腺素激动剂（SABA）：是首选药物，通常在哮喘发作时使用。

②吸入型糖皮质激素（ICS）：对于一些中度至重度发作的患者，可能需要配合使用，但通常在SABA后使用。

（2）给药方式

①吸入器（MDI）：使用定量气雾剂（Metered-Dose Inhaler）是最常见的方式。需配合使用气雾剂空间器，以帮助药物更好地进入气道。

②干粉吸入器（DPI）：适合能够配合使用的儿童，使用时需吸气用力。

③雾化器：对于小儿或重度发作的患者，雾化器是一个有效选择，能够提供持续的药物吸入，适合那些难以使用吸入器的患者。

（3）临床考虑

①年龄和协作能力：对于年幼儿童，雾化器可能是更好的选择；而较大儿童可以使用MDI或DPI。

②病情严重程度：如果哮喘发作较重，可能需要更高剂量或更频繁使用SABA，医生会根据情况调整治疗方案。

③监测：发作后，患者应监测症状改善情况，如没有改善，需立即就医。

**问题34什么是吸入治疗？有何优、缺点？**

吸入治疗是通过呼吸道直接将药物送入肺部的一种治疗方法，常用于管理哮喘及其他呼吸系统疾病。药物可以是抗炎药（如吸入性糖皮质激素）或支气管扩张剂（如β2-激动剂），其主要目的是减轻气道炎症、扩张气道和改善呼吸功能。

（1）吸入治疗的优点

①直接作用于肺部：吸入药物能够直接到达病灶，快速发挥作用，减轻症状。

②副作用较少：与口服药物相比，吸入药物的全身性吸收较少，因此副作用相对较小。

③迅速缓解症状：急性发作时，快速吸入支气管扩张剂可以迅速改善呼吸困难。

④便于剂量调整：医生可以根据患者的需要灵活调整药物剂量和使用频率。

（2）吸入治疗的缺点

①技术要求：正确的吸入技术对药物的效果至关重要，许多患者（尤其是儿童）可能无法正确使用吸入器。

②依赖性：需要定期使用，有时可能导致患者对药物产生依赖。

③可能的局部副作用：长期使用吸入性糖皮质激素可能导致口腔真菌感染或声音嘶哑等局部副作用。

④经济负担：某些吸入设备和药物可能较为昂贵，对患者家庭造成经济压力。

**问题35如何选择吸入装置？**

（1）吸入装置的类型

常见的吸入装置包括：

①喷雾吸入器（MDI）：适用于大多数儿童，但需要良好的协调能力。

②干粉吸入器（DPI）：适合能够吸入强度足够的儿童，但通常不适合低于5岁的儿童。

③雾化器：适用于无法使用其他装置的幼儿或重症患者，能够提供较大剂量的药物。

（2）年龄和发展阶段

①年龄：通常，5岁以下的儿童建议使用雾化器或MDI配合空间器；而6岁及以上的儿童可逐步使用DPI。

②发展能力：评估儿童的手部协调能力和吸入技巧。

（3）使用便利性

①设备的易用性：选择简单易用的装置，尤其是对于家庭护理者，确保他们能够正确使用。

②维护和清洁：了解吸入装置的维护要求，选择易于清洁的装置。

（4）药物类型

①药物配方：确保所选装置与所需药物兼容，例如某些药物只适用于MDI。

（5）患者的依从性

①教育与培训：确保患者及其家属接受充分的培训，了解如何正确使用吸入装置。

②个体偏好：考虑儿童的个人偏好，增加其使用吸入装置的积极性。

（6） 临床指导

①随访和评估：定期评估儿童的吸入技巧和药物效果，必要时调整装置或治疗方案。

②专家建议：在选择吸入装置时，咨询儿科医生或哮喘管理专家，以获得个性化建议。

**问题36如何正确使用pMDI？**

（1）准备吸入器：

①轻轻摇动吸入器，确保药物充分混合。

②如果吸入器是新开封的，首次使用前需喷出几次以确保其正常工作。

（2）检查嘴部：

①确保嘴部清洁，没有食物残渣。

②使用之前，建议刷牙并漱口，以减少口腔和喉咙的副作用。

（3）姿势：让孩子站直或坐直，放松身体，以便更好地吸入药物。

（4）装置正确：

①如果孩子无法直接用嘴吸入，可以使用空间舱（Spacer），这样可以提高药物的有效性。

②将pMDI与空间舱连接好，并确保密封。

（5）吸入步骤：

①先将吸入器放在嘴唇之间，形成一个良好的密封。

②启动吸入器（同时按下瓶身），然后迅速深吸气。尽量在吸气的同时按下吸入器，确保药物能进入肺部。

③深吸气后，保持呼吸约5-10秒，然后缓慢呼出。

（6）重复使用：如果需要多次吸入，请等待30秒至1分钟，然后重复以上步骤。

（7）清洁吸入器：定期清洗吸入器（根据厂家说明），确保其正常工作。

注意事项

①监测反应：使用pMDI后，观察孩子的症状变化，如果症状未改善或加重，应及时就医。

②定期检查：确保定期与医生沟通，检查使用技巧及治疗效果。

③药物管理：保持药物的有效期，及时补充所需药物。

**问题37如何正确使用储雾罐？**

（1）准备工作：

①确保储雾罐在使用前经过清洗和干燥。检查药物的有效期。

②如果是新罐，使用前需摇匀并进行一次喷雾测试，确保药物能够正常释放。

（2）保持正确的姿势：

①儿童应坐直或站立，以确保药物能有效进入肺部。

②若儿童较小，建议在成人的帮助下进行。

（3）使用前的准备：

①轻轻摇动储雾罐几秒钟（一般3-5秒）。

②拿掉保护盖，检查喷嘴是否干净。

（4）喷雾器放置：将储雾罐的喷嘴放在儿童嘴巴中，唇部紧闭，或使用面罩覆盖面部，确保密封。

（5）吸气和喷雾：

①儿童在吸气时，同时按下储雾罐的顶部，释放药物。应鼓励儿童深吸一口气。

②持续吸气5-10秒钟，随后再缓慢呼气。

（6）等待时间：如果需要使用多种药物，建议在使用第一个药物后等待至少30秒再使用第二个药物。

（7）清洁工作：使用后，务必将喷嘴清洗干净，并重新盖好保护盖，以避免污染。

（8）观察反应：使用后观察儿童的呼吸情况，确保药物有效，注意是否有任何副作用。

注意事项

①定期检查：定期检查储雾罐的有效期和使用状态，避免使用过期药物。

②教育和练习：为儿童提供适当的教育和练习，确保他们掌握正确的使用方法。

③遵医嘱：始终按照医生的指导使用药物，调整剂量和使用频率。

**问题38如何正确使用准纳器？**

（1）准备工作：

①确保准纳器和药物在室温下，摇匀药瓶（如果是需要摇动的类型）。

②如果是新罐或长时间未使用的罐，需按说明书进行排气，通常是喷出1-2次。

（2）定位：

①孩子应保持坐姿或站立，头部稍微向后仰。

②将准纳器放在嘴唇之间，确保嘴唇紧闭，以免药物泄漏。

（3）使用空间器（如需）：

①对于小孩，推荐使用空间器（或喷雾器），以提高药物的有效性。

②确保空间器与准纳器连接牢固。

（4）吸入药物：

①按下准纳器，让药物喷出，同时让孩子缓慢、深深地吸气（如果使用空间器，可以先吸气再按下准纳器）。

②吸入时，建议让孩子保持深吸气约5秒，然后屏住呼吸。

（5）呼气：孩子可以自然呼气，注意不要立即说话或咳嗽。

（6）重复（如需）：如果医生建议使用多剂量，等待大约30秒后再重复上述步骤。

（7）清洁：使用后，应定期清洁准纳器，以防止堵塞。通常每周用温水冲洗喷嘴，确保干燥。

注意事项

①监测效果：使用后观察孩子的症状变化，记录任何副作用或不适。

②定期复查：定期与医生沟通，确保用药方案的适宜性和效果。

③教育孩子：教导孩子了解哮喘管理的重要性，鼓励他们主动参与治疗过程。

**问题39如何正确使用都保？**

都保（Dulera）是一种常用于儿童哮喘的吸入药物，通常包含两种成分：布地奈德（布地奈德是一种类固醇，具有抗炎作用）和福莫特罗（福莫特罗是一种长效β2-肾上腺素受体激动剂，帮助扩张气道）。正确使用都保对于控制哮喘症状至关重要，以下是使用的具体步骤和注意事项：

正确使用都保的步骤：

（1）摇匀：在每次使用前，轻轻摇晃都保的罐子，以确保药物成分均匀。

（2）准备吸入器：将都保的吸入器从保护壳中取出，确保无杂物，检查喷嘴是否清洁。

（3）呼气：深吸一口气，尽量将空气排出肺部，为吸入药物做好准备。

（4）吸入药物：

①将喷嘴放入嘴中，唇紧闭。

②同时按下喷雾器，深吸入药物，尽量将药物吸入肺部。

③吸气时要尽量慢而深，保持吸入的气流平稳。

（5）屏住呼吸：在吸入后，尽量屏住呼吸5-10秒，以确保药物充分沉积在气道中。

（6）呼气：缓慢呼气，不要直接呼出。

（7）清洁吸入器：使用后，按照说明书的指导清洁喷嘴，避免药物残留影响下次使用。

注意事项：

①遵循医生的处方：严格按照医生的建议和处方使用都保，定期复查，根据病情调整剂量。

②监测哮喘症状：定期记录儿童的哮喘症状，观察用药效果，及时与医生沟通。

③避免误用：都保不应作为急救药物（如阿莫特罗或沙丁胺醇），在哮喘急性发作时应使用医生开具的短效β2-激动剂。

④了解副作用：注意可能的副作用，如喉咙痛、声音嘶哑、口腔霉菌感染等，若出现异常症状应及时就医。

**问题40如何正确使用压缩雾化器？**

（1）准备设备：

①确保雾化器的所有部件（如雾化杯、压缩机、管道和面罩或口含器）清洁、无损坏。

②检查药物是否在有效期内，并根据医生的处方准备好相应的药物剂量。

（2）组装雾化器：按照制造商的说明书将雾化器组装好。确保各个部件连接牢固，避免漏气。

（3）药物准备：

①将药物倒入雾化杯中，通常是药物的推荐剂量。注意不要超过最大容量。

②如果需要，可以加入生理盐水以稀释药物，具体根据医生的建议。

（4）连接电源：将雾化器插入电源，并确保开关处于关闭状态。

（5）佩戴面罩或口含器：根据儿童的舒适度选择使用面罩或口含器。面罩应紧贴面部，确保没有漏气。

（6）启动雾化器：打开雾化器，观察雾化杯是否开始产生雾气。此时，儿童应保持安静，尽量深呼吸，确保药物能够充分进入肺部。

（7）雾化过程：

①雾化过程通常持续约5到15分钟，具体时间取决于药物和设备类型。

②观察儿童，确保他们在整个过程中感到舒适。如果出现不适，及时停用。

（8）结束后清洁：雾化结束后，关闭雾化器，拆卸各个部件。

按照制造商的指示清洗雾化杯和面罩。清洗后彻底晾干，以防细菌滋生。

（9）定期检查设备：定期检查雾化器的功能，确保其正常运作。如果设备出现问题，及时更换或修理。

注意事项

①使用频率：遵循医生的指导，按时使用雾化器。不要自行更改用药方案。

②监测效果：使用雾化器后，观察儿童的症状变化，如有明显改善或恶化，及时与医生联系。

③心理支持：鼓励儿童在使用过程中放松，必要时可以通过玩具或音乐转移注意力。

**问题41吸入技术对哮喘治疗效果有哪些影响？**

（1）药物递送的有效性：吸入治疗是哮喘管理中最常用的方式，包括使用吸入性糖皮质激素（ICS）和支气管扩张剂。正确的吸入技术能够确保药物有效地到达气道，减少全身性副作用。

（2）改善药物吸收：不当的吸入技术可能导致药物吸收不充分。例如，呼气过强、吸气过快或过慢，都可能影响药物在气道中的沉积。

（3）减少副作用：正确使用吸入器可以最大程度地减少药物的副作用。比如，避免全身性吸收而导致的副作用，从而提高患者的依从性和治疗满意度。

（4）提高患者依从性：教育儿童及其家长关于正确的吸入技术可以增强他们的信心，提升他们对治疗方案的依从性。教育内容应包括吸入器的使用方法、清洁和维护等。

（5）监测与评估：通过正确的吸入技术，医疗专业人员可以更准确地评估治疗效果，并根据患者的反应调整治疗方案。例如，评估症状控制和肺功能可以帮助判断药物的有效性。

（6）个体化治疗：不同类型的吸入器（如定量吸入器、干粉吸入器等）对不同年龄段和不同能力的儿童有不同的适用性。选择适合患者的吸入器类型，并确保正确使用，可以显著提高治疗效果。

（7）教育和培训的重要性：医务人员应定期对患者及其家属进行吸入技术的培训，尤其是在就诊时。使用视频演示、模拟吸入器等方法可以提高培训的有效性。

**问题42常见的吸入技术错误有哪些？**

（1）未摇匀药物：对于需要摇匀的气雾剂（MDI），如果未摇匀，药物成分可能不均匀，导致剂量不准确。

（2）未正确使用吸入器：儿童可能未能按照说明正确操作吸入器，例如未能在吸气时按压气雾剂，或未能保持吸入器的正确角度。

（3）吸气速度不当：使用干粉吸入器（DPI）时，儿童可能未能进行足够快速且深的吸气，导致药物无法充分进入肺部。

（4）未屏住呼吸：吸入药物后，未能屏住呼吸几秒钟，可能会减少药物在肺部的沉积。

（5）吸入器未能清洁：吸入器如果未定期清洁，可能会影响药物释放和剂量准确性。

（6）药物未按时使用：未按医生建议的时间表使用药物，例如在症状加重前未预防性使用药物。

（7）吸入器选择不当：未根据儿童的具体情况选择合适的吸入器，可能导致药物效果不佳。

（8）无适当的协助：对于年幼儿童，缺乏成人的协助，可能导致操作不当。

**问题43为什么要对哮喘患儿进行管理？**

（1）减少症状：通过有效的管理，可以显著减少哮喘症状的发生，如咳嗽、喘息和呼吸急促。这有助于提高孩子的生活质量，使他们能够更自由地进行日常活动。

（2）预防急性发作：良好的哮喘管理可以减少急性发作的频率和严重程度，避免不必要的急救和住院治疗。通过定期监测和合理用药，家长和医生可以提前识别并应对潜在的哮喘恶化。

（3）改善肺功能：适当的管理可以帮助维持孩子的肺功能，防止长期的肺部损伤。定期的肺功能检测和适时的治疗调整对儿童的生长发育至关重要。

（4）教育与自我管理：通过管理，患者及其家属可以学习识别哮喘触发因素和症状，并掌握如何使用药物和应对措施。教育是帮助孩子实现自我管理的重要部分，增强他们的独立性。

（5）心理健康：长期的哮喘症状可能对孩子的心理健康产生负面影响。通过有效的管理，可以减少焦虑和抑郁的风险，让孩子在心理上更健康。

（6）社会参与：良好的哮喘控制使孩子能够参与学校活动、运动和社交，避免因哮喘而被限制的情况。这对他们的社交技能和自信心有积极的促进作用。

总的来说，哮喘的管理不仅仅是控制症状，更是全面提升孩子的生活质量和健康状况的重要措施。通过家长、医生和患者的共同努力，能够实现最佳的治疗效果。

**问题44哮喘患儿为什么要定期到医院复诊？多长时间复诊1次？**

（1）病情评估：定期复诊可以帮助医生评估患儿的哮喘控制情况，包括症状的频率、发作的严重程度和使用药物的效果。这有助于及时调整治疗方案，以确保病情得到良好控制。

（2）用药管理：医生可以根据患儿的具体情况，调整药物种类和剂量，确保患儿得到最适合的治疗方案。定期复诊有助于监测药物的副作用以及使用的合规性。

（3）教育与指导：在复诊时，医生可以对家长和患儿进行健康教育，讲解哮喘的管理知识，包括诱因的识别、避免措施及正确的吸入药物使用方法。这些知识对于日常管理至关重要。

（4）监测合并症：哮喘患儿可能会有其他健康问题（如过敏性鼻炎），定期复诊可以帮助医生识别和管理这些合并症。

（5）预防急性发作：通过定期评估和调整治疗，能够降低急性哮喘发作的风险，提高患儿的生活质量。

复诊频率： 复诊的具体频率因患儿的病情而异。一般建议哮喘控制良好的患儿每3-6个月复诊一次。而对于症状不稳定或控制不良的患儿，可能需要每月复诊，以便及时调整治疗方案。

在整个管理过程中，家长应密切关注患儿的症状变化和用药情况，随时与医生沟通，以便在需要时做出相应的调整。

**问题45哮喘患儿复诊的主要内容有哪些？**

（1）病史回顾：

①询问过去一段时间内的症状频率和严重程度，包括咳嗽、喘息、呼吸急促和夜间症状。

②评估是否有急性加重的情况及其诱因，例如过敏原暴露、病毒感染或环境变化。

（2）用药情况评估：

①检查患儿的日常控制药物和急救药物的使用情况，包括使用频率和剂量。

②询问家长对药物的理解和遵从情况，确保患儿正确使用吸入器或其他给药装置。

（3）肺功能检测：

①根据年龄和配合能力，进行峰流速测定或肺功能测试，以评估肺功能状况。

②对于学龄前儿童，可以使用临床症状评估和家庭观察数据。

（4）过敏原检测：如有必要，进行皮肤点刺试验或血液检测，识别可能的过敏原，以便进行有效的环境控制。

（5）环境评估：

①讨论家庭和学校环境中可能的哮喘触发因素，如吸烟、宠物、尘螨和霉菌等。

②提供相关的环境管理建议。

（6）教育与自我管理：

①向家长和患儿提供哮喘管理知识，包括识别症状、使用药物和应对急性发作的策略。

②强调建立个人哮喘行动计划，确保家长和患儿了解如何应对突发情况。

（7）随访计划：

①根据病情稳定性和控制程度，制定下次复诊的时间表。

②在必要时调整药物治疗方案。

在复诊过程中，确保与家长和患儿建立良好的沟通，积极听取他们的反馈，以便更好地管理哮喘症状，提升生活质量。

**问题46哮喘的早期预防措施有哪些？**

（1）环境管理

①避免过敏原：识别并尽量减少儿童接触的常见过敏原，如花粉、尘螨、宠物皮屑和霉菌。

②空气质量：保持室内空气清新，避免烟草烟雾和空气污染。使用空气净化器可以帮助改善室内空气质量。

③湿度控制：维持适当的室内湿度，防止霉菌滋生，推荐湿度在30%-50%之间。

（2）健康教育

①哮喘知识：教育家长和儿童认识哮喘的症状、诱因和应对措施，帮助他们掌握使用吸入器和其他药物的方法。

②自我监测：鼓励定期使用峰流速仪（Peak Flow Meter）监测肺功能，帮助及时发现症状恶化。

（3）定期体检

①专业评估：定期就诊于儿科或呼吸科医生，评估儿童的哮喘控制情况，必要时调整治疗方案。

②疫苗接种：保持疫苗接种的及时性，特别是流感疫苗和肺炎疫苗，降低呼吸道感染的风险。

（4）药物管理

①长期控制药物：在医生指导下，必要时使用长期控制药物（如吸入性糖皮质激素），以减轻气道炎症。

②急救药物：确保家庭备有急救药物（如短效β2激动剂），并了解其使用时机。

（5）生活方式调整

①健康饮食：鼓励均衡饮食，富含抗氧化物和Omega-3脂肪酸的食物有助于减轻炎症。

②适度锻炼：根据医生建议，进行适度的锻炼，增强体质和肺功能，注意运动前的准备活动。

（6）心理支持

情绪管理：注意儿童的情绪变化，提供心理支持，缓解压力与焦虑，这些因素可能会影响哮喘控制。

**问题47哮喘常见的触发因素有哪些？**

（1）过敏原

①尘螨：尘螨是儿童哮喘的常见过敏原，尤其在湿润、温暖的环境中容易滋生。

②宠物皮屑：如猫、狗等动物的皮屑、毛发也是常见的过敏原。

③花粉：春季和秋季，尤其是树木、草和杂草的花粉，可以引发过敏性哮喘。

④霉菌：霉菌孢子，尤其在潮湿环境中，可以引发过敏反应，导致哮喘症状。

⑤蟑螂和其他昆虫的排泄物：这类过敏原在某些地区尤为常见。

（2）呼吸道病毒感染：常见的病毒，如鼻病毒、流感病毒和呼吸道合胞病毒（RSV），常常是哮喘急性发作的触发因素。特别是在感冒或流感季节，儿童的哮喘症状可能更加严重。

（3）空气污染和环境刺激

①空气中的污染物，如二氧化氮、二氧化硫和颗粒物，尤其在工业化城市中更为常见，可能刺激呼吸道，导致哮喘症状加重。

②烟雾，包括香烟烟雾和其他类型的污染烟雾，是已知的强烈哮喘触发因素。

③强烈的气味，如清洁剂、空气清新剂、香水等化学气味，可能引起一些儿童的呼吸道反应。

（4）气候变化和温度波动：突然的温度变化、寒冷的空气或湿度变化可能刺激气道，诱发哮喘症状。

（5）体力活动和运动：运动诱发性哮喘是指通过剧烈运动引发的哮喘症状，尤其在寒冷天气或空气干燥时更加明显。

（6）情绪和心理因素：情绪波动，如压力、焦虑或哭泣等，可能通过加剧呼吸道的炎症反应，诱发哮喘症状。

（7）药物：一些药物，如阿司匹林和其他非甾体抗炎药（NSAIDs），可能会引起一些哮喘患者的症状加剧。

（8）饮食

①食物过敏：例如奶制品、坚果、海鲜等食物可能是过敏反应的触发因素，从而加重哮喘症状。

②食物添加剂：某些食物中的添加剂，如亚硝酸盐、味精等，也可能导致哮喘症状的发生。

**问题48常见的过敏原有哪些？**

（1）尘螨：尘螨是哮喘最常见的过敏原之一，尤其是在潮湿的环境中。尘螨主要栖息在床垫、枕头、沙发、地毯等家庭环境中的尘土中。尘螨的排泄物和尸体含有强烈的过敏物质。

（2）宠物皮屑：宠物（特别是猫、狗）的皮屑是哮喘患者常见的过敏原。宠物的皮屑随着空气传播，容易被吸入，从而引发或加重哮喘症状。

（3）花粉：不同季节的花粉也是常见的过敏原之一，特别是春季和秋季。树木、草类和杂草的花粉会在空气中传播，尤其是在空气干燥和风大的日子里，易引起过敏反应。

（4）霉菌：霉菌孢子是另一个常见的过敏原，尤其在潮湿环境中生长繁殖。室内潮湿的地方，如地下室、浴室等，可能是霉菌的滋生地。霉菌孢子一旦进入呼吸道，就可能引发哮喘。

（5）昆虫过敏原：蚊子、蟑螂、老鼠等昆虫及其排泄物或尸体残骸，也可能引起过敏反应，尤其是在家庭环境中。蟑螂过敏在一些大城市的家庭中较为常见。

（6）食物过敏：虽然食物过敏与哮喘的关系较为复杂，但一些食物，如牛奶、鸡蛋、坚果、海鲜、花生等，可能会导致哮喘症状加重。食物过敏通常通过皮肤测试或血液检查来确诊。

（7）烟草烟雾：吸烟或暴露在二手烟中，会增加儿童哮喘的风险，烟草烟雾中的有害物质可刺激呼吸道，导致哮喘发作或加重。

（8）空气污染：汽车尾气、工业排放、室外空气污染等也是哮喘的重要诱因之一。污染物可以刺激气道，导致呼吸道炎症，进而诱发哮喘。

（9）寒冷空气：冷空气或干冷的气候变化可能引发哮喘症状，尤其是对有过敏性哮喘的儿童来说，寒冷空气可刺激呼吸道，引起痉挛。

**问题49哮喘患儿为何需要进行过敏原检测？**

（1）明确过敏原触发因素：哮喘的发作往往与过敏原密切相关，如尘螨、花粉、动物皮屑、霉菌、某些食物等。通过过敏原检测，可以明确孩子是否对某些过敏源敏感，并识别可能导致哮喘症状加重或发作的具体过敏原。了解过敏原后，家长可以采取有效的预防措施，减少孩子与这些过敏源的接触，从而减少哮喘发作的频率和严重性。

（2）个性化治疗方案：通过过敏原检测，医生能够更精确地了解哮喘患儿的过敏情况，从而制定个性化的治疗计划。例如，对于对某种过敏原高度敏感的患儿，可以采取免疫治疗、避免接触相关过敏原，或根据过敏源选择合适的药物（如抗组胺药、吸入类固醇等），使治疗更加有效。

（3）提高疾病控制率：过敏原是引起哮喘症状加重的重要因素之一。通过定期进行过敏原检测，可以动态监测孩子的过敏反应，及时调整治疗方案，避免过敏原的长期暴露，进而提高哮喘的控制率，减少急性发作和住院的风险。

（4）促进家庭和学校的支持：当知道孩子的过敏原后，家庭成员、学校老师和保健人员可以更好地理解孩子的疾病，避免触发过敏原。例如，避免将宠物带入学校、避免过敏季节外出、调整家庭环境（如更换床单、清理灰尘等）。这些措施能够帮助患儿更好地控制病情，提升生活质量。

（5）提高患者和家属的自我管理能力：过敏原检测能够让家长了解哮喘发作的潜在触发因素，有助于家长更加主动地管理孩子的健康状况。通过教育家长如何识别和避免过敏原，可以增强家属对哮喘管理的信心，使其更好地配合医生的治疗和护理建议。

（6）减少不必要的药物使用：了解过敏原后，可以帮助医生避免使用一些不必要的药物。例如，过敏性鼻炎和哮喘的症状有时会混淆，过敏原检测有助于区分病因，减少误诊和过度治疗。

**问题50过敏原检测的主要方法有哪些？**

（1）皮肤点刺试验（Skin Prick Test, SPT）：皮肤点刺试验是一种常见的过敏原检测方法，通常用于检测过敏源的即时过敏反应。通过将不同过敏原的提取液滴在患者的皮肤上（通常为前臂或背部），然后使用针头轻微刺破皮肤，观察反应情况。

①优点：操作简便，反应快速（通常15分钟内结果可见），对过敏原的检测敏感度较高。

②缺点：对皮肤刺激性较大，可能出现局部过敏反应，且不能用于严重过敏体质的患者。

（2）血清特异性IgE检测（Specific IgE Test）：此方法通过采集血样，检测血液中针对特定过敏原的免疫球蛋白E（IgE）水平。血清IgE检测可广泛应用于多种过敏原，包括空气传播的花粉、尘螨、宠物皮屑等。

①优点：无创伤，适用于皮肤过敏反应过于敏感或不宜进行皮肤试验的患者；可同时检测多种过敏原。

②缺点：需要抽血，结果通常较皮肤点刺试验慢，且某些情况下可能存在假阳性或假阴性。

（3）皮肤划痕试验（Skin Scratch Test）：皮肤划痕试验与皮肤点刺试验类似，但操作时会用小刀或划痕针在皮肤表面划伤后涂抹过敏原溶液。这种方法比较少见，主要用于无法进行皮肤点刺试验的特殊病例。

①优点：对于某些特殊过敏源可能会更有效。

②缺点：需要更多的操作空间，且可能导致更大范围的皮肤损伤。

（4）吸入性过敏原的鼻拭子采样：通过收集患者鼻腔内的分泌物，分析其中的过敏原特异性IgE水平。这种方法较为简便，但更多用于过敏性鼻炎的诊断，不如皮肤点刺试验和血清IgE检测广泛用于哮喘的诊断。

①优点：无创，适合部分不能耐受皮肤试验的患者。

②缺点：对哮喘的诊断效能有限，且需要对鼻腔分泌物进行采集。

（5）食物过敏原检测（Food Allergen Testing）：在某些儿童哮喘病例中，食物过敏可能是诱发因素之一。针对常见食物过敏原（如牛奶、鸡蛋、坚果等），可以使用皮肤点刺试验、特异性IgE检测等方法进行诊断。

①优点：能够帮助识别食物过敏的诱因，尤其对于哮喘合并食物过敏的患者。

②缺点：对于食物过敏的测试通常需要综合多种检测方法进行评估。

**问题51什么是螨虫？如何避免尘螨过敏？**

螨虫（Dust Mites）是微小的昆虫，肉眼无法直接看见。它们通常生活在温暖、潮湿的环境中，尤其是在家庭的床上、枕头、被褥、地毯、窗帘等地方。螨虫本身并不会直接引起过敏反应，但它们的排泄物和尸体中含有过敏原，这些过敏原是导致哮喘和过敏性鼻炎等症状的常见原因。

如何避免尘螨过敏？

尘螨过敏是儿童哮喘的常见诱因之一，采取以下措施可以减少尘螨过敏的风险：

（1）清洁床上用品：每周用60°C以上的热水清洗床单、枕套、被罩等，能够有效杀死尘螨。考虑使用防螨床垫和枕头套，它们可以减少尘螨的滋生。

（2）保持环境干燥：尘螨喜欢潮湿的环境，因此保持室内干燥至关重要。使用除湿机或空调来降低室内湿度，理想湿度应保持在40%至50%之间。

（3）减少地毯和软家具：地毯和软家具容易积累尘螨，因此尽量避免使用地毯，或者定期用吸尘器清洁，并使用有高效过滤系统（HEPA过滤）的吸尘器。

（4）定期清洁空气：使用空气净化器可以减少空气中的尘螨和其他过敏原，尤其是配有HEPA过滤器的空气净化器。定期开窗通风也有助于改善空气质量。

（5）避免积尘区域：尘螨喜欢藏匿在床头柜、窗帘、书架等积尘区域，因此定期清洁这些地方，减少尘土的积累。

（6）保持儿童的室内活动区域清洁：如果孩子有哮喘或过敏史，确保他们的玩具、衣物、床铺等地方保持干净，避免带入过多的尘土。

（7）考虑过敏原测试：如果怀疑尘螨过敏，儿童可以进行皮肤过敏原测试，确认是否对尘螨过敏，并在医生指导下采取相应的防护措施。

**问题52如何避免霉菌过敏？**

霉菌是常见的室内过敏源之一，尤其对于患有儿童哮喘的孩子，霉菌暴露可能会引发过敏反应，甚至加重哮喘症状。以下是一些有效的措施，可以帮助减少霉菌暴露，避免霉菌引起的过敏反应：

（1）保持室内干燥：霉菌喜欢湿润的环境，因此控制室内湿度是预防霉菌过敏的关键。使用除湿器将室内湿度保持在30%-50%之间，避免潮湿空气导致霉菌滋生。

（2）清洁并通风良好：定期清洁家中易积水或潮湿的区域，如浴室、厨房、地下室和窗户边缘。定期打开窗户，保持室内通风良好，可以有效减少霉菌生长的机会。

（3）处理水渍问题：检查房屋是否有漏水现象，修理破损的水管和漏水的屋顶，防止水渍积累。潮湿的墙面或地板是霉菌生长的温床，及时修复这些问题是预防霉菌的关键。

（4）移除霉菌;如果家中已经有霉菌，可以使用含有漂白水的溶液进行清洗，或者购买专用的霉菌清除剂进行处理。清洗时，佩戴口罩和手套，避免直接接触霉菌和吸入霉菌孢子。

（5）使用空气净化器：空气净化器可以帮助清除室内空气中的霉菌孢子。选择具有HEPA（高效颗粒空气）过滤功能的空气净化器，能有效过滤细小的霉菌孢子，改善空气质量。

（6）注意室外霉菌：除室内环境外，霉菌也可能在户外环境中存在，尤其是在湿润的天气中。尽量避免让孩子在潮湿、霉菌较多的地方玩耍，尤其是在雨后或湿度较高的季节。

（7）防止霉菌积聚在床上用品：床单、枕头和被褥是霉菌的常见滋生地，尤其是如果这些物品经常处于潮湿环境中。定期清洗床上用品，使用防水和透气性良好的床垫保护套，有助于防止霉菌生长。

（8）注意玩具和儿童用品的清洁

对于小孩的玩具、书籍等，定期清洁和保持干燥也十分重要。避免让这些物品暴露在潮湿的环境中，尤其是在浴室和地下室。

**问题53哪些宠物毛屑容易引起过敏？**

宠物毛屑是儿童哮喘过敏反应的重要诱因之一。不同类型的宠物，其毛屑和皮屑所含的过敏原有所不同，这些过敏原可以引发哮喘发作或加重哮喘症状。常见的容易引起过敏的宠物毛屑包括：

（1）猫的毛屑： 猫的毛屑是最常见的过敏源之一。猫的皮肤会产生微小的脱落皮屑，这些皮屑含有多种过敏原，如Fel d 1（猫的主要过敏原）。即使猫本身并未脱毛，这些过敏原也会随着空气流动扩散到家中。
（2）狗的毛屑： 狗的毛屑同样是常见的过敏源。狗的毛发、皮屑、唾液和尿液等都含有过敏原，如Can f 1和Can f 2等。某些犬种（例如贵宾犬和雪纳瑞）可能因为毛发较为细腻而更容易产生过敏反应。
（3）小动物（如兔子、仓鼠、豚鼠）的毛屑： 小型哺乳动物的毛屑也可能是过敏原。尤其是兔子、仓鼠和豚鼠等宠物，它们的皮肤和唾液中含有的过敏原（例如Lep d 1）会随毛屑散播，可能引发哮喘和其他过敏症状。
（4）鸟类的羽毛和粪便： 虽然鸟类不像哺乳动物那样有毛发，但它们的羽毛、羽屑、粪便中的过敏原（如Gal d 1）同样可能引发过敏反应。鸟类宠物的笼子和环境中的尘土和羽屑是重要的过敏源。
（5）其他爬行动物和两栖动物： 虽然较少见，但某些爬行动物（如蜥蜴和蛇）和两栖动物（如青蛙）也可能通过它们的皮肤或分泌物释放出过敏原，特别是在其栖息地的环境中，可能引起过敏反应。

减少过敏原暴露的建议：

（1）定期清洁家中的空气和地面，尽量减少宠物毛屑的积聚。
（2）给宠物定期洗澡和梳理毛发，减少毛屑的产生。
（3）在家中设立“宠物禁区”，尤其是儿童的卧室和活动区。
（4）使用空气净化器来过滤空气中的过敏原。
（5）尽量避免儿童接触到过敏源，并在外出后清洁身体、衣物。

**问题54哪些植物与哮喘和过敏性鼻炎有关？**

常见与哮喘和过敏性鼻炎相关的植物

儿童哮喘和过敏性鼻炎常常与空气中的植物过敏原（如花粉）相关。这些过敏原会通过呼吸进入身体，引发免疫系统的过度反应，导致哮喘症状加重或过敏性鼻炎的发生。以下是常见的与这些病症相关的植物：

（1）草本植物

①紫花苜蓿（Ryegrass）：这类草的花粉是过敏性鼻炎和哮喘的常见诱因，尤其在春季和夏季的花粉季节。

②狗尾草（Timothy grass）：这种草的花粉通常在春季和夏季释放，容易引发哮喘和过敏性鼻炎的症状。

③雷公藤（Fescue grass）：主要存在于温带地区，特别是湿润的气候中，其花粉常常引发过敏反应。

（2）树木

①白杨树（Birch trees）：白杨树的花粉是引发过敏反应的常见植物之一，尤其在春季，它的花粉容易刺激呼吸道。

②橡树（Oak trees）：橡树的花粉是哮喘和过敏性鼻炎的常见致敏源，尤其在早春。

③桦树（Willow trees）：桦树的花粉可引发严重的过敏症状，特别是在春季，可能导致哮喘加重。

（3）花卉

①菊花（Chrysanthemums）：菊花是过敏源之一，其花粉和气味可能诱发过敏性鼻炎。

②罂粟（Poppy）：某些罂粟花种类的花粉是已知的过敏原，尤其是对于哮喘患者。

（4）其他植物

①常春藤（Ivy）：常春藤的叶片和花朵中的过敏原可能会通过空气传播，尤其是在潮湿环境中。

②仙人掌科植物（Cactus family）：部分仙人掌科植物也能通过花粉引发过敏反应。

过敏性反应的机制：当这些植物的花粉、芽或其他成分进入人体后，免疫系统会错误地将它们识别为有害物质，启动一系列免疫反应。这些反应释放了组胺等化学物质，导致炎症反应，最终引起哮喘的症状（如呼吸急促、喘息、咳嗽）和过敏性鼻炎的症状（如流鼻涕、鼻塞、打喷嚏）。

如何减少植物过敏原的影响？

（1）避免暴露：避免在植物花粉高峰期外出，尤其是春季和秋季。

（2）保持室内空气清洁：使用空气净化器，定期清洁家中，避免花粉积聚。

（3）注意衣物和皮肤的清洁：外出后要及时换衣和洗澡，清洗皮肤和头发上的花粉。

（4）服用抗过敏药物：如抗组胺药、鼻用喷雾剂等，根据医生指导使用药物管理过敏症状。

在临床管理儿童哮喘和过敏性鼻炎时，除了关注环境过敏原外，还应注意：

（1）过敏原检测：通过皮肤试验或血液检测来确定儿童具体的过敏原类型。

（2）个体化治疗方案：根据孩子的具体过敏原和症状，制定个体化的治疗方案，可能包括药物治疗、免疫治疗等。

（3）家长教育：家长应了解过敏反应的症状及管理方式，并确保儿童按时服药和进行环境管理。

**问题55如何应对花粉过敏？**

花粉过敏是导致儿童哮喘发作的一个常见诱因，特别是在花粉季节。了解如何有效应对花粉过敏，可以帮助减少哮喘症状的发生，并提高儿童的生活质量。

（1）避免接触花粉

①留在室内：在花粉浓度较高的时段（通常是早晨和傍晚）尽量避免让孩子外出。尤其是在春季和秋季，花粉浓度较高时，减少户外活动能有效减少过敏反应的风险。

②关闭窗户：在花粉季节，将家中窗户关闭，尤其是在早晨和傍晚。使用空调时，确保设备内有有效的过滤系统。

③避免花粉较多的环境：如果家庭附近有花卉或树木等高花粉源地，可以选择尽量避免到这些地方活动。

（2）洗净暴露区域

①更换衣物：外出后，立即更换衣物，并将外套等外部衣物清洗，防止花粉残留在衣物上带回室内。

②清洗皮肤和头发：花粉可粘附在头发、皮肤和眼睛周围，外出后建议尽早清洗，尤其是面部和头发，减少花粉接触的机会。

③洗澡：如有花粉暴露，及时洗澡和更换衣物，有助于减少花粉的残留。

（3）药物治疗

①抗组胺药物：抗组胺药物可以有效缓解过敏症状，如打喷嚏、流鼻涕和眼睛痒等。常见的抗组胺药物包括非处方药如氯雷他定（Loratadine）等，使用时应根据儿童的年龄和体重剂量来调整。

②鼻用类固醇喷雾：对于有花粉过敏症状且反复发作的儿童，鼻用类固醇喷雾可以减少鼻腔炎症，有效缓解鼻塞、流涕等症状。

③支气管扩张剂：如果花粉过敏引发哮喘症状（如喘息、呼吸困难等），可以在医生指导下使用支气管扩张剂（如沙丁胺醇）来缓解急性症状。

④免疫治疗：对于症状长期且无法通过药物控制的儿童，免疫治疗（过敏原特异性免疫治疗）可能是一个有效的选择。通过逐步增加花粉过敏原的暴露，来提高儿童的耐受性，减少过敏反应。

（4）监测和控制哮喘

①定期监测呼吸功能：对于已知有哮喘病史的儿童，家长应使用峰流速计定期监测呼吸功能，以便及时发现哮喘症状的变化。如果儿童的峰流速值明显下降，可能表明哮喘加重。

②遵循哮喘管理计划：按照医生的建议，确保孩子按时使用预防性哮喘药物，如吸入型类固醇（ICS）等药物，尤其是在花粉季节前后，避免病情加重。

③及时就医：如果孩子出现频繁咳嗽、喘息、呼吸急促等哮喘症状，及时就医。医生可能会调整药物治疗，或根据病情需要进行更多检查。

（5）改善室内空气质量

①使用空气净化器：使用带有HEPA过滤网的空气净化器可以帮助减少空气中的花粉和其他过敏原，尤其是在花粉季节。

②定期清洁家中环境：保持家庭环境清洁，特别是床单、窗帘、地毯等容易积聚过敏原的地方。定期清洗这些物品，减少过敏原的积累。

（6）饮食与补充

①健康饮食：保持均衡的饮食，增加富含抗氧化物质的食物（如水果、蔬菜等），有助于增强免疫力，减少过敏反应。

②补充益生菌：一些研究表明，益生菌可能对调节免疫系统有帮助，降低过敏性疾病的风险，可以在医生指导下适量补充。

（7）了解和识别过敏症状：

提前识别过敏反应：了解孩子可能出现的过敏症状（如频繁打喷嚏、眼睛痒、鼻塞、呼吸急促等），能够帮助家长尽早采取措施，防止症状加重。

**问题56如何应对蟑螂过敏？**

蟑螂过敏是儿童哮喘的重要诱因之一，尤其是在居住环境潮湿、温暖的地方。蟑螂的体内物质及其排泄物、脱落的外骨骼均可能成为过敏源，导致哮喘症状的加重。应对蟑螂过敏可以从以下几个方面入手：

（1）减少蟑螂暴露

①彻底清洁环境：定期清理家中厨房、餐厅、卫生间等易吸引蟑螂的区域，保持环境的干净整洁，尤其是清理食物残渣和垃圾。

②封闭食物：确保食物存放在密封容器中，避免食物残留吸引蟑螂。

③修复裂缝和孔洞：检查家庭各个角落，封闭墙壁和地板上的裂缝，特别是厨房和浴室的接缝处，防止蟑螂进入。

④使用蟑螂诱杀剂：可以考虑使用环保的蟑螂诱杀器，但应避免使用含有强毒性的化学物质，尤其是在有儿童的家庭中。

（2）过敏源检测与避免接触

①过敏原检测：如果怀疑儿童对蟑螂过敏，可以通过过敏源检测（如皮肤点刺试验或血清IgE检测）确认是否为蟑螂过敏。

②避免接触过敏源：一旦确诊蟑螂过敏，应尽量减少儿童与蟑螂及其排泄物的接触。可以使用空气净化器来过滤空气中的过敏源，尤其是在过敏季节。

（3）药物治疗

①抗组胺药：抗组胺药可以缓解由蟑螂过敏引起的过敏症状，如流鼻涕、打喷嚏和眼睛痒。

②鼻用类固醇喷雾：类固醇鼻喷剂有助于减轻过敏引起的鼻塞、流涕等症状。

③哮喘药物：对于哮喘患者，蟑螂过敏可能引发哮喘加重。此时应根据医生建议使用吸入性激素、支气管扩张剂等药物控制哮喘症状。

（4）家庭环境管理

①提高家庭空气质量：使用空气净化器清除空气中的过敏原，保持良好的通风，特别是在潮湿的季节。

②保持湿度适宜：蟑螂喜欢潮湿环境，因此通过保持家中干燥（湿度保持在30%-50%）可以减少蟑螂滋生的机会。

（5）避免二手烟和污染物

①避免二手烟：吸烟会加剧过敏和哮喘症状，因此家庭成员应避免在室内吸烟。

②减少空气污染物：减少家中或周围环境的空气污染物，特别是工业和交通排放物。

（6）寻求专业帮助：如果儿童的过敏症状持续或哮喘控制困难，应及时寻求医生的帮助。医生可能会建议进一步的过敏治疗、调整药物方案或进行免疫治疗。

**问题57什么是食物过敏？**

食物过敏是指免疫系统对某些食物中的特定成分产生异常反应的情况。正常情况下，免疫系统是用来保护身体免受细菌和病毒的侵害，但在食物过敏患者中，免疫系统错误地将某些食物中的蛋白质视为有害物质，并启动防御反应。这种反应可能导致一系列过敏症状，轻则皮疹和胃肠不适，重则可能引发严重的过敏性反应，甚至危及生命。

（1）食物过敏的常见症状

①皮肤症状：如荨麻疹、皮疹、瘙痒等。

②呼吸道症状：如咳嗽、喘息、呼吸急促或困难。

③消化系统症状：如呕吐、腹痛、腹泻等。

④全身性症状：如脸部肿胀、过敏性休克（即过敏性反应导致的严重血压下降和气道阻塞，严重时可危及生命）。

（2）食物过敏与儿童哮喘的关系：在儿童中，食物过敏与哮喘有一定的关联。部分患有哮喘的儿童可能对特定食物产生过敏反应，这种过敏反应可能加重哮喘症状，导致哮喘发作的频率和严重程度增加。常见的过敏原包括牛奶、鸡蛋、花生、坚果、海鲜、芝麻等。

（3）如何管理食物过敏

①识别过敏原：通过医疗检测，如皮肤点刺试验或血液检查，确定孩子对哪些食物过敏。

②避免接触过敏源：一旦确定过敏源，最重要的就是避免食用含有这些成分的食物。家长需要仔细阅读食物标签，了解食品中的成分。

③药物治疗：对于已经发生过敏反应的患者，可以使用抗组胺药、肾上腺素注射剂等药物来缓解症状。在严重过敏反应发生时，应立即使用肾上腺素，并就医处理。

④教育与应急预案：家长和孩子都应了解食物过敏的症状，并准备应急药物。学校和其他照看孩子的机构应知晓孩子的过敏情况，以防万一。

（4）食物过敏的预防

①早期引入：一些研究表明，适当的早期引入某些食物，如花生，可能有助于减少过敏的发生。不过，这一做法仍需在医生的指导下进行。

②母乳喂养：母乳喂养有助于增强婴儿免疫系统，有些研究认为它能降低食物过敏的风险。

**问题58什么是食物不耐受？**

食物不耐受是指在摄入某些食物后，人体出现的非免疫性反应。与食物过敏不同，食物不耐受通常不会引发严重的过敏反应（如过敏性休克）。食物不耐受的症状通常较为温和，但可能会持续较长时间，并且症状因人而异。

（1）食物不耐受的常见原因包括：

①消化系统问题：一些儿童可能由于消化酶不足（如乳糖不耐受）或肠胃功能问题，无法有效地分解某些食物成分，从而引发不适。

②化学成分过敏：某些食物中的添加剂、色素、调味料等化学成分可能引起过敏反应或不适感。

③食物中的天然化学物质：例如某些高氨基酸含量的食物（如巧克力、奶制品、酒精等）可能导致不耐受反应。

（2）食物不耐受的症状通常包括：

①消化系统症状：如腹痛、腹胀、恶心、呕吐或腹泻。

②皮肤症状：如湿疹或皮疹。

③呼吸系统症状：如喉咙不适或轻微的喘息，但不会引发急性过敏反应。

④头痛和偏头痛：一些食物成分可能引发头痛或偏头痛。

（3）儿童哮喘与食物不耐受的关系

对于哮喘儿童来说，食物不耐受可能会加重病情。虽然食物不耐受本身并不会直接引起哮喘发作，但一些研究表明，特定的食物可能会使已存在的哮喘症状更加严重，尤其是含有高盐分或防腐剂的加工食品。食物不耐受还可能与儿童的肠道健康密切相关，肠道功能不良可能导致免疫系统反应过度，从而增加过敏反应的风险。

（4）食物不耐受的诊断与管理

诊断食物不耐受通常通过排除法来进行。临床医生可能会建议家长记录孩子的饮食和症状，并在此基础上逐步去除可疑食物，观察是否能缓解症状。某些情况下，可能需要进行实验室测试或专门的食物挑战试验以确认具体的食物不耐受。

一旦确定了某种食物不耐受，管理的关键是避免摄入该食物，并注意提供均衡的饮食以确保营养的全面性。在哮喘管理中，合理控制食物不耐受可以帮助减少不必要的诱发因素，从而为孩子的健康提供更好的保障。

**问题59食物过敏的婴儿如何添加辅食？**

对于食物过敏的婴儿，辅食的添加需要格外谨慎，尤其是在哮喘或其他过敏性疾病的背景下。

（1）根据医生的建议制定计划：在为食物过敏的婴儿添加辅食之前，最好先与儿科医生或过敏专科医生讨论。这可以帮助确定哪些食物是安全的，哪些食物可能会引发过敏反应。

（2）选择低过敏性食物：对于食物过敏的婴儿，可以从低过敏性食物开始添加。以下是一些推荐的食物：

①米粉：米粉是最常见的初期辅食之一，通常不含常见过敏原（如牛奶、鸡蛋、坚果）。

②蔬菜泥：如胡萝卜泥、南瓜泥、土豆泥等，这些食物的过敏风险较低。

③水果泥：如苹果泥、梨泥等，建议选择较为温和且常见的水果。

（3）逐步引入新食物：为了观察是否有过敏反应，应采用“一次添加一种食物”的原则。每次引入新食物后，观察3到5天，看看婴儿是否出现皮肤红疹、呕吐、腹泻、呼吸困难等过敏反应。如果没有不适症状，可以逐步增加新的食物。

（4）避免常见过敏原：常见的食物过敏原包括：

①牛奶：许多婴儿对牛奶蛋白过敏。

②鸡蛋：鸡蛋也是一个常见的过敏源。

③坚果及花生：这些食物容易引发严重的过敏反应。

④海鲜及贝类：这些食物通常引发过敏性反应。 因此，在添加辅食时应避免这些潜在的过敏原，特别是在婴儿有食物过敏家族史时。

（5）密切观察过敏反应：在添加新食物时，需要密切观察婴儿是否出现以下过敏症状：

①皮肤反应：如湿疹、红疹或肿胀。

②消化系统症状：如呕吐、腹泻或腹痛。

③呼吸系统症状：如喘息、咳嗽或呼吸急促。

若婴儿出现过敏反应，应立即停止喂食该食物，并寻求医生的帮助。

（6）与过敏专家合作：如果婴儿有已知的食物过敏史，可能需要定期与过敏专家合作，进行皮肤点刺试验或血清免疫球蛋白E（IgE）检测，以便了解哪些食物可以安全食用，哪些需要避免。

（7）过敏性哮喘患者的辅食添加：对于有过敏性哮喘的婴儿，在添加辅食时要特别小心。某些食物可能加剧呼吸系统的反应，特别是过敏性哮喘的婴儿。如果有任何喘息加剧的现象，应立即停用该食物，并咨询医生。

**问题60如何管理食物过敏的儿童？**

（1）避免过敏源：家长需彻底了解孩子的过敏原，并严格避免接触相关食物。定期查看食物标签，确保食品不含过敏源成分。

（2）药物管理：家长应根据医生建议，备有抗组胺药、肾上腺素自动注射器等应急药物。特别是在外出时，携带这些药物是预防过敏反应的必要措施。

（3）教育与培训：家长、教师、以及孩子的其他照顾者需要接受过敏急救处理的培训，包括如何使用肾上腺素自动注射器。

（4）定期跟踪：定期到医院复查，尤其是儿童过敏反应的变化，需要与过敏科医生沟通。医生可能会建议定期进行皮肤点刺试验或血液检测，以监测过敏原的影响。

（5）饮食替代：在孩子的饮食中，如发现过敏源，需要提供替代食物，保证孩子的营养均衡。

管理食物过敏的最终目的是提高孩子的生活质量，同时通过严格的过敏源控制和及时的药物处理来避免严重过敏反应。通过教育和准备，家长能够为孩子创建一个更安全的生活环境。

**问题61如何诊断食物过敏？**

（1）临床症状和病史评估

诊断食物过敏的首要步骤是通过详细的病史收集和症状评估。家长或患者应向医生提供以下信息：

①症状出现的时间和情况：是否在食用特定食物后出现反应，例如皮肤荨麻疹、喉咙肿胀、呼吸困难、呕吐或腹泻等。

②家族史：过敏性疾病（如哮喘、湿疹、过敏性鼻炎等）的家族历史可以增加食物过敏的风险。

（2）皮肤点刺试验（Skin Prick Test，SPT）：皮肤点刺试验是一种常用的食物过敏检测方法。通过在患者皮肤上轻轻刺入微量的食物提取液，观察是否产生过敏反应（如局部红肿）。该方法通常用于诊断常见的食物过敏原，如牛奶、鸡蛋、坚果等。

（3）血清特异性IgE抗体检测：血液检查可以测量特定食物过敏原的IgE抗体水平。血清中IgE抗体水平升高可能表明患者对特定食物有过敏反应。此方法适用于无法进行皮肤试验的患者，或者当皮肤试验结果不明确时。

（4）食物激发试验：在某些情况下，当皮肤试验和血液检查结果不确定时，医生可能建议进行食物激发试验。这是通过让患者在严格监控下逐步食用疑似过敏的食物，观察是否引发过敏反应。此方法仅在医院环境中进行，并由专业医生操作，以保证安全。

（5）排除诊断：如果患者有明显的食物过敏症状，但上述检测结果未能确定过敏源，医生可能会建议暂时排除疑似食物，观察症状是否缓解。这一方法有助于进一步确定过敏源。

（6）临床观察和长期管理：对于已确诊的食物过敏患者，长期的过敏管理至关重要。患者和家属需要与医生密切合作，制定避免过敏源的饮食计划，并了解如何应对过敏反应，包括急救药物的使用（如肾上腺素注射器）。

**问题62哮喘患儿能注射疫苗吗？**

是的，哮喘患儿一般可以且应该接种大多数疫苗，除非存在特定禁忌症。疫苗接种对于哮喘患儿尤其重要，因为某些呼吸道感染会增加他们的哮喘发作风险。然而，接种疫苗时需要考虑患儿的哮喘病情、疫苗种类以及当前哮喘控制状况。

以下是一些关键的指导原则：

（1）灭活疫苗：灭活疫苗（如流感疫苗、肺炎疫苗、百白破疫苗等）一般对哮喘患儿是安全的。建议哮喘患儿按时接种流感疫苗，因为流感感染可能引发哮喘急性发作。

（2）减毒活疫苗：减毒活疫苗（如麻疹、腮腺炎、风疹联合疫苗MMR、水痘疫苗等）通常也适合哮喘患儿，但需评估其病情。哮喘患儿如果正在接受全身性免疫抑制治疗或哮喘未得到有效控制时，应谨慎接种，并咨询医生的建议。

（3）哮喘急性发作期：在哮喘急性发作期间或哮喘症状控制不佳的情况下，建议推迟疫苗接种，待哮喘得到有效控制后再接种，以免疫苗接种引起哮喘症状加重。

（4）过敏史：如果患儿对疫苗成分有过敏史，特别是鸡蛋（例如某些流感疫苗可能含有鸡蛋成分），需要在接种前与医生讨论，评估风险。

（5）定期随访与个体化评估：每个哮喘患儿的病情不同，因此建议家长与儿科医生或哮喘专科医生进行个体化评估，确保安全接种。

注意事项

（1）哮喘患儿在接种疫苗后应监测哮喘症状，有时可能会出现轻微的呼吸道症状，但通常是短暂的。

（2）接种疫苗前，如果患儿使用过吸入性糖皮质激素或短期口服类固醇，需要根据医生的建议调整或观察。

**问题63哮喘发作与上呼吸道感染有什么关系？**

哮喘发作与上呼吸道感染（URTI）之间存在密切关系。上呼吸道感染，尤其是由病毒引起的感染，如鼻病毒、流感病毒或呼吸道合胞病毒等，是儿童哮喘发作的主要诱因之一。其机制可以通过以下几个方面来解释：

（1）炎症反应加剧：上呼吸道感染会引发呼吸道局部的炎症反应，加重气道炎症和敏感性，使得气道更易收缩和反应过度。这种加剧的炎症反应是哮喘发作的主要原因之一。

（2）气道高反应性增加：病毒感染不仅会直接引发炎症，还会导致气道上皮细胞的破坏，增加气道对过敏原和其他环境因素的敏感性。这种高反应性使得哮喘儿童在暴露于病毒感染后更易出现气道收缩和喘息症状。

（3）免疫系统影响：上呼吸道感染期间，人体的免疫系统被激活，释放出各种细胞因子和化学信号物质。这些信号物质可能导致气道平滑肌的收缩和粘液分泌增加，加重气道阻塞。

（4）触发过敏性反应：在一些哮喘患儿中，病毒感染可以触发过敏性免疫反应，产生更多的IgE抗体，从而进一步加重气道的过敏反应和哮喘症状。

（5）影响吸入治疗的效果：感染期间，气道充血和分泌物增多可能影响药物的吸收，使得常规的哮喘控制药物（如吸入性糖皮质激素）疗效降低。因此，在上呼吸道感染期间，可能需要调整哮喘管理方案，以更好地控制症状。

管理建议

对于儿童哮喘患者来说，预防上呼吸道感染对哮喘控制至关重要。家长可以采取以下措施：

（1）减少接触传染源：避免与感冒或其他呼吸道感染者密切接触，尤其是在病毒传播高峰期。

（2）加强免疫系统：确保儿童按时接种流感疫苗等预防性疫苗，有助于减少相关病毒感染的风险。

（3）保持良好卫生习惯：勤洗手、避免用手接触口鼻，减少感染机会。

（4）及时就医：当儿童出现感冒症状且哮喘病史时，应及时咨询医生，以避免感染诱发哮喘急性发作。

**问题64哮喘患儿可以运动吗？如何运动？**

哮喘患儿在病情得到有效控制的情况下是可以运动的。适当的运动不仅可以增强患儿的体质，还能帮助改善呼吸功能和心肺耐力，促进身心健康。然而，家长和临床医师需要共同关注运动的选择和安全性，以避免诱发哮喘症状。以下是一些适合哮喘患儿的运动建议：

（1）选择适合的运动项目

①低强度运动：适合哮喘患儿的低强度运动包括散步、游泳、骑自行车等。游泳是较推荐的运动方式，因为湿润的空气有助于减少气道刺激，并且水的温度对呼吸道刺激较小。

②间歇性运动：例如羽毛球、乒乓球等，这类运动可以在中间休息，减少持续的高强度运动对呼吸道的压力。

③户外运动：可以选择空气质量较好的时候进行户外活动。若空气污染较严重或气温较低时，建议减少或避免户外运动，以防气道刺激。

（2）运动前的预防措施

①充分热身：哮喘患儿在运动前应进行10-15分钟的热身，逐渐增加活动强度，帮助呼吸系统适应运动需求。

②使用哮喘药物：在运动前，医生可能会建议患儿使用吸入型支气管扩张剂（例如沙丁胺醇）来预防运动诱发的哮喘发作，但具体药物和使用量需遵循医嘱。

③避免诱发因素：运动环境应尽量避免接触过敏原（如花粉、尘螨等）和刺激物（如烟雾、空气污染等），以减少哮喘发作风险。

（3）运动中注意事项

①监测症状：家长或陪同人员应随时观察患儿的状态，留意是否出现喘息、呼吸急促、胸闷等哮喘症状。

②适时休息：如果患儿感到不适，应立即停止运动并休息。轻微哮喘症状时可以通过呼吸调整来缓解，但若症状严重则需及时使用急救药物。

③保持稳定节奏：避免过度激烈的运动，保持适中的运动强度。可以通过调整呼吸频率和节奏来避免气道的过度刺激。

（4）运动后的冷身

逐步降温：运动结束后应进行5-10分钟的放松和冷身，逐步减少心率和呼吸频率，避免突然停止运动引发哮喘症状。

（5）与医师保持沟通

①定期检查：患儿应定期进行哮喘控制评估，医生会根据病情调整运动方案。

②运动计划调整：若哮喘症状有所变化，及时与医生沟通，可能需要调整运动项目和强度。

**问题65运动能诱发哮喘发作吗？**

运动确实可能诱发哮喘发作，这种情况称为运动诱发性哮喘（EIA，exercise-induced asthma）或运动诱发支气管痉挛（EIB，exercise-induced bronchoconstriction）。在儿童哮喘患者中，运动后出现气道收缩、喘息、呼吸急促等症状较为常见。这些症状通常在运动开始后的几分钟内逐渐出现，并在运动停止后持续一段时间。

（1）运动诱发哮喘的原因：运动导致的哮喘症状主要由于快速呼吸时气道暴露于冷、干燥的空气，从而引发气道炎症和收缩。运动时身体需要更多的氧气，呼吸变得更深更快，使得通过鼻腔温暖、湿润空气的机会减少。对于哮喘患者，尤其是儿童，这种情况更容易诱发气道过敏反应。

（2）常见的诱发因素

①运动种类：如长时间的跑步、游泳等耐力运动更容易诱发哮喘症状，而短时间的低强度运动相对风险较低。

②环境因素：干燥、寒冷的天气，或污染较重的环境更易加重症状。

③呼吸方式：口呼吸会使空气直接进入下呼吸道，缺乏鼻腔加温加湿的效果。

（3）预防与管理：为了帮助儿童哮喘患者安全进行运动并减少症状发作的风险，可以采取以下预防和管理措施：

①预防用药：在运动前15-30分钟使用短效支气管扩张剂（如沙丁胺醇），能有效预防症状发作。

②逐步热身：运动前的热身可以让身体适应运动带来的负荷，减少运动开始时的气道刺激。

③选择合适的运动：推荐相对温和的运动，如游泳、步行，或在适宜温湿度的环境中进行运动。

④控制环境：避免在干燥、寒冷或空气污染严重的地方运动，可以选择在室内进行适当运动。

（4）家长的支持与关注

家长可以通过以下方式帮助儿童管理运动诱发性哮喘：

①观察并记录症状：了解并记录儿童在不同运动中的症状表现，帮助医生更好地了解孩子的状况。

②及时用药：确保孩子在运动前按医嘱使用预防药物，掌握吸入器的正确使用方法。

③鼓励适量运动：运动对儿童的体力和免疫系统健康至关重要，应尽量鼓励适当运动，以增强肺功能和总体健康。

运动诱发性哮喘并不意味着孩子完全不能运动。合理管理和适量运动不仅能帮助控制哮喘，还对孩子的身心健康有益。然而，如果孩子在运动后症状反复严重或持续加重，应及时就医以调整治疗方案。

**问题66如何预防运动诱发的哮喘发作？**

（1）合理使用药物

①预防性药物：在运动前，建议根据医生指导使用短效β2受体激动剂（如沙丁胺醇）作为预防措施，通常在运动前10-15分钟使用。对于长期控制哮喘的患者，医生可能会建议定期使用吸入性糖皮质激素等长期控制药物。

②调整药物治疗计划：如果运动诱发哮喘频繁发作，可能需要重新评估哮喘的控制水平，并调整长期的药物治疗计划。

（2）选择适合的运动类型

①低风险运动：一些运动（如游泳、步行、骑自行车）对哮喘患者较为友好，因为这些运动通常不会诱发高强度的呼吸需求。

②避免剧烈运动：避免高强度、持久的运动（如长时间的跑步、足球等），尤其是在冷空气或干燥的环境中，因为这些运动可能会加重气道的反应性。

（3）热身和冷身

①充分热身：运动前的充分热身有助于让气道适应运动所需的呼吸量，减少哮喘发作的风险。建议在运动前进行10-15分钟的低强度热身，如轻微的拉伸、慢跑等。

②逐渐冷身：运动后逐渐冷身可以避免突然的呼吸变化，从而减少诱发哮喘的可能性。

（4）注意环境因素

①避免冷空气和干燥环境：寒冷和干燥的空气容易刺激气道，诱发哮喘发作。运动时可以佩戴围巾，或者在温暖湿润的环境下进行活动。

②避免空气污染：户外空气污染也会加剧哮喘的风险。尽量选择空气质量良好的时间段和地点运动。

（5）教育与沟通

①教育儿童和家属：了解哮喘症状，帮助儿童认识和管理自己的哮喘症状。家属和教练员要了解急性发作的处理方法，以便在紧急情况时及时应对。

②沟通并制定应急方案：在学校或运动场所，通知教练或指导老师哮喘的情况，并制定应急方案。确保随时携带哮喘药物，必要时可立即使用。

（6）定期复查：儿童的身体在不断发育，哮喘症状和药物需求可能会发生变化。定期复查可以帮助医生及时调整治疗方案，保障运动安全。

**问题67情绪波动能诱发哮喘发作吗？如何预防？**

情绪波动确实可以诱发哮喘发作。情绪紧张、焦虑、愤怒等负面情绪会引发身体的应激反应，导致交感神经系统兴奋，释放大量应激激素（如肾上腺素和皮质醇）。这类反应会引起气道收缩、呼吸加快、心率增加，从而加重哮喘症状，甚至导致哮喘急性发作。特别是在儿童中，由于他们的情绪调节能力较弱，情绪波动更易对哮喘病情产生影响。

（1）预防措施

①情绪管理训练：帮助孩子学会识别、表达和调节情绪，例如通过深呼吸、冥想或想象放松法等方法，帮助他们平复心情，降低情绪对身体的刺激。

②家庭心理支持：家庭成员应积极关心儿童的情绪变化，保持耐心并给予足够的支持，避免在生活中出现过多的压力和紧张情绪，营造一个平和、愉快的家庭环境。

③认知行为训练：通过认知行为疗法（CBT），帮助儿童理解情绪与哮喘的关系，逐步学习如何控制自己的情绪反应。这种方法可以有效减少负面情绪对哮喘的诱发作用。

④适度运动：规律的轻度到中度运动有助于提高心肺功能，增强身体对情绪波动的适应力。但需要在医生指导下选择合适的运动形式和强度，以免运动诱发哮喘发作。

⑤保持日常规律：良好的作息习惯和规律的生活能有效降低情绪波动的频率，减少对哮喘的负面影响，保证睡眠充足、饮食健康，避免因疲劳引发的情绪不稳。

（2）药物支持

对于部分容易因情绪波动诱发哮喘的儿童，医生可能会建议适量使用短效支气管扩张剂（如沙丁胺醇）作为预防手段。在情绪波动较大或即将面临可能导致紧张情绪的情境前，可以在医生指导下提前使用，但必须避免依赖。

**问题68哮喘对患儿及家长会产生哪些心理行为影响，如何干预？**

哮喘对患儿及其家长产生的心理行为影响较为广泛，常表现为焦虑、抑郁和心理负担的增加，且可能影响家庭的整体生活质量。

1. 对患儿的影响

（1）焦虑和恐惧：哮喘发作的突然性和呼吸困难会让孩子产生焦虑感，害怕再次发作，甚至可能产生对运动或外出活动的畏惧。

（2）自信心受挫：与健康儿童相比，哮喘患儿可能需要在生活和活动中有所限制，容易因此产生自卑心理，影响其自信心和社交互动。

（3）学业和情绪困扰：反复发作的症状和频繁的医疗干预可能影响孩子的出勤率和学习成绩，加之患病带来的情绪波动，可能导致患儿的注意力和情绪控制力下降。

2. 对家长的影响

（1）焦虑和无助感：家长可能因为担心孩子的健康而产生焦虑，特别是在哮喘发作时，容易感到无助，担心无法及时帮助孩子。

（2）家庭生活的压力增加：哮喘儿童需要长期管理和护理，可能会让家长感到身心疲惫，甚至可能产生挫败感，影响日常生活和家庭和谐。

（3）过度保护倾向：有些家长因为对哮喘发作的恐惧，可能对孩子采取过度保护的措施，反而限制了孩子的正常活动空间，影响其心理健康和自我管理能力的发展。

3.干预措施

为了缓解哮喘对患儿及家长的心理行为影响，可以通过以下几种方式进行干预：

（1）心理支持与教育

①心理咨询：为患儿提供心理支持和咨询，帮助他们应对焦虑和恐惧。心理咨询可以帮助孩子学会识别和调节负面情绪，增强自我控制感。

②家长教育：通过哮喘管理课程，帮助家长了解哮喘的发作和控制方式，减轻家长的焦虑。同时，还可以学习如何支持孩子的情绪和独立性，避免过度保护。

（2）哮喘管理知识培训

①药物管理培训：帮助患儿及家长掌握正确的吸入技术和药物使用方式，增强对哮喘管理的信心。

②发作预警系统：教育患儿及家长识别哮喘发作的早期症状，采取有效的应对措施，减少急性发作带来的心理负担。

③生活方式指导：包括体育活动的安全建议和饮食管理，帮助患儿维持积极的生活方式。

（3）同伴支持和团体活动

①哮喘支持小组：为患儿和家长提供与其他哮喘患儿家庭交流的机会，共享管理经验和情感支持，有助于减轻孤独感和无助感。

②团体活动和健康教育讲座：鼓励患儿与其他同龄人互动，通过适当的活动提高他们的自信心和社交能力，同时让家长也能在支持团体中获得理解和安慰。

（4）学校和社会支持

①学校支持：与学校沟通，确保患儿在学校能获得适当的支持，比如允许必要的药物携带和发作时的紧急处理措施。

②社会资源的利用：利用社区和社会资源，如哮喘协会和健康组织的教育和支持服务，帮助家庭获得更全面的支持和资源。

**问题69空气污染能诱发哮喘发作吗？如何防护？**

空气污染确实可以诱发哮喘发作。空气中的污染物，如颗粒物（PM2.5、PM10）、臭氧、氮氧化物和挥发性有机化合物等，会刺激呼吸道，导致气道炎症和收缩，从而加重哮喘症状。对儿童哮喘患者而言，空气污染可能会增加气喘、呼吸急促、咳嗽等症状的发生频率和严重程度，甚至引发急性哮喘发作。因此，采取有效的防护措施是帮助减少空气污染对儿童哮喘患者影响的关键。

以下是一些防护措施：

（1）减少户外活动：在空气污染指数较高的日子，尽量减少儿童的户外活动，尤其是剧烈运动。空气质量监测数据可以帮助判断空气状况，通常建议在空气质量指数（AQI）高于100时避免外出。

（2）使用空气净化设备：在室内使用空气净化器，尤其是在睡眠环境中，可以有效减少室内空气中的污染物浓度，提供一个较为清洁的呼吸环境。

（3）关闭门窗：空气污染严重时，建议关闭门窗，减少污染物进入室内的机会。但要注意保持适当的通风，防止室内空气变得过于密闭。

（4）使用口罩：在必须外出的情况下，为孩子佩戴适合的防护口罩（如N95口罩）可以减少吸入空气中的颗粒污染物。然而，需确保儿童佩戴的口罩适合其面部大小且舒适，避免引起呼吸困难。

（5）定期关注空气质量预报：家长可以通过空气质量监测应用或网站获取最新的空气污染指数和预报，提前了解空气状况，以便调整孩子的活动计划。

（6）避免高污染环境：如尽量避免在交通繁忙的道路附近散步或在高峰时间出行，减少暴露在车辆尾气中的机会。

（7）咨询医生：若空气污染严重并影响到哮喘症状，及时与医生沟通，可能需要调整哮喘控制药物或增加急救药物，以预防急性发作。

（8）加强免疫力：家长可通过合理的膳食、充足的睡眠和适当的运动，帮助孩子增强免疫力，从而更好地抵抗外界不良环境对呼吸系统的刺激。

**问题70哪些刺激性气味容易诱发哮喘发作？如何预防？**

1. 常见的诱发气味

（1）烟雾：香烟、雪茄或其他烟草制品产生的烟雾。二手烟和三手烟（残留在衣物、墙壁和家具表面的烟味）同样会对儿童造成危害。

（2）香水和空气清新剂：一些香水、空气清新剂和除臭剂中含有挥发性化学成分，可能会刺激呼吸道，引发哮喘。

（3）清洁剂和化学品：漂白剂、消毒剂和其他强效清洁产品通常含有强烈的化学气味，对呼吸道刺激较大。

（4）烹饪烟雾：烹饪时产生的烟雾、油烟，尤其是在使用高温煎炸时，可能含有颗粒物质，对哮喘患者造成刺激。

（5）油漆和溶剂：油漆、胶水、稀释剂等常用溶剂含有挥发性有机化合物（VOCs），会引起呼吸系统反应。

（6）花粉和植物：某些植物花粉较浓，释放出的气味和花粉可能诱发哮喘症状。

2. 预防措施

（1）保持室内空气质量：确保家中良好的通风，特别是在烹饪和使用清洁剂时，尽可能打开窗户或使用排气扇。

（2）避免二手烟暴露：家庭成员或访客应避免在室内吸烟，尽量保持居住环境的无烟状态，儿童接触过的衣物和环境也要避免烟草残留。

（3）慎用香水和化学产品：尽量减少香水、空气清新剂等刺激性产品的使用，选择无香型、低挥发的清洁产品。

（4）选择温和的清洁剂：避免使用含氯和氨等强刺激成分的清洁剂，改用温和的清洁产品或自然替代品（如醋、苏打等）。

（5）减少花粉暴露：在花粉浓度较高的季节，关闭门窗，外出时佩戴口罩。使用空气净化器来减少室内花粉。

（6）及时清洁和更换空气过滤设备：空调、空气净化器等设备需要定期更换滤芯，保持设备清洁以减少空气中的刺激物。

**问题71哪些气候变化容易诱发哮喘发作？如何预防**？

气候变化在诱发儿童哮喘方面起着重要作用，特别是在季节交替、温差显著以及空气湿度变化显著的情况下，更易导致哮喘发作。以下是几种主要的气候变化及其对哮喘的影响：

（1）季节变化和温度波动：春季和秋季的温差较大，花粉浓度和空气中的过敏原显著增加。这会引起哮喘患者的气道敏感性上升，从而增加哮喘发作的可能性。

（2）湿度的突然变化：湿度过高（如在夏季或雨季）或过低（如冬季或干旱季节）都会对气道产生不利影响。高湿度会使霉菌和尘螨繁殖，低湿度则使空气干燥，从而刺激气道，引发哮喘症状。

（3）空气污染增加：气候变化可能增加空气污染，特别是在天气闷热时，空气中污染物（如颗粒物、臭氧和一氧化碳）浓度较高。空气污染与哮喘发作密切相关，尤其是对于儿童。

（4）冷空气刺激：冬季的冷空气会使气道收缩，对气道较为敏感的哮喘患者来说，容易诱发哮喘。

预防措施

（1）关注天气变化：家长应关注天气预报，特别是在温差较大的季节或湿度变化显著的情况下，尽量减少孩子在户外活动的时间。

（2）定期清洁室内环境：保持室内清洁，尤其是减少尘螨、霉菌等过敏原的存在。使用空气净化器和除湿机有助于维持适宜的室内空气质量。

（3）避免在高污染天气外出：雾霾或空气质量差的天气时，应尽量减少孩子户外活动，并使用空气净化器保证室内空气质量。

（4）防护措施：在寒冷天气时，出门时为孩子戴上口罩和围巾，减少冷空气直接进入气道的机会，从而降低哮喘发作的风险。

（5）药物管理：对于已经确诊哮喘的儿童，家长应在医生指导下做好日常药物的使用，包括预防性药物和缓解药物。在气候变化剧烈的时期，确保随身携带急救药物，以备突发哮喘使用。

**问题72香烟暴露对哮喘患儿有什么不良影响？如何预防？**

香烟暴露对哮喘患儿有显著的不良影响。儿童接触香烟烟雾（被动吸烟）或吸入二手烟，可导致哮喘症状加重、急性发作频率增加，并可能引发长期的肺部损伤。具体而言，香烟暴露会使患儿的气道变得更敏感，容易受到刺激，引发呼吸困难、喘息和咳嗽等症状。此外，长期的香烟暴露还可能导致肺功能的下降，影响孩子的正常呼吸发育，增加哮喘的严重性和对药物的依赖。

1.香烟暴露的主要不良影响包括：

（1）增加哮喘发作频率：香烟中的有害物质会加重气道的炎症，导致哮喘患儿更频繁地出现发作。

（2）导致症状加重：香烟烟雾中的化学物质会引起气道黏膜的刺激，使喘息、咳嗽和呼吸困难等症状加剧。

（3）影响长期肺功能：研究表明，长期接触香烟烟雾会抑制肺部的正常发育，导致肺功能下降，并可能在成年后产生持久的呼吸系统问题。

（4）增加药物需求：哮喘患儿暴露于香烟烟雾中，可能需要更高剂量的哮喘药物进行控制，增加了副作用的风险。

2.预防措施

（1）家庭内完全禁烟：家长及其他家庭成员应避免在家中、汽车内或儿童活动的任何封闭环境中吸烟，以减少二手烟暴露。家长应意识到，即使在房间内不吸烟，但在家中或车内残留的烟雾成分也可能对孩子有害。

（2）外出时避免接触香烟烟雾：尽量避免带孩子前往允许吸烟的公共场所。如果在户外遇到吸烟者，应尽快远离或带孩子到空气清新的区域。

（3）教育家庭成员与亲友：告知所有与孩子有密切接触的亲友和访客香烟烟雾对哮喘儿童的影响，避免在孩子附近吸烟。可以提供替代的户外吸烟区，帮助减少孩子的二手烟暴露。

（4）促进无烟政策：鼓励学校、社区中心、儿童活动场所等推广无烟政策，营造无烟环境。

（5）引导戒烟：如果家庭成员有吸烟习惯，建议进行戒烟以减少家庭内的二手烟暴露。可以咨询医生或使用戒烟辅助工具，减少对孩子的健康影响。

**问题73哮喘与肥胖有关吗？如何预防？**

1.哮喘与肥胖的关系

临床研究发现，哮喘与肥胖之间存在一定的关联。肥胖增加了哮喘的患病风险，尤其是在儿童中。肥胖可能加重哮喘的症状，增加呼吸困难的概率，还可能导致哮喘的治疗效果下降。肥胖会使体内的慢性炎症水平升高，进而影响肺部功能，并可能促使哮喘症状的发生或加重。此外，肥胖儿童的气道反应性可能更高，导致气道更易出现过敏性反应，加剧哮喘的风险。

2.预防措施

（1）健康饮食：为孩子提供营养均衡的饮食，避免高糖、高脂肪的食物。增加水果、蔬菜、全谷物和优质蛋白质的摄入，以帮助维持健康的体重并降低体内炎症水平。

（2）保持日常运动：定期运动不仅有助于控制体重，还可以改善心肺功能，增强免疫力。对于哮喘儿童，建议适当选择运动强度较低且能逐步提高耐力的活动，如游泳、步行等。应避免剧烈运动，以免诱发哮喘症状。

（3）控制体重：健康的体重管理对于降低哮喘风险非常重要。通过合理的饮食和适当的运动，帮助孩子保持在合适的体重范围内。如果儿童的体重明显超标，可咨询营养师或医生以制定合适的减重计划。

（4）避免过敏原和诱发因素：哮喘患者应尽量避免接触过敏原，如灰尘、花粉、宠物毛发等。此外，避免烟雾、空气污染等刺激性气体对孩子呼吸道的伤害，可以减轻哮喘症状的频率。

（5）定期监测和随访：对于哮喘风险较高或已有哮喘症状的儿童，建议定期进行健康检查，监测其肺功能、体重指数等相关指标。通过与医生保持沟通，及时调整健康管理方案。

（6）心理健康支持：肥胖与哮喘的发生可能会给儿童带来心理压力，增加焦虑或自卑感。家长应关注孩子的心理健康，提供积极的情感支持，必要时可寻求心理咨询的帮助。

**问题74外出活动或旅游时，有哪些因素容易导致哮喘急性发作？**

（1）环境过敏原：不同的地区有不同的过敏原。例如，花粉、尘螨、霉菌、动物皮屑等。季节性花粉尤其在春季和秋季常见，因此如果去往花粉含量高的地区，患儿可能会接触到新的过敏原而导致哮喘发作。

（2）空气污染：城市的空气污染和乡村的烟尘污染都是常见的诱发因素。尤其在空气质量不佳（如PM2.5和PM10较高）的环境中，吸入污染物会刺激患儿的呼吸道，加重哮喘症状。

（3）气候和温度变化：突然的温度变化，尤其是冷空气，可能会引起气道收缩，诱发哮喘。在潮湿或湿度极高的环境中，霉菌孢子数量增加也会引发哮喘反应。

（4）活动量增加：户外活动和运动增加了身体对氧气的需求，尤其是在寒冷空气中跑跳，可能会诱发哮喘症状。过度的体力活动可能导致患儿呼吸急促，从而刺激气道。

（5）旅途中的紧张与压力：旅行的紧张、兴奋、焦虑等情绪可能也会诱发哮喘，尤其是对于敏感的患儿。身体处于应激状态时，可能会增加气道的敏感性。

（6）食物或药物过敏：在旅行中接触到新的食物或药物时，可能会发生过敏反应，进而诱发哮喘。如果患儿有明确的食物或药物过敏史，旅行时应特别注意，避免误食或误用药物。

（7）空气干燥：特别是在冬季或空调房间，空气干燥可能会导致气道干燥，诱发哮喘。因此在环境干燥的情况下，保持适当的空气湿度可能有助于预防哮喘发作。

预防建议：

（1）在外出前，了解目的地的过敏原及空气质量，做好防护措施。

（2）随身携带吸入剂和其他必要的急救药物，并与医生讨论应急管理策略。

（3）避免在高污染和温度极端变化的环境中进行剧烈活动。

（4）注意患儿的情绪和饮食，减少应激反应。

**问题75哮喘急性发作或加重有时间特点吗？**

（1）夜间和凌晨：哮喘的急性发作往往在夜间或凌晨加重。这一时间段的哮喘发作可能与多种因素有关，包括体内激素的昼夜波动（如皮质醇和肾上腺素的水平变化），这些激素的浓度在夜间通常较低，而炎症因子如白细胞介素-5的水平会相对较高，从而可能导致气道更加敏感、气道收缩增加。这种生理变化可能使夜间和清晨成为哮喘加重的高峰期。

（2）季节性：哮喘急性发作的时间特点还会受到季节变化的影响。例如，在花粉含量较高的春季和秋季，部分患者会因过敏原（如花粉、霉菌孢子等）增加而导致哮喘发作频率上升。对于有季节性过敏性哮喘的儿童患者，季节性变化可能会成为发作的触发因素。

（3）空气污染或气候变化：空气污染水平较高的日子，如雾霾天，或温度和湿度急剧变化时，也可能引发或加重哮喘症状。此外，在天气突然变冷的秋冬季，冷空气可刺激气道，诱发哮喘急性发作。因此，哮喘患者应尽量减少在空气污染较重的时段外出，或在出门时佩戴口罩。

（4）日常活动时间：哮喘急性发作的时间特点还会受到患者日常活动的影响，例如运动后或体力活动较大时，尤其是剧烈运动或户外活动后容易诱发哮喘急性发作。这一时间点与患者的个体差异相关，运动诱发型哮喘在儿童哮喘患者中较为常见。

**问题76哪些表现可以预示儿童哮喘的急性发作？**

（1）呼吸急促和困难：儿童在平静状态下出现呼吸急促或明显呼吸困难，尤其是在夜间或清晨时更为明显。家属应注意孩子是否在呼吸时费力，是否表现出呼吸短促的迹象。

（2）持续性或加重的咳嗽：咳嗽是哮喘的常见症状，尤其是干咳。如果儿童出现持续性咳嗽，特别是在夜间、清晨或运动后更为显著，可能提示哮喘的急性加重。

（3）胸闷或胸痛：儿童可能会感到胸部不适，包括胸闷和偶尔的胸痛，家属可以通过询问了解他们的感觉。如果孩子抱怨“喘不上气”或“胸口压迫感”，这是一个重要的警示信号。

（4）喘息音（哮鸣音）：在哮喘急性发作前，家属可能会听到孩子呼吸时出现的哮鸣音，尤其是在呼气时更为明显。这个高音调的哨声是气道收缩的标志，是哮喘急性发作的典型表现。

（5）身体疲倦或食欲不振：因为哮喘急性发作时会导致身体缺氧，儿童可能会表现出极度的疲倦感，或出现食欲下降的情况，这可能也是哮喘恶化的征兆。

（6）日常活动受限：如果孩子因呼吸不畅而避免跑跳、游戏等活动，这也可能是哮喘症状加重的信号。家属应注意观察孩子活动量的变化，尤其是在运动后有喘息、咳嗽或疲劳加重的情况。

（7）情绪变化：有些儿童在哮喘急性发作前可能出现焦虑、易怒等情绪变化，这是由于缺氧或身体不适导致的情绪波动。家属应留意孩子的情绪变化，尤其是突然变得易怒或安静。

以上表现并不意味着每个哮喘急性发作的儿童都会全部出现，但这些症状的组合或单一症状的显著加重可能提示急性发作的来临。家属和临床医生应特别关注这些变化，一旦确认急性发作迹象，应立即采取措施，如使用紧急哮喘吸入剂或咨询专业医生。

**问题77如何判断儿童哮喘急性发作的严重程度？**

（1）呼吸频率与节律

①轻度发作：呼吸稍微增快，但不会出现明显的呼吸困难或喘息的节律异常。

②中度发作：呼吸明显增快，孩子可能有轻度的呼吸困难，讲话时可能断断续续，呼气时间延长，使用鼻翼或辅助肌肉呼吸。

③重度发作：呼吸急促且不规则，可能会看到孩子使用呼吸辅助肌肉（如颈部和胸部肌肉），呼吸急促且困难，讲话或哭闹时明显不连贯，甚至不能正常进行日常活动。

（2）哮鸣音（喘息声音）

①轻度发作：喘息声音不明显或仅在呼气时出现。

②中度发作：喘息声音较明显，可能伴随吸气和呼气时的声音。

③重度发作：喘息声音非常明显，甚至在静止状态下也能听到，或者喘息声音逐渐减弱（可能是因为气道严重阻塞，空气流动减少），需特别注意此情况。

（3）呼吸辅助肌肉的使用情况

①轻度发作：不需要使用辅助呼吸肌肉。

②中度发作：会使用鼻翼、胸肌或腹肌来辅助呼吸，表现为“吸气时胸廓内陷”。

③重度发作：显著使用辅助肌肉，如颈部、胸部及肩膀肌肉等，且呼吸节律不均匀。

（4）氧饱和度

①轻度发作：氧饱和度（SpO₂）通常在95%以上。

②中度发作：氧饱和度在92%到95%之间。

③重度发作：氧饱和度低于92%，此时应尽快就医。

（5）其他症状

①轻度发作：轻度咳嗽、轻微不适，日常活动不受明显影响。

②中度发作：持续咳嗽、胸闷、活动受限，可能出现焦虑或不适感。

③重度发作：严重胸闷、无法正常说话或哭泣，意识模糊、疲劳，甚至可能出现青紫（唇部或指甲发紫）。

（6）哮喘控制测试和峰流速计（如果有）

①在有条件的情况下，可以使用哮喘控制测试（如ACT评分）或峰流速计来评估孩子的肺功能状态。

②轻度发作：峰流速下降不超过个人最佳值的20%。

③中度发作：峰流速下降20%到50%。

④重度发作：峰流速下降超过50%。

紧急情况处理

（1）轻度发作：可以使用快速缓解药物（如短效β2受体激动剂）来缓解症状，观察孩子是否有改善。

（2）中度或重度发作：应立即使用缓解药物，并尽快就医，以便医生提供更强效的药物和支持性治疗。

（3）极重度发作（如出现呼吸停止、意识丧失等）：立即拨打急救电话，尽早进行急救处理。

**问题78因急性发作住院治疗的哮喘患儿，其出院后的注意事项有哪些？**

（1）遵医嘱用药

①出院时，医生会开具必要的药物，如吸入性糖皮质激素、支气管扩张剂等。这些药物的剂量和使用频率需严格遵循医生的指导，尤其是维持用药的吸入剂，应按时按量使用，防止哮喘症状复发。

②注意随身携带应急药物（如短效β2受体激动剂），以应对突发症状。同时，教会患儿家属和患儿正确的药物使用方法，确保吸入装置使用得当。

（2）定期随访

①出院后建议定期复诊，通常在1-2周内安排初次复查，以便医生评估患儿病情的恢复情况，并调整治疗方案。之后，可根据病情需要每3-6个月复查一次。

②在复诊中，医生可能会进行肺功能测试和症状评估，以判断治疗效果及哮喘控制情况，必要时调整药物。

（3）监测症状和触发因素

①家属应密切观察患儿的呼吸状况，如是否有咳嗽、气喘、胸闷等症状，及时记录以便医生参考。

②注意避免可能的过敏原和刺激物，例如花粉、灰尘、二手烟、冷空气和动物毛发等。同时，保持居住环境清洁，避免潮湿和霉菌滋生。

（4）培养良好的生活习惯

①饮食健康：平衡饮食，避免过多刺激性或易引发过敏的食物，例如海鲜、坚果等。

②规律作息：保证充足的睡眠，避免过度劳累，因为疲劳可能会加重哮喘症状。

③适当锻炼：在医生的建议下，安排适度的运动，增强免疫力，但要避免剧烈运动，以防诱发哮喘。

（5）家庭健康教育

①教育家属和患儿了解哮喘的基础知识，包括哮喘的触发因素、如何使用吸入药物以及在紧急情况下的处理方法。

②制定家庭应急预案，确保在哮喘发作时家属能够迅速采取措施，帮助患儿稳定症状，并及时就医。

（6）注意季节变化

①在寒冷或过敏高发季节，特别注意防寒保暖，预防上呼吸道感染，因为感冒等感染容易引发哮喘急性发作。

②可根据医生建议提前进行抗过敏治疗，帮助降低过敏性哮喘的发作风险。

**问题79如何判断与识别处于哮喘行动计划绿区的哮喘患儿？**

（1）症状管理

绿区通常指哮喘症状得到了良好控制，表现为以下几个特点：

①无呼吸急促或气短：孩子在日常活动中呼吸平稳，没有感到呼吸困难。

②无咳嗽、喘息或胸闷：特别是在早晨和晚上，这些症状基本没有出现，或出现频率很低。

③运动耐受良好：孩子可以进行日常活动和适度运动，而不会因哮喘症状而感到不适。

（2）峰流速测量：使用峰流速仪可以帮助判断气道通畅程度。峰流速值应维持在个人最佳值的80%或以上。对于已知最佳峰流速的患儿，这个数值可以作为一个客观指标。如果家中没有峰流速仪，则医生可以在定期随访时为孩子进行检测。

（3）无需额外的快速缓解药物：在绿区的患儿通常无需使用快速缓解药物（如沙丁胺醇）来控制症状，或者使用频率极低（比如仅偶尔使用，每周不超过一次）。如果患儿在日常生活中完全不依赖此类药物，且没有出现急性症状，那么他可能处于绿区。

（4）睡眠质量：患儿在绿区时夜间症状很少，能够正常入睡且不被哮喘症状打扰。如果孩子能保持整夜安稳睡眠，并没有夜间醒来咳嗽、喘息或感到胸闷，那么他可能处于绿区。

（5）家长与患儿的日常观察：家长可以留意孩子日常的活动表现，并与医生的建议对比。记录日常症状和体征可以帮助家长更好地了解孩子的哮喘控制情况，并作为医生判断的一部分。在哮喘绿区时，患儿的日常生活应无明显受限，且能参与户外活动而不出现症状。

**问题80对处于哮喘行动计划绿区的哮喘患儿，为何仍需制定长期控制治疗方案？**

（1）预防哮喘症状复发：虽然患儿目前的症状被有效控制在绿区（即无症状或症状轻微），但哮喘本质上是一种慢性炎症性疾病。即使在无症状期间，气道仍可能存在潜在的炎症反应。长期控制治疗可以帮助维持这种低炎症状态，预防症状的加重或突然发作。

（2）改善长期预后：哮喘儿童的气道在炎症长期存在的情况下可能会发生结构性改变（即气道重塑），这会导致气道功能逐渐下降。持续的控制治疗可以减少气道炎症的反复出现，降低气道重塑的风险，从而改善患儿的长期呼吸健康预后。

（3）减少药物使用和急性发作的风险：长期控制治疗有助于稳定哮喘病情，减少急性发作的可能性，避免使用短效β2激动剂等“急救”药物的频率。这不仅有助于提高生活质量，还能减少哮喘急性发作带来的医疗费用及因急诊而产生的负担。

（4）提高生活质量：长期控制治疗可以帮助儿童保持较高的活动水平，减少因哮喘影响日常活动和运动的机会，从而提高他们的生活质量。对于正处于身体和心理发展关键期的儿童来说，维持正常的日常活动尤为重要。

（5）帮助家长和儿童理解和掌控哮喘管理：长期的控制治疗方案会培养患儿及其家属的疾病管理意识和技能，有助于在症状出现之前及时采取行动，增强自我管理的信心和能力，减少因疾病失控而带来的焦虑。

**问题81对处于哮喘行动计划绿区的哮喘患儿，如何降级治疗？**

对于处于哮喘行动计划绿区的哮喘患儿（即症状控制良好，通常不出现呼吸困难、胸闷、咳嗽等哮喘症状，并在日常活动和睡眠中无任何不适），降级治疗可以考虑逐步减少药物使用强度，以期在不影响哮喘控制的情况下减少药物的依赖和潜在副作用。降级治疗需依据哮喘指南，通常包括以下几个步骤：

（1）评估病情的稳定性：在考虑降级治疗之前，需确认患儿的哮喘症状已持续稳定至少3至6个月。如果患儿症状控制良好且未出现急性加重，则符合降级治疗的条件。

（2）制定降级治疗方案：降级治疗通常在医生的指导下进行。常见的降级步骤包括：

①减少吸入性糖皮质激素的剂量。如果患儿使用中等剂量的吸入性糖皮质激素，可以在医生的建议下逐步降低至低剂量。

②如果患儿已经达到最低的吸入性糖皮质激素剂量，可考虑逐步停止使用长效β2受体激动剂，仅保留低剂量的吸入性糖皮质激素。

③在适当情况下，医生可能会建议停止预防性药物，仅在必要时使用短效β2受体激动剂（例如沙丁胺醇）作为缓解药物。

（3）密切监测：降级治疗后，患儿的病情应密切监测。家长需注意观察是否有哮喘症状复发，如咳嗽、胸闷、气短等。如果症状再度出现，应及时与医生联系，并根据需要恢复到之前的治疗级别。

（4）教育和指导：在降级过程中，家长应接受关于哮喘控制的教育，了解常见症状和应对措施，确保家庭能够识别症状变化并及时采取行动。患儿应继续保持定期随访，以确保病情控制。

**问题82如何管理哮喘患儿，使其长期稳定在绿区？**

（1）制定个性化的哮喘管理计划

每位患儿的哮喘表现和诱发因素有所不同，因此制定一个个性化的哮喘管理计划十分重要。该计划应由儿科哮喘专科医生与家长共同制定，包括以下要点：

①日常用药：例如，低剂量的吸入性糖皮质激素 (ICS) 可以作为日常控制药物，减少气道的慢性炎症。

②发作时的用药：例如，短效 β2 受体激动剂 (SABA) 作为急救药物，用于缓解症状。

③用药剂量及时间表：根据病情调整药物剂量和频率，以维持在最低有效剂量，避免长期使用较高剂量的激素。

（2）定期监测与复诊

①定期复查：每隔 1-3 个月复诊，以评估哮喘控制情况，进行肺功能测试，确保用药效果。

②自我监测：家长可使用峰流速计 (PEF) 监测患儿的气道功能，根据读数判断是否有异常。

③症状记录：记录哮喘症状的出现频率、严重程度和诱发情况，有助于医生及时调整治疗方案。

（3）识别和管理触发因素

①过敏原：常见过敏原包括尘螨、花粉、霉菌、宠物毛发等，患儿应尽量避免接触这些过敏原。例如，使用防尘螨的床上用品，保持室内清洁、通风。

②环境污染：避免在空气质量差的天气外出，家中尽量少用香薰、烟雾等可能刺激呼吸道的物质。

③感染控制：预防感冒、流感等呼吸道感染，及时接种流感疫苗等。

④情绪管理：患儿的情绪波动可能加重哮喘发作，家长应关注患儿心理健康，避免焦虑或紧张。

（4）提高依从性

①用药教育：家长和患儿需了解哮喘药物的作用及使用方法，如正确使用吸入装置，确保药物充分到达气道。

②家庭支持：哮喘管理是一个长期过程，家长需积极配合医生建议，并在日常生活中帮助患儿养成健康的习惯。

③学校沟通：与学校老师沟通患儿的病情和应对措施，确保患儿在学校的安全。

（5）急性发作处理预案

①设立家庭应急方案：一旦出现哮喘急性发作症状，家长应及时使用 SABA 等急救药物，并在必要时联系急救服务。

②教育患儿识别发作早期信号：让患儿逐步学会辨别自己发作的早期症状，避免因延误而加重症状。

**问题83如何判断与识别处于哮喘行动计划黄区的哮喘患儿？**

在哮喘管理中，哮喘行动计划通常分为绿区、黄区和红区三个级别，每个区代表不同的哮喘控制状态。处于黄区的哮喘患儿通常表现出一些哮喘症状，但症状还没有严重到需要紧急治疗的地步。以下是识别处于哮喘行动计划黄区的哮喘患儿的一些方法：

（1）观察症状变化

①症状：患儿会表现出轻到中度的哮喘症状，这些症状通常包括频繁咳嗽、轻微喘息、胸闷或呼吸急促。与绿区的完全控制相比，这些症状会在日常活动中变得明显。

②夜间症状：患儿可能在夜间出现咳嗽或呼吸急促，影响睡眠质量，这是一个进入黄区的信号。

（2）呼吸流量峰值监测

峰值流速：通过使用峰值呼气流量计（PEFR），可以客观地评估肺功能。通常来说，当患儿的峰值流速下降到个人最佳流速的50%-80%之间时，表明哮喘处于黄区。

（3）使用短效支气管扩张剂的需求

药物使用：如果患儿需要使用短效支气管扩张剂（如沙丁胺醇）来缓解症状，且这种需求比平时增加，表明哮喘可能处于黄区。这表明基础药物控制可能不足，需要更密切的观察或调整治疗方案。

（4）耐力下降

活动能力：如果患儿在进行日常活动（如跑步或玩耍）时，表现出比平时更容易疲惫或喘息，说明哮喘控制可能不稳定，处于黄区。

黄区管理策略

当哮喘患儿进入黄区时，通常建议采取以下措施来避免病情进一步加重：

（1）增加支气管扩张剂使用：按照医生建议使用短效支气管扩张剂，通常每4-6小时一次。

（2）增加基础治疗：在医生指导下，可能会增加吸入性糖皮质激素的剂量。

（3）监测病情：密切观察患儿的症状、呼吸流量峰值，以及药物反应。如果症状持续超过24小时，或在使用药物后没有改善，应立即联系医生。

**问题84如何应用PEF监测处于黄区的哮喘患儿？**

对于处于黄区（即哮喘症状较严重但未到急性加重的程度）的哮喘患儿，峰流速（PEF）监测是有效的管理工具，有助于帮助家属和临床医生密切观察病情变化，及时调整治疗方案。

（1）黄区定义及PEF的作用

①哮喘黄区一般指患儿的PEF值在个人最佳值的50-80%之间。此时哮喘症状有所加重，但未达到急性加重的红区标准。

②通过PEF监测，家属和临床医生可以动态评估哮喘病情，及时判断是否需要药物调整或进一步就诊。

（2）PEF监测的具体步骤

①每日监测频率：建议每日早晚各测量一次，记录PEF值，以观察患儿的呼吸情况变化。

②记录数据：持续记录PEF测量结果，包括测量时间、PEF值和症状情况。这样可以建立一份病情变化的追踪记录，便于医生在随诊时了解病情波动趋势。

③监测环境：保持测量环境的安静和空气流通，以保证患儿在稳定状态下进行呼吸测试，减少数据误差。

④与症状对照：将PEF数据与患儿的实际症状进行对比，评估呼吸困难、胸闷等症状的严重程度。

（3）采取的措施

①药物调整：根据医生的指导，适当增加快速缓解药物的剂量，如短效β2受体激动剂，以帮助缓解症状。

②避免诱因：避免患儿接触可能诱发哮喘的因素，如过敏原（尘螨、宠物、花粉）、烟雾、寒冷空气等。

③观察病情变化：通过连续的PEF监测观察患儿是否有改善迹象。如果PEF值持续低于80%，应及时联系医生。

（4）警惕病情加重的信号

如果患儿的PEF值持续下降并接近红区标准（低于50%），或症状突然加重（如持续性咳嗽、气促），家属应尽快带患儿就医。

（5）教育家属及患儿

①正确使用PEF仪：确保家属和患儿能够正确使用峰流速仪，理解测量结果的意义。

②哮喘控制计划：帮助家属理解哮喘行动计划，在黄区时如何根据医生建议调整治疗，并在必要时进入医院检查。

**问题85处于黄区的哮喘患儿应首先使用哪些药物？如何使用？**

处于黄区的哮喘患儿通常症状较轻，但已经出现哮喘加重的迹象，因此需要采取适当的药物干预以防止症状进一步恶化。黄区的哮喘管理建议如下：

（1）快速缓解药物（SABA，短效β2受体激动剂）

①推荐药物：沙丁胺醇（如万托林 Ventolin）或特布他林（Bricanyl）气雾剂。

②使用方法：对于大多数儿童，建议使用带储雾罐的气雾剂，以确保药物吸入效果。使用剂量一般为1-2次喷雾，间隔4-6小时一次，直至症状缓解。

（2）吸入性糖皮质激素（ICS）增加剂量

①推荐药物：如果患儿平时已经在使用低剂量ICS（如布地奈德、氟替卡松），在哮喘急性加重的黄区阶段可以考虑短期增加剂量。例如，布地奈德（Pulmicort）剂量增加至原剂量的两倍，使用7-10天。

②使用方法：与日常吸入剂量保持一致的吸入方法，确保儿童正确使用吸入器具（如干粉吸入器或储雾罐）。

（3）其他口服药物

如果患儿症状在短效β2激动剂和增加吸入性糖皮质激素后仍未完全缓解，临床医生可能会考虑短期口服糖皮质激素（如泼尼松）作为辅助，但应根据专业医生的建议进行，不建议家庭随意使用。

使用指导

（1）确保患儿的父母或监护人熟悉药物使用方法，特别是如何正确使用储雾罐、干粉吸入器等设备。

（2）密切观察患儿的症状，如呼吸困难、胸闷等症状缓解情况。

（3）如果症状没有缓解或恶化，应立即联系医生，可能需要进入红区管理甚至前往急诊。

**问题86如何判断黄区患儿的病情得到有效缓解？**

在哮喘分级管理中，“黄区”通常代表病情加重但尚未到达严重程度的状态。判断黄区患儿的病情是否得到有效缓解，可以通过以下几方面的指标来评估：

（1）症状的缓解：患儿在黄区状态下可能出现咳嗽、喘息、胸闷等症状。有效缓解的标志是这些症状逐渐减轻，患儿的呼吸平稳，咳嗽减少，尤其是夜间症状减少。

（2）肺功能指标改善：家长可在医生指导下使用峰值流速仪来监测患儿的肺功能。如果患儿的峰值呼气流速（PEF）逐渐接近个人最佳值的80%或以上，说明肺功能正在恢复。

（3）用药需求的减少：在黄区阶段，患儿可能需要增加快速缓解药物的使用，如短效β2激动剂（SABA）等。如果病情得到有效缓解，患儿对这类药物的需求会减少，逐步恢复到日常维持药物即可控制的状态。

（4）活动耐力的恢复：病情好转的患儿可以逐渐恢复到正常的活动水平，不再因喘息或呼吸困难而限制活动，能够参与日常活动而不出现哮喘症状。

（5）医师评估：若有条件，建议定期到医院复诊，由医生进行专业的评估，判断是否可以恢复至“绿区”状态。

**问题87黄区的患儿在什么情况下必须立即去医院就诊？**

黄区是哮喘管理的黄色预警区域，通常表明孩子的哮喘症状有所加重，但还未达到红色紧急区的严重程度。对于处于黄区的患儿，以下情况提示需要立即去医院就诊：

（1）短效支气管扩张剂无效：如果患儿使用短效支气管扩张剂（如沙丁胺醇）后，症状没有明显缓解，说明气道狭窄程度可能较为严重，需要专业的医疗干预。

（2）症状持续加重：即便在家按指导剂量使用了哮喘急救药物，但患儿的症状（如咳嗽、喘息、呼吸急促等）仍在加重，甚至出现胸闷感加剧或呼吸困难的迹象，这表明病情进展，需要及时就医。

（3）呼吸急促：如果患儿的呼吸频率明显加快，伴有吸气时肋间凹陷或胸廓起伏明显加大，这表明呼吸系统负担加重，可能会导致缺氧。

（4）口唇发紫或指端青紫：出现这些症状说明氧气供给不足，属于较严重的缺氧表现，应尽快送医处理。

（5）不能正常进食或饮水：患儿由于喘息和呼吸困难，无法正常进食或饮水，可能导致脱水或能量不足，使情况恶化。

（6）患儿过于焦虑或异常困倦：患儿如果表现出焦虑、神志不清，或反应迟钝等，可能是缺氧或呼吸困难导致，需要紧急处理。

（7）夜间症状显著加重：哮喘症状在夜间明显加重，导致患儿无法正常入睡，可能预示病情不稳定，需前往医院进一步评估。

**问题88对于处在哮喘行动计划黄区的哮喘患儿，如何升级治疗？**

（1）确认哮喘症状

①观察症状：黄区通常表示哮喘症状有所加重，但尚未达到严重程度。典型症状可能包括咳嗽、喘息、呼吸困难或胸闷加重，且无法完全通过常规日用药物控制。

②自我监测峰流速：若患儿能够配合，可使用峰流速仪来监测呼吸功能。若峰流速低于个人最佳状态的50%-80%，表明需要进一步干预。

（2）加强短效β2受体激动剂的使用

①增加药物剂量或频次：可以短期内增加短效β2受体激动剂（例如沙丁胺醇）的使用频次，例如每4小时使用一次，通常不超过24-48小时，具体频次应根据医生指导。

②注意监测效果：如果短效β2受体激动剂的使用能够有效缓解症状，患儿应持续观察并遵循医生指导，如症状无缓解或恶化，需进一步处理。

（3）考虑增加抗炎药物

①加用或增加吸入型糖皮质激素剂量：如黄区症状持续，医生可能建议暂时增加吸入型糖皮质激素的剂量，或考虑短期口服糖皮质激素。

②避免长期大剂量使用：这类药物有潜在副作用，需在医生指导下使用，并尽可能缩短使用时间以减少副作用风险。

（4）及时联系医疗专业人员

①随时咨询：家属应及时联系医生，特别是当症状在增加药物剂量后未见改善或加重时，可能需要进一步干预甚至住院治疗。

②制订个性化的行动计划：家属与医疗团队合作，确保患儿的哮喘行动计划根据黄区的变化适当调整，并定期复查。

（5）教育和心理支持

①加强家属的哮喘管理知识：帮助家属了解如何正确使用药物，识别哮喘恶化的早期症状，以便及时采取措施。

②为患儿提供心理支持：哮喘的加重可能导致患儿出现紧张或焦虑，心理支持有助于提高患儿的依从性和舒适感。

（6）后续复查与监测

①定期评估：在症状缓解后，患儿需要复查，确定是否可以恢复到原来的维持治疗剂量，或进一步调整长期治疗方案。

②记录病情变化：家属应记录患儿症状的变化和药物使用情况，以帮助医生判断治疗效果并调整方案。

**问题89如何避免过度使用速效β_2_受体激动剂？**

（1）正确识别药物作用：速效β2受体激动剂是用于缓解哮喘急性发作症状的药物，能迅速舒张支气管，但它并非治疗哮喘的长期控制药。患者和家属需要明确，速效β2受体激动剂主要用于短期缓解症状，过度使用会掩盖哮喘控制不佳的实际情况，从而影响疾病的长期管理。

（2）制定并遵循哮喘管理计划：在医生指导下，制定个性化的哮喘管理计划，包括长期控制药物的使用、速效β2受体激动剂的剂量和使用频率。在日常中，家属应严格遵循该计划，并在患儿哮喘症状变化时及时与医生沟通。

（3）使用长期控制药物：长期控制药物（如吸入性糖皮质激素）有助于减少气道的慢性炎症，从而降低急性发作的风险，减少速效β2受体激动剂的需求。坚持使用长期控制药物，能显著改善哮喘的整体控制，避免频繁依赖速效药物。

（4）监测哮喘症状和药物使用频率：家属可通过哮喘日记记录儿童的症状、药物使用频率、触发因素等。如果速效β2受体激动剂的使用频率超过了医生的建议（例如每周使用超过2次），这表明哮喘控制可能不佳，需及时就医调整治疗方案。

（5）避免接触诱发因素：一些环境因素如过敏原（如尘螨、宠物皮屑）、污染、气候变化等可诱发哮喘。通过减少接触这些诱发因素，可以有效降低哮喘急性发作的风险，从而减少速效β2受体激动剂的使用。

（6）教育患儿和家属正确的吸入技巧：速效β2受体激动剂的效果与正确的吸入技巧密切相关。家属应确保患儿掌握正确的吸入方法，并定期检查其使用方法，以确保药物能够充分发挥作用。

（7）定期随访并评估哮喘控制水平：定期随访可以帮助医生评估哮喘控制水平，调整治疗方案，从而减少速效β2受体激动剂的依赖。定期的肺功能测试等检查能帮助监测哮喘的发展，防止病情加重。

**问题90如何判断与识别处于哮喘行动计划红区的哮喘患儿？**

（1）呼吸困难加重

红区的哮喘患儿通常表现出明显的呼吸困难，甚至在安静状态下也难以缓解。常见表现包括：

①呼吸频率显著增加，伴有明显的胸部凹陷（即在吸气时胸壁下部或肋骨间隙下陷）。

②患儿可能出现呼气时间延长，尤其在试图说话、进食或进行轻微活动时出现呼吸急促或困难。

（2）哮鸣音（喘息）明显加重

哮鸣音是判断哮喘严重程度的重要指标。红区的患儿通常会有：

①哮鸣音明显加重，听诊时全肺野均可听到显著的喘息声。

②无论使用急救药物是否见效，喘息的严重程度仍未减轻。

（3）口唇发绀（紫绀）或皮肤苍白：由于缺氧，红区哮喘患儿可能会出现口唇、指尖和面部苍白或发紫。这是哮喘加重、氧气供应不足的迹象，表明需要紧急干预。

（4）短效β2受体激动剂无效：如果哮喘患儿在使用短效β2受体激动剂（例如沙丁胺醇）后仍未见明显好转，表明哮喘控制效果不佳，进入红区的可能性较大。

（5）其他相关症状

此外，红区患儿可能会出现如下症状：

①胸痛或胸部压迫感

②精神状态改变，例如嗜睡、极度疲惫或烦躁不安

③无法完成简单的日常活动

临床干预

如果哮喘患儿符合上述多个症状，应立即采取以下措施：

（1）立即使用急救吸入药物，按医生建议剂量给药，并密切观察症状变化。

（2）如症状无缓解，或症状继续加重，立刻联系急救中心或前往急诊。

哮喘行动计划的红区判别标准

在哮喘行动计划中，红区通常表明哮喘已经达到严重发作的阶段，不能依赖常规的缓解药物，应立即寻求急救治疗。家长和监护人应严格遵循医疗建议，并确保患儿随时备有急救药物。

**问题91如何避免哮喘急性严重发作？**

（1）遵从医生的治疗方案：儿童哮喘患者应定期服用医生处方的长期控制药物，这些药物能够帮助控制哮喘症状，减少发作风险。家长应确保孩子按时按量服药，不得擅自停药或调整剂量。

（2）避免触发因素

了解并尽量避免可能诱发哮喘的触发因素，这包括：

①过敏原：如尘螨、花粉、宠物毛发、霉菌等。

②环境污染：尽量减少暴露在空气污染严重的环境中，外出时可以考虑佩戴口罩。

③烟雾和刺激性气体：避免接触烟草烟雾、香烟或其他刺激性气体。

④感染：病毒感染（如感冒）常常是哮喘急性发作的原因，因此，注重卫生，避免感冒等感染源。

（3）定期监测呼吸情况：家长可以通过峰流速仪或其他监测工具，定期测量孩子的呼吸状况。峰流速监测可以帮助提前发现哮喘加重的早期信号，便于及时调整治疗方案。

（4）学会识别早期症状：教育孩子和家长学会识别哮喘急性发作的早期症状，如胸闷、气短、咳嗽、夜间哮喘等。早期干预（例如使用缓解药物）可帮助阻止病情进一步恶化。

（5）制定并遵守哮喘管理计划：医生通常会为哮喘患者制定个性化的哮喘管理计划，包括日常用药、急性发作处理方案等。家长应确保孩子及家庭成员熟悉该计划，并在急性发作时严格按照计划执行。

（6）及时接种疫苗：流感疫苗和肺炎疫苗可减少呼吸道感染的风险，从而降低诱发哮喘急性发作的可能性。家长可以咨询医生，按时为孩子接种相关疫苗。

（7）定期复诊：即使病情稳定，定期复诊也是必要的。医生可以通过复诊评估病情、调整药物剂量和提供个性化的健康指导。

（8）学习使用急救药物

在急性发作时，快速缓解药物（如短效β2受体激动剂）可帮助迅速缓解症状。家长和孩子应学习如何正确使用这些急救药物，如定量吸入器或雾化器，并随身携带，特别是在外出时。

**问题92哮喘患儿家中或身边应常备哪些急救药物？**

（1）短效β₂受体激动剂 (SABA) 气雾剂：例如沙丁胺醇（Ventolin）或特布他林（Bricanyl）。这些药物能在几分钟内扩张支气管，是哮喘急性发作时的首选药物，用于迅速缓解气道痉挛，帮助患儿更顺畅地呼吸。气雾剂通常搭配储雾罐（例如 spacer）使用，尤其适用于小儿童，能帮助药物有效吸入肺部。

（2）口服或注射的类固醇药物：例如泼尼松（Prednisone）或地塞米松（Dexamethasone）。在严重的哮喘发作或症状加重时，医生可能建议短期使用类固醇以减少气道炎症。这类药物通常需在医生的指导下使用，避免长期服用以防止副作用。

（3）长效控制药物备用：虽然长效控制药物（例如吸入性糖皮质激素或联合治疗的ICS-LABA药物）通常是每天固定使用以预防哮喘发作，但若患儿在使用中出现加重的急性发作，应继续每日使用，并结合SABA的急救药物。长效药物需要严格遵循医嘱，不能随意增加剂量。

（4）抗过敏药物（视情况备有）：对于有过敏性哮喘的患儿，可能会同时备有抗组胺药（如氯雷他定、非索非那定）以缓解因过敏引发的症状，但并不能作为哮喘急性发作的首选药物，仅作为辅助。

注意事项

（1）定期检查药物有效期，确保所有药物在有效期内，特别是急救药物。

（2）急救方案与用药指南：家长或看护人需了解哮喘急性发作的正确用药方法，特别是SABA气雾剂的使用技巧。与儿科医生一起制定应急方案（Asthma Action Plan），明确何时使用急救药物，何时寻求医疗帮助。

（3）定期随访与评估：哮喘患儿的病情可能随着时间变化，应定期到医院复诊，调整药物剂量及治疗方案，确保患儿的哮喘得到有效控制。

**问题93如何对处于红区的哮喘患儿进行家庭急救？**

（1）立即使用急救药物

①短效β2受体激动剂：快速起效的药物（如沙丁胺醇气雾剂）应立即使用。这种药物可以松弛气道平滑肌，缓解气道收缩。标准剂量为每20分钟1次，连续3次。

②吸入方法：使用气雾剂时，建议使用带面罩的储雾罐，以确保药物正确进入患儿的气道。每次使用后，观察患儿的反应。

（2）评估症状反应

如果在使用急救药物后，症状没有明显改善，或者哮喘症状持续加重，例如呼吸急促、嘴唇或指甲发青、言语困难，可能需要立即前往急诊。患儿家属应密切观察患儿的症状反应，并及时采取措施。

（3）保持冷静，帮助患儿放松

患儿哮喘急性发作时通常会感到紧张和害怕，这会进一步加重症状。家属应尽量保持冷静，引导患儿深呼吸，缓解他们的紧张情绪，帮助其放松，以减少因焦虑引发的呼吸困难。

（4）尽可能让患儿坐直，保持呼吸通畅

患儿可以坐在椅子上，身体稍微向前倾斜，这种姿势有助于保持气道的开放。尽量避免平卧，因为这可能会加重呼吸困难。

（5）避免过度活动

哮喘急性发作期间，任何剧烈活动都可能加重症状，患儿家属应确保患儿保持安静和休息。

（6）立即寻求医疗帮助

如果哮喘发作严重且急救措施效果不佳，必须立即联系急救服务（如拨打急救电话），并将患儿迅速送往医院。在等候急救服务到达时，可以继续按医嘱使用急救药物。

注意事项：

（1）若患儿的哮喘管理方案中有口服类固醇药物（如泼尼松），并已由医生建议在严重发作时使用，应按医生指导的剂量服用。

（2）家属在采取上述步骤时，应根据患儿的个体情况进行调整。如果患儿的症状与以往发作时不同，或者之前的急救药物使用后效果不佳，应更快地寻求专业医疗帮助。

**问题94红区哮喘患儿急诊就医过程中有哪些注意事项？**

在红区（严重发作）哮喘患儿的急诊就医过程中，以下几点非常重要，能够帮助临床专家和患者家属有效管理和应对急性哮喘发作。

（1）保持冷静，尽快前往急诊

①立即就医：当患儿处于红区，症状严重且呼吸急促或困难，应立刻前往急诊，避免自行处理或延误治疗。

②保持冷静：家长需保持冷静，避免引起患儿过度紧张，因为情绪紧张可能加重呼吸困难。

（2）使用急救药物（如速效支气管扩张剂）

①使用支气管扩张剂：在前往急诊途中或等待就诊时，可以在医生指导下使用速效支气管扩张剂（如沙丁胺醇）。每4小时一次，观察效果是否显著。

②正确使用设备：确保吸入装置（如定量气雾剂或雾化器）使用正确，以使药物充分吸入，避免浪费。

（3）观察并记录症状

①监测呼吸状况：家长需密切观察患儿的呼吸情况，包括呼吸频率、胸部起伏等是否有明显变化，以便向医生提供准确的信息。

②记录使用药物的时间和剂量：记下急救药物使用的时间、剂量和效果，以便医生在急诊时快速了解病情。

（4）提供充足氧气

①保持通风良好：在条件允许下，尽量保持通风良好，以增加空气流通，提供足够氧气。

②避免诱发物质：远离可能的诱发因素（如香烟烟雾、强烈的气味或过敏源）。

（5）确保随身携带病历和药物清单

①病历和药物清单：携带患儿的病历记录，尤其是包括以往的哮喘诊断、药物过敏史和当前用药情况，以便医生全面评估病情。

②备有备用药物：尽量随身携带备用药物，尤其是急救药物，以备不时之需。

（6）注意避免食物和饮料摄入

避免饮食：在严重哮喘发作期间，尽量避免饮食，尤其是固体食物，以免呛咳引发窒息。

（7）及时告知医生患儿的过敏史

告知过敏史：如果患儿有过敏史，家长需在急诊时第一时间告知医生，以防在治疗中使用不适合的药物。

（8）保持随时和医生沟通

①清晰描述病情：家长在急诊时要尽量准确、清晰地描述患儿的病情，包括病情变化、用药效果等，以便医生制定合适的治疗方案。

②听从医生建议：配合医生的检查和治疗，不要随意更改治疗方案或药物使用剂量。

（9）紧急处理后继续进行后续护理

①继续观察：急诊处理结束后，家长应继续观察患儿的症状，防止病情反复。

②遵医嘱安排随访：急性期后，应安排适当的复查，配合医生评估治疗方案，以减少再次发作的风险。

**问题95红区哮喘患儿使用全身性糖皮质激素有哪些注意事项？**

（1）用药时机与剂量

①及时使用：当哮喘患儿症状显著加重，出现重度喘息或使用常规支气管扩张剂（如沙丁胺醇）疗效不佳时，考虑启动糖皮质激素治疗。

②合理剂量：全身性糖皮质激素的剂量应根据患儿的体重、症状严重程度及医生的建议进行调整。通常建议使用最低有效剂量，避免过量使用，以减少不良反应的发生。

（2）用药时间与疗程

①疗程控制：急性发作期用药一般建议短期使用，全身性糖皮质激素的疗程通常在3-5天内。如果症状缓解迅速，可能无需继续使用；若症状未得到有效控制，应在医生指导下评估是否需要延长疗程或调整治疗方案。

②逐步停药：虽然急性治疗时间较短，但如果使用超过一周，建议在医生指导下逐步减量，避免突然停药，以预防肾上腺皮质功能抑制等症状的发生。

（3）监测副作用

①短期副作用：儿童使用全身性糖皮质激素可能会出现短期副作用，如胃肠不适、情绪波动（如易怒、焦虑）、睡眠障碍及血糖水平波动等。密切观察孩子的情绪和行为变化，必要时调整饮食，减少高糖食物。

②长期副作用：尽管急性加重期的用药时间较短，但若频繁使用全身性糖皮质激素，有可能影响儿童的生长发育，增加感染风险。因此，建议每次使用后复查，评估其体重、身高及感染状况等，记录在案以便长期跟踪。

（4）药物相互作用

注意药物交互作用：一些药物可能与糖皮质激素产生交互作用，如非甾体抗炎药、某些抗生素等。使用糖皮质激素期间，避免自行使用其他药物，尤其是非处方药，以防发生药物相互作用。

（5）定期随访与家属教育

①随访复查：患儿使用全身性糖皮质激素后，应定期进行随访，检查用药效果和副作用。家属需详细记录孩子的症状变化、用药情况，并按时与医生复诊。

②家属教育：教育家属正确认识糖皮质激素的作用和副作用，理解治疗的必要性，以提高依从性。家属需了解何时可以逐步停药，何时应立即复诊，避免盲目延长疗程。

**问题96对于反复处于红区的哮喘患儿，如何进行治疗方案的调整及长期管理？**

1. 评估目前的治疗方案

重新评估哮喘控制情况：红区状态通常指哮喘严重发作，可能由以下因素引起：

（1）基础药物不足：维持治疗药物剂量不足或使用不规律。

（2）触发因素未控制：接触过敏原（如尘螨、花粉等）或其他诱发因素（如烟草烟雾、冷空气、病毒感染）。

（3）吸入技术问题：很多患儿使用吸入装置存在问题，导致药物未充分吸入。

建议在调整治疗方案前，详细评估患儿的病史、生活环境和诱因管理情况，并检查吸入技术是否正确。

2. 加强基础治疗

（1）调整药物剂量：对于反复进入红区的患儿，通常需要强化吸入性糖皮质激素（ICS）剂量，并考虑联合长效β2受体激动剂（LABA）或白三烯受体拮抗剂（如孟鲁司特），根据哮喘严重程度和年龄调整剂量。例如：

①中剂量或高剂量ICS：适用于未能在低剂量ICS或低剂量ICS+LABA下获得控制的患儿。

②ICS + LABA联合用药：对于6岁以上患儿，可在中至高剂量ICS基础上添加LABA。

（2）短期强化疗法：当患儿处于红区时，可以短期增加ICS剂量（如加倍），或使用口服糖皮质激素（如泼尼松龙），视发作严重程度和临床指征决定短期用药的持续时间（一般不超过5-7天）。长期口服糖皮质激素应慎用，因其副作用较大，仅适用于严重病例。

3. 管理和预防触发因素

环境控制：家属应尽量减少孩子接触已知过敏原和其他触发因素，例如：

（1）保持家中无烟环境：烟草烟雾会加重哮喘症状，尤其在儿童中。

（2）减少过敏原暴露：如尘螨、宠物毛发、霉菌等。使用空气净化器、定期清洗床上用品、保持室内通风等措施可以有效降低过敏原。

（3）预防感染：流感和其他呼吸道感染是常见的哮喘诱因，建议按时接种流感疫苗。

4. 定期监测和随访

（1）使用峰流速计监测：在家中监测患儿的峰呼气流速（PEF），可以帮助识别症状恶化趋势，建议家属每天记录峰流速，尤其是早晚两次测量，以便及早发现病情变化。

（2）定期随访：患儿需要每3-6个月进行哮喘随访，确保病情稳定，同时让医生根据患儿的具体情况对治疗方案进行调整。对于控制不佳的患儿，随访频率可以增加至每月一次，直到病情稳定。

5. 制定个性化的哮喘行动计划：制定一份详细的哮喘行动计划可以帮助家属更好地管理患儿病情。该计划应包括：

（1）红区、黄区和绿区的定义和处理措施：让家属能够识别和应对不同严重程度的哮喘发作。

（2）药物使用指南：包括维持药物、急救药物及其剂量和使用频率。

（3）紧急措施：在哮喘急性发作时，家属应知道何时使用急救药物（如沙丁胺醇）以及何时需要紧急就医。

6. 心理支持与教育：患儿反复进入红区会引发家属的担忧，需提供心理支持和教育：

（1）家庭教育：帮助家属了解哮喘的病理机制、药物作用及其副作用，从而增强对哮喘管理的信心。

（2）心理支持：患儿在反复发作后可能产生焦虑情绪，适当的心理干预可以减轻他们对哮喘发作的恐惧，避免情绪影响病情。

7. 持续关注成长发育：长期使用糖皮质激素可能影响儿童的生长发育，因此在随访过程中应关注患儿的身高和体重发育情况，必要时可以请儿科内分泌医生进行评估，调整治疗方案，以平衡哮喘控制和发育之间的关系。

8. 考虑专科转诊：如果患儿在积极治疗和管理后仍频繁进入红区，建议转诊至哮喘专科（如儿科哮喘中心或呼吸专科），进一步检查是否存在其他基础疾病（如过敏性鼻炎、胃食管反流等），并通过气道炎症监测（如呼出气一氧化氮测定）来优化治疗方案。

**问题97如何在医院开展儿童哮喘的健康教育、推行CCAAP？**

1.建立系统化的健康教育方案

（1）多学科团队协作：首先，应由医生、护士、呼吸治疗师、营养师等组成多学科团队，共同制定和实施儿童哮喘健康教育计划。这样可以确保教育内容的专业性、全面性和一致性。

（2）分阶段教育：根据儿童哮喘患者的年龄和病情严重程度，将教育内容分为基础知识、病情自我管理、药物使用与调整等阶段，逐步引导家长和患者理解和掌握哮喘管理技能。

（3）制定个性化教育计划：在开展教育时，应针对每个患儿的实际情况制定个性化的教育计划，包括对药物敏感性、过敏原、环境因素等进行评估，以便提供更有针对性的建议。

2.关键教育内容

（1）哮喘基础知识：

①讲解哮喘的病理生理机制，包括气道炎症、气道高反应性等。

②阐述哮喘的典型症状，如咳嗽、喘息、气促和胸闷，帮助家长和患者识别和管理哮喘症状。

（2）触发因素管理：

①介绍常见的哮喘触发因素，如过敏原（尘螨、花粉、霉菌等）、感染、气温变化、运动和情绪波动。

②教授避免接触或减少暴露的方法，如清洁环境、使用空气过滤装置、避免烟雾等。

（3）药物管理与使用技巧：

①讲解哮喘的控制药物（如吸入型糖皮质激素）和缓解药物（如短效β₂受体激动剂）的作用机制和使用方法。

②教授吸入装置的正确使用方法，如雾化器、定量吸入器（MDI）和干粉吸入器（DPI）等的使用技巧，确保药物能够有效到达气道。

（4）日常监测与自我管理：

①通过训练家长和患儿了解如何使用峰流速仪监测气道情况，并根据峰流速的变化调整管理措施。

②教育家长和患儿如何根据哮喘控制等级（绿、黄、红灯区）判断病情，以便及时采取措施。

（5）应急处理方案：为患儿制定个性化的应急处理计划，包括加重期的药物使用方案和紧急就医指引，确保家长在病情急性加重时能够正确、及时地进行处理。

3.推行CCAAP的具体措施

①提供书面和电子版哮喘行动计划：为每位患儿和家长提供书面和电子版的CCAAP，详细列出管理措施和应急处理方案。这些计划应包括绿、黄、红灯区的说明和相关指导，以便家长随时参考。

②定期复诊和随访：通过建立哮喘管理档案，安排患儿的定期随访评估。利用随访机会进一步强化教育内容，对家庭管理策略进行检查和调整，确保管理计划的执行效果。

③推广教育资源：

在医院内设立哮喘教育中心，提供教育资料、视频课程和呼吸训练等。

提供在线支持平台，家长和患儿可以通过APP或网站获得即时的教育资源和专家建议。

④家庭和学校合作：向家长和患儿的学校提供哮喘管理的知识分享与指导，以确保儿童在学校环境中也能得到适当的支持。学校护士和教师也应掌握基本的哮喘应急知识，以便在突发情况下提供初步帮助。

4.持续评估与改进

①效果评估：定期评估哮喘健康教育和CCAAP的实施效果，如患者的哮喘控制率、急性加重次数、急诊就医率等，通过这些指标判断教育和管理计划的有效性。

②反馈机制：收集家长和患者对健康教育内容和CCAAP的反馈，分析实际操作中的问题，适时进行改进，以提高教育和管理方案的可行性和有效性。

5.常见临床问题的纠正与答疑

针对家长常见的误区，如过度依赖缓解药、错误的吸入技术等，多次复习和讲解这些知识点，纠正错误观念和做法。同时，随时回答家长和患者的疑问，以增强他们对哮喘管理的信心。

**问题98如何在社区开展儿童哮喘的健康教育及防治工作？**

1.提高家长和儿童的哮喘健康意识

（1）哮喘的基本知识宣传：在社区内通过讲座、宣传手册、海报等方式普及儿童哮喘的病因、诱发因素和症状，使家长了解哮喘的慢性特性及规范管理的必要性。

（2）病情识别与应对教育：教导家长如何识别哮喘的常见症状（如喘息、气促、胸闷和咳嗽等），特别是在夜间或清晨加重的表现。同时讲解儿童哮喘急性发作时的处理方法，如保持患儿镇定、按医嘱使用缓解药物等。

2. 开展哮喘管理培训

（1）个体化的哮喘管理计划：为每个哮喘患儿制定个性化管理计划，内容包括日常管理、急性发作的处理方案、用药方案等。特别注意规范吸入剂的使用方法，避免剂量错误或使用不当。

（2）用药教育：针对家长和儿童，详细说明哮喘长期控制药物（如吸入性糖皮质激素）的使用必要性及缓解药物的作用，帮助家长和儿童理解如何坚持使用控制药物以预防发作、使用缓解药物在急性期缓解症状。正确示范吸入装置（如干粉吸入器或气雾剂吸入器）的使用方法，确保患儿能有效吸入药物。

3. 环境控制与过敏原管理

（1）室内环境管理：建议家庭保持居住环境整洁，减少尘螨、霉菌、宠物毛发等常见过敏原，避免二手烟和其他刺激性气味。定期清洗家中容易积聚过敏原的物品，如床单、窗帘和地毯。

（2）减少户外诱发因素：在天气状况（如重污染、花粉高峰期）不利于哮喘患者出行时，建议尽量减少户外活动，并在必要时佩戴防护口罩。此外，避免让儿童参加剧烈运动或暴露于强烈冷空气中，以减少诱发哮喘发作的风险。

4. 建立随访制度和应急联络网络

（1）定期随访：鼓励哮喘儿童的家长按时带患儿至社区卫生服务中心或专科医院进行定期随访。随访内容包括症状评估、肺功能检查、用药依从性检查等，有助于根据病情变化调整治疗方案。

（2）急性发作应急预案：在社区内建立儿童哮喘急性发作的应急联系网络，包括医生、家长、学校和社区工作人员，以便在紧急情况下能及时联络并提供帮助。

5. 社区与学校合作

（1）学校的哮喘管理支持：与学校合作，向教师和校医提供哮喘管理的基本培训，确保哮喘儿童在学校能得到必要的关注和支持，尤其在体育课等容易诱发哮喘的场合。

（2）儿童与家长的教育：定期举办面向患儿及其家长的哮喘知识课堂和管理技能培训，鼓励儿童了解哮喘，学习自我管理，帮助他们应对和减少哮喘发作的心理压力。

6. 引入健康管理工具与技术支持

（1）智能健康工具：通过手机APP或智能设备记录哮喘症状、用药情况、肺功能数据等，帮助家长和医疗人员随时掌握儿童的病情变化，方便在社区开展更为便捷的健康管理。

（2）远程健康指导：社区卫生服务中心可以通过电话、微信或网络平台，为家长提供远程哮喘管理指导，解答日常疑问，并督促家长和患儿按计划用药和定期复诊。

7. 建立哮喘家长互助小组

支持与交流：在社区内组织哮喘患儿家长的支持小组，定期交流哮喘管理经验，分享育儿心得，以减少家长在哮喘护理中的孤独和无助感，增强家长的信心。

**问题99如何在校园做好儿童哮喘的健康教育及防治工作？**

1. 加强儿童、家长及教职工的哮喘健康教育

（1）举办哮喘知识讲座：定期为学生、家长和教师组织哮喘知识讲座，介绍哮喘的基础知识、诱发因素、典型症状和应对方法。尤其针对哮喘患儿所在班级的教师和家长，讲解哮喘急性发作时的识别与初步处理。

（2）分发哮喘管理手册和宣传资料：发放简明易懂的哮喘知识手册和资料，内容包括常见诱因、急性发作的应对措施、正确使用吸入器等，使学生和家长能够掌握哮喘的基本知识。

（3）利用校内媒介：通过校园广播、公告栏、微信公众号等多种媒介宣传哮喘的健康知识。通过多渠道、持续性的宣传，可以提高学生、教师、家长对哮喘的关注度和防治意识。

2. 建立学生个性化的哮喘管理计划

（1）制定个性化健康档案：学校医务室或校医可根据患儿的病史和医生建议，为哮喘患儿建立健康档案，记录哮喘严重程度、触发因素、日常药物使用及注意事项。档案需定期更新并得到学校管理层的支持和保存。

（2）配合家庭与医院的管理计划：在患儿监护人的协助下，学校应与患儿的主治医生沟通，获得并了解儿童的哮喘控制方案，确保校医或教职人员掌握急性发作时的急救流程，并按计划提醒患儿按时用药。

（3）教会患儿正确的用药技巧：确保患儿掌握吸入剂、干粉吸入器或其他药物装置的正确使用方法。可以组织哮喘患儿和家长共同参与吸入器使用培训，确保患儿在没有家长陪同时也能有效使用药物。

3. 创设安全、无刺激的校园环境

（1）减少诱发因素：避免在教室内使用容易引发哮喘的化学品、清洁剂或空气清新剂，减少对患儿呼吸道的刺激。对校园内种植的植物及其花期进行管理，减少花粉等常见过敏原的暴露。

（2）加强空气质量监测：建议在学校设置空气质量监测设备，尤其是在空气污染频发的地区，以便实时监控并采取措施。如空气质量较差时，可减少户外活动或调整体育课的形式。

（3）通风及清洁管理：保持教室和公共区域的通风良好，定期清洁空调系统、窗帘、地毯等容易积累尘螨和霉菌的设施，减少过敏原在室内的积累。

4. 建立快速应急响应机制

（1）制定哮喘急性发作处理流程：学校应有统一的急性发作应急处理流程，包括症状识别、急救步骤和用药流程。确保教师、校医及其他教职员工能够熟练掌握，以便在紧急情况下及时应对。

（2）准备急救药物和设备：在校医室常备急救药物，如急救吸入剂（例如短效β₂受体激动剂）。教师和校医应熟悉药物的使用，并在必要时为患儿使用急救吸入剂，帮助缓解急性症状。

（3）培训师生掌握急救技能：组织教职工和高年级学生进行基本的急救培训，教会他们识别哮喘急性发作的症状并协助处理，尤其是对于年幼患儿，急救员可以在专业救护人员到达之前采取急救措施。

5. 加强学校、家庭与医院的沟通协作

（1）定期沟通：学校应与家长保持密切联系，了解患儿在校外的病情控制情况，若发现异常情况，及时与家长沟通，以便早期干预和处理。

（2）促进家校医院三方管理：学校医务室、家庭医生及家长应共同参与患儿的哮喘管理，定期召开健康管理会议，更新哮喘管理方案。同时，根据季节变化和患儿的病情变化，调整校内外的防护措施。

（3）引导哮喘患儿进行自我管理：随着年龄增长，教会患儿识别哮喘症状并了解如何在发作初期进行自救，包括避开诱发因素、合理用药等。提升孩子的自我管理能力，可以显著降低发作风险。

6. 鼓励适量体育活动和健康生活习惯

（1）合理安排体育活动：哮喘儿童应在运动前做好预防工作，如按照医生建议提前使用支气管扩张药物，适度进行热身运动。教师应鼓励哮喘患儿参加体育活动，但应避免剧烈、长时间的户外运动，尤其在空气质量较差的日子里。

（2）倡导健康生活习惯：在校园教育中强调健康生活方式，如均衡饮食、充足睡眠、定期锻炼等，通过良好的生活习惯提升患儿的身体免疫力和整体健康水平。

**问题100如何利用互联网平台做好儿童哮喘的健康教育工作？**

1. 在线健康教育课程

（1）平台选择：可以通过微信公众号、健康管理App、视频平台等提供儿童哮喘相关的在线课程。课程内容包括哮喘的病理知识、症状识别、药物使用、过敏原避免、急救措施等。

（2）互动功能：通过平台的互动功能（如在线问答、评论、直播等），家长和患者可以随时向专业医生提问，解决疑虑。这样能够提高教育的互动性，帮助家长更好地理解哮喘管理的重点。

2. 定期发布科普文章和短视频

（1）文章和短视频：定期发布简明易懂的科普文章或短视频，内容涉及儿童哮喘的基础知识、药物的使用方法、环境管理、急性发作的应对措施等。这些信息可以帮助家长了解如何在日常生活中更好地管理孩子的哮喘。

（2）多媒体内容：结合图文、视频、动画等多种形式进行知识传播，能够使内容更加生动，易于理解。短视频特别适合家长在忙碌时段随时观看，增强了教育的可达性。

3. 在线健康监测与数据共享

（1）健康管理App：一些专为哮喘管理设计的手机应用可以记录孩子的症状、药物使用情况、环境暴露等数据，帮助家长及时了解孩子的哮喘状况。部分App还可以实时反馈症状变化，提醒家长按时用药。

（2）医生远程指导：家长可以通过平台与医生进行线上咨询和定期跟踪，及时调整治疗方案。医生可以根据孩子的健康数据和症状表现，提供个性化的指导。

4. 线上支持小组与社交平台

（1）家长交流群：建立线上家长支持小组（如微信群、QQ群等），让患儿家长分享彼此的经验和感受。这些小组可以成为家长们互相支持和鼓励的场所，同时也是交流育儿经验和哮喘管理技巧的有效平台。

（2）专家答疑：定期举办专家在线讲座或问答，邀请呼吸科医生、过敏免疫科专家等为家长解答困惑，提升家长的健康管理水平。

5. 远程跟踪和智能干预

（1）远程监测：通过设备（如智能吸入器、肺功能监测仪等）采集儿童的哮喘相关数据，医生可以进行远程评估和干预。这些设备通过互联网平台将数据传输到云端，医生可以实时跟踪儿童的病情变化，及时进行调整。

（2）智能提醒：平台可以通过设定提醒功能，提醒家长按时给孩子使用药物、进行复诊、避免过敏原等，减少家长的遗忘或疏漏，提高治疗依从性。

6. 个性化健康教育内容

（1）基于大数据的个性化推送：通过互联网平台收集用户（家长和患儿）的相关数据和病历信息，为每个患儿量身定制健康教育内容。例如，对于过敏性哮喘的儿童，平台可以专门推送有关过敏原管理的知识和建议。

（2）行为改变建议：平台可以通过数据分析，识别家长和患儿的行为习惯，并给出有针对性的行为改变建议（如改善饮食习惯、减少环境刺激等），帮助家长和患儿更好地应对哮喘。

7. 公益活动和线上宣传

（1）公益项目：通过互联网平台推动一些公益活动，如“哮喘管理日”活动、哮喘知识普及月等，邀请专家通过直播进行健康教育讲座，增加公众对哮喘的认知。

（2）线上宣传：结合社交媒体进行哮喘相关的公益宣传，增强公众对儿童哮喘的关注，提高早期筛查和及时治疗的意识。

**AI-Simplified Responses, AI-SRs**

**问题1什么是CCAAP？为什么要使用CCAAP？**

CCAAP（慢性病管理行动和评估计划）是一种帮助慢性病患者（如哮喘）管理健康的工具。它提供评估、个性化护理计划、监测策略和患者教育，旨在帮助患者及其家属更好地控制病情。

为什么要使用CCAAP？

①个性化管理：根据每位患者的具体情况制定护理计划，提高治疗效果。

②系统监测：通过标准评估工具，医生和患者可以更好地追踪病情变化，及时调整治疗。

③增强参与感：鼓励患者和家庭积极参与，帮助他们理解病情和管理症状。

④预防并发症：通过健康管理，及时发现潜在风险，减少急性发作和住院的可能性。

⑤提升健康知识：提供教育材料，帮助患者掌握哮喘的病因、诱因和日常管理技巧。

⑥促进团队合作：加强医生、护士和药师之间的合作，确保患者获得全面支持。

**问题2 CCAAP手机版应用程序有哪些优点？**

CCAAP 是“中国儿童哮喘行动计划”的手机版应用程序，可以帮助家长更方便地管理孩子的哮喘。它的主要优点包括：

①随时查看管理方案：家长可以在手机上查看医生为孩子制定的日常用药和急性发作处理方案，不易遗失。

②病情判断直观：应用程序通过“绿区、黄区、红区”帮助判断病情。绿区表示控制较好，黄区提示病情可能加重，红区提示病情较重，需要及时处理或就医。

③便于持续记录：可以记录孩子的哮喘症状、呼气峰流量和可能的诱发因素，帮助家长及时发现病情变化。

④辅助规范用药：家长可以按照医生预先制定的行动计划采取相应措施，减少漏药、随意停药或处理不及时的情况。

⑤方便复诊沟通：长期记录的信息有助于医生了解孩子近期的哮喘控制情况，并为调整治疗方案提供参考。

⑥提供健康教育：家长可以通过应用程序学习哮喘、吸入药物使用及日常护理等知识，提高家庭管理能力。

⑦携带和保存方便：与纸质行动计划相比，手机更便于随身携带，相关记录也更容易保存和查看。

需要注意的是，CCAAP 手机版是一种辅助管理工具，不能代替医生的诊断和治疗。孩子的红、黄、绿区标准及具体用药方案，应由医生根据病情提前制定，家长不要仅依据应用程序提示自行长期增减药物。

**问题3 什么是哮喘？**

哮喘是一种影响呼吸道的慢性病，导致气道发炎，容易对刺激物产生过敏反应。主要症状包括喘息、呼吸急促、胸闷和咳嗽，通常在晚上或清晨更明显。哮喘可能由多种因素引起，比如过敏原（如尘螨和花粉）、空气污染、病毒感染、运动或情绪变化。

①病因：哮喘的原因复杂，包括遗传和环境因素，比如空气质量差和过敏原。

②症状：常见症状是喘息、胸闷和咳嗽，不同人可能表现不同。

③诊断：医生通过询问病史、症状和进行肺功能测试来确诊。

④管理：哮喘的管理包括避免诱因、定期监测症状、使用医生开处方的药物，并制定应急计划。

⑤预防：教育患者及家属识别和处理哮喘诱因，保持健康的生活习惯，减少发作的机会。

**问题4哮喘可以遗传吗？**

①家族影响：如果家里有人患哮喘，其他人得病的风险会增加。

②环境因素：过敏原（如花粉和尘螨）、空气污染和烟草烟雾也会导致哮喘发作。

③遗传与环境：哮喘通常是遗传因素和环境因素共同作用的结果。例如，某些基因可能让人对过敏原更敏感。

④预防与管理：虽然遗传不能改变，但可以通过早期检查、避免诱因和适当治疗来有效管理哮喘。

总的来说，哮喘有一定的遗传性，但环境因素也很重要。对有家族史的人来说，及早监测和管理很关键。

**问题5儿童哮喘有哪些类型？**

儿童哮喘主要有以下几种类型：

①过敏性哮喘：最常见，通常与过敏源（如花粉、宠物毛发、尘螨）有关。

②非过敏性哮喘：不由过敏源引起，可能与病毒感染、空气污染或天气变化相关。

③运动诱发性哮喘：运动后发作，尤其在冷或干燥空气中更明显。

④夜间哮喘：症状在夜间或清晨加重，可能与生理变化或过敏有关。

⑤职业性哮喘：较少见，儿童可能因接触特定物质（如化学品）而发病。

⑥难治性哮喘：症状难以控制，需要特别的治疗。

**问题6 儿童哮喘与成人哮喘有何不同和联系？**

（1）发病原因：

①儿童哮喘：通常与过敏有关，常见过敏源有花粉、尘螨和宠物毛发。儿童的气道较小，容易堵塞。

②成人哮喘：可能与环境因素、职业暴露和吸烟有关。成人的哮喘常伴有气道变化。

（2）症状表现：

①儿童哮喘：症状包括喘息、咳嗽（特别是夜间或运动后）和胸闷，常有周期性。

②成人哮喘：症状可能更复杂，包括持续咳嗽和气短，常伴有其他呼吸问题。

（3）治疗方法：

①儿童哮喘：药物剂量通常根据体重调整，需注意药物对生长的影响。

②成人哮喘：治疗方法较灵活，可能使用更强效的药物，患者管理能力更强。

（4）心理社会因素：

①儿童哮喘：可能影响学习和社交，家长的支持很重要。

②成人哮喘：心理因素如焦虑和抑郁可能影响管理和治疗效果。

联系

（1）病理生理：两者均涉及气道的慢性炎症和气流受限等机制。

（2）治疗目标：控制症状、减少急性发作、改善生活质量和维护正常肺功能是共同目标。

（3）急性发作管理：急性发作的处理原则相似，通常使用支气管扩张剂和糖皮质激素。

（4）教育和自我管理：对患者及家属进行教育，制定管理计划和使用吸入器对双方都很重要。

**问题7儿童哮喘有哪些临床特点？**

（1）症状：

喘息：常在夜间或清晨加重。

咳嗽：多为干咳，活动后或接触过敏源时加重。

呼吸困难：活动时感到呼吸急促，严重时影响日常生活。

胸闷：部分儿童可能感到胸部紧缩。

（2）发作：

周期性：症状时好时坏，常与过敏源或病毒感染相关。

夜间加重：夜间症状常影响睡眠。

（3）诱因：

过敏源：如尘螨、花粉和动物皮屑。

环境因素：寒冷空气、烟雾等。

感染：常见的上呼吸道病毒感染。

情绪：激烈情绪或兴奋可能诱发发作。

（4）生理特点：

肺功能：表现为可逆性气道阻塞，通过支气管扩张剂可改善。

气道反应性：儿童的气道对刺激物反应更强。

（5）家族史：

许多儿童有过敏性疾病或哮喘的家族历史。

（6）合并症：

哮喘常与过敏性鼻炎、湿疹等共存。

了解这些特点有助于早期发现和管理儿童哮喘，提升他们的生活质量。

**问题8儿童哮喘如何诊断？**

儿童哮喘的诊断步骤包括几个重要部分。首先，医生会询问孩子的症状，如喘息、咳嗽和呼吸急促，了解这些症状发生的频率和诱因。然后，医生会进行体检，查看呼吸道状况。接着，可能会做肺功能测试，比如测量呼吸峰流速，以评估肺功能。此外，过敏原测试和影像学检查也可能被用来排除其他疾病。最后，医生会根据这些信息来确定是否患有哮喘，并定期随访以调整治疗方案。

**问题9婴幼儿期的反复喘息会发展为哮喘吗？**

婴幼儿期的反复喘息可能与未来哮喘有关。研究表明，早期喘息是哮喘风险因素之一，但并不是所有有喘息的婴儿都会发展为哮喘。

影响因素包括：

（1）喘息类型：病毒感染引起的喘息可能不代表未来哮喘。

（2）家族史：如果家里有哮喘或过敏史，风险会增加。

（3）环境因素：接触过敏原（如尘螨和烟雾）会提高风险。

（4）健康问题：如过敏性鼻炎或湿疹也可能关联。

管理建议：

定期就医：与医生合作，监测症状。

避免触发因素：减少接触过敏源。

健康教育：了解哮喘的早期迹象和管理方法。

**问题10 6岁以下儿童的哮喘如何诊断？**

（1）病史收集：询问孩子的症状，如喘息、咳嗽和呼吸急促，特别是在夜间或运动后加重的情况。确认是否有家族哮喘或过敏史。

（2）体格检查：医生检查孩子的呼吸音，寻找喘鸣音或其他异常。

观察孩子是否有呼吸困难的迹象，比如鼻翼扇动。

（3）症状监测：记录孩子的症状、药物使用和触发因素（如过敏原、运动或感染）。

（4）肺功能测试：对于小儿童，肺功能测试可能有限，但可以使用简单的设备评估气道通畅度。

（5）过敏原检测：可考虑进行皮肤测试或血液检查，以找出过敏原。

（6）排除其他疾病：确保没有其他可能导致类似症状的疾病，如感染或异物吸入。

（7）试用药物：医生可能会给孩子试用支气管扩张剂，观察是否改善症状。

与家长沟通非常重要，以确保症状描述准确，并制定合适的管理计划。这个过程帮助确认哮喘的诊断，确保孩子得到适当的治疗。

**问题11什么是CVA？**

CVA（不伴喘息的特殊类型哮喘）是一种哮喘，患者通常感到呼吸困难和胸闷，但不出现喘息声。其诊断标准包括：1) 反复出现的呼吸困难，2) 肺功能测试显示气流受限，3) 排除其他呼吸疾病。

治疗方法一般包括使用吸入性糖皮质激素（ICS）和长效β2-激动剂（LABA）。必要时，可以添加白三烯受体拮抗剂或生物制剂。患者还应定期监测症状和肺功能，以确保治疗有效并及时调整。

**问题12儿童哮喘要注意与哪些疾病相鉴别？**

儿童哮喘需要与以下疾病区分：

（1）过敏性鼻炎：症状包括鼻塞和流涕，容易误诊为哮喘。

（2）支气管炎：急性或慢性咳嗽和喘息可能与哮喘相似。

（3）肺炎：会导致呼吸急促和咳嗽，需通过检查确认。

（4）慢性咳嗽综合征：主要表现为持续咳嗽，可能由其他原因引起。

（5）气道异物：小孩常会误吸异物，需警惕。

（6）心脏病：某些心脏问题也能引起喘息和呼吸困难。

（7）胃食管反流病（GERD）：可能导致咳嗽和喘息。

准确的诊断需要详细了解病史、症状，并进行必要检查，以便合理管理哮喘。

**问题13哮喘的并存疾病有哪些？**

哮喘常见的并存疾病包括过敏性鼻炎、食物过敏、湿疹和胃食管反流病。研究表明，约60%至80%的哮喘患者也有过敏性鼻炎，湿疹在儿童中约占30%至50%。这些疾病会影响生活质量，并可能加重哮喘症状。因此，管理这些并存疾病对哮喘患者很重要。

**问题14 AR对哮喘有什么影响？**

变应性鼻炎（AR）与哮喘密切相关。AR会引起上呼吸道的炎症，这可能加重气道的过敏反应，从而增加哮喘发作的风险。许多AR患者也有气道高反应性，这使得哮喘症状更严重。因此，控制AR对管理哮喘非常重要。治疗方法包括抗组胺药和鼻用类固醇。有效管理AR不仅能减轻鼻部症状，还能减少哮喘的发生和严重程度。

**问题15儿童哮喘的常见检查有哪些？**

儿童哮喘的常见检查包括：

（1）病史评估：询问症状、发作频率、诱因和家族史，以了解哮喘的严重程度。

（2）体格检查：检查呼吸音、呼吸频率和有无喘息。

（3）肺功能测试：

①峰流速测定：使用峰流速计在家监测。

②肺活量测定：适合5岁以上儿童，评估肺功能。

（3）支气管激发试验：测试气道的反应性，适用于肺功能正常但怀疑哮喘的情况。

（4）过敏原检测：皮肤试验或血液检查，寻找可能的过敏源。

（5）影像学检查：有时做胸部X光，排除其他呼吸疾病。

（6）呼气一氧化氮（FeNO）检测：测量呼气中的一氧化氮，评估气道炎症。

这些检查帮助医生准确诊断和管理儿童哮喘。

**问题16为什么要测PEF？**

测量峰流速（PEF）在儿童哮喘管理中非常重要，主要有以下几点原因：

（1）监测病情：PEF能帮助患者和家属了解哮喘控制情况，及时发现肺功能变化。

（2）个性化管理：每个儿童的哮喘不同，PEF测量能帮助医生制定适合的治疗方案。

（3）激励患者：定期测量PEF可以让儿童看到自己的进步，增强他们管理哮喘的积极性。

（4）识别触发因素：PEF变化可以帮助找出导致哮喘加重的环境或活动因素，便于提前应对。

（5）评估治疗效果：新治疗方案后，PEF可以用来判断治疗是否有效。

（6）预防急性发作：通过监测PEF，可以及时发现问题，采取措施减少急性发作的风险。

总之，PEF测量是儿童哮喘管理的重要工具，有助于提高控制水平和生活质量。

**问题17什么是PEF的预测值和最佳值？**

在儿童哮喘的管理中，PEF（峰流速）是一个重要指标。它帮助医生和家属了解孩子的呼吸状况。以下是关于PEF的预测值和最佳值的简明介绍。

（1）PEF的预测值：预测值是根据儿童的年龄、性别和身高计算出的理想峰流速。这是儿童在没有哮喘时可能达到的最佳肺功能水平。

（2）PEF的最佳值：最佳值是指儿童在特定时间内测量到的最高峰流速。这通常是在哮喘控制良好的情况下测得的，代表了孩子的实际肺功能状态。

（3）如何使用PEF值

①监测变化：定期测量PEF，并与预测值和最佳值比较，以观察是否有下降趋势。

②调整治疗：如果PEF值低于最佳值，可能需要调整治疗方案，包括药物和避免诱因。

③应对紧急情况：如果PEF值显著下降（通常低于60%预测值），应及时就医。

总结

了解PEF的预测值和最佳值对于管理儿童哮喘非常重要。家长和患者应定期监测PEF，并与医生合作，及时调整治疗，以保持儿童的健康。

**问题18如何使用PEF仪？**

使用PEF仪是管理儿童哮喘的重要方法。以下是简单的步骤和注意事项：

使用步骤

（1）准备设备：确保PEF仪干净且工作正常。

（2）正确姿势：孩子应站立或坐直，以便更好地呼吸。

（3）深吸气：鼓励孩子深吸一口气，填满肺部。

（4）快速呼气：孩子要用力快速地通过PEF仪呼气，尽量做到一次性呼出。

（5）记录数据：读取并记录仪器显示的峰流速（单位：升/分钟）。建议记录三次，取最高值。

（6）定期监测：每天在早晨和晚上测量，以监控肺功能变化。

（7）与医生沟通：记录结果，定期与医生讨论，以调整治疗方案。

注意事项

（1）咨询医生：开始使用前，请向医生咨询，学习正确的方法。

（2）保持一致：在相同的时间和条件下测量，以获得可靠数据。

（3）了解最佳值：与医生一起确定孩子的最佳峰流速，用于日常监测。

**问题19如何判断PEF仪测定的结果？**

判断儿童哮喘的PEF（峰值呼气流量）结果非常重要，可以按照以下步骤进行：

（1）了解PEF：PEF是呼气时的最大流速，反映气道通畅情况。正常值因年龄、性别和身高而异。

（2）测量准备：确保PEF仪器校准正确，让儿童放松，避免使用影响结果的药物。

（3）进行测量：让儿童坐直，深吸气后快速用力呼气。重复三次，记录最大值。

（4）评估结果

①绿色区（≥80%）：控制良好，症状少。

②黄色区（50%-79%）：可能有气道问题，需注意。

③红色区（<50%）：气道严重受限，应立即使用救援药物并寻求帮助。

（5）记录与分析：定期记录PEF，观察变化。与医生讨论结果，及时调整管理计划。

（6）注意事项

频繁的黄色区或红色区需注意触发因素，并与医疗团队沟通。

**问题20常用的儿童哮喘临床评估工具有哪些？如何选择使用？**

儿童哮喘的临床评估工具对于诊断和管理非常重要。常用的工具包括：

（1）哮喘控制问卷（ACQ）：适用于5岁及以上儿童，评估过去一周的症状和药物使用，判断哮喘控制水平。简单易用，适合日常监测。

（2）儿童哮喘控制测试（ACT）：针对4至11岁儿童，由家长和孩子共同完成。

帮助了解哮喘控制情况。

（3）哮喘严重性评分：评估症状频率、夜间觉醒和急救药物使用，帮助医生制定治疗方案。

（4）峰流速计：测量呼气峰流速，适合5岁及以上儿童，能监测气道情况。

可用于自我监测，发现症状恶化的早期迹象。

（5）哮喘管理计划：结合评估结果，为患者和家属提供应对症状的指导。

个性化管理，有助于患者自我管理。

如何选择工具

选择合适的评估工具时，需要考虑：

（1）年龄：确保选用适合儿童年龄的工具。

（2）症状控制需求：长期监测可用ACQ或ACT，急性症状可重点使用峰流速计。

（3）家庭参与：选择易懂的工具，方便家长参与。

（4）医疗资源：在资源有限的情况下，选择简单的工具。

结论：结合这些工具，可以全面评估儿童哮喘的控制情况。医生应根据儿童的具体情况和家庭需求，灵活选择评估工具，以实现最佳管理效果。

**问题21儿童哮喘如何分期、分级？**

儿童哮喘的分期和分级主要根据症状的频率和严重程度。根据国际哮喘和过敏协会，儿童哮喘可以分为四个阶段：

（1）间歇性哮喘：症状少于每周两次，夜间症状少于每月两次，肺功能正常。

（2）轻度持续性哮喘：症状每周超过两次，夜间症状每月1-2次，肺功能正常。

（3）中度持续性哮喘：症状每天出现，夜间症状每周一次，肺功能轻度下降。

（4）重度持续性哮喘：症状持续，夜间症状频繁，肺功能明显下降。

**问题22儿童哮喘的防治原则是什么？**

儿童哮喘的防治原则包括：

（1）识别和避免诱因

①过敏源：减少接触尘螨、花粉、霉菌和动物皮屑。

②环境因素：避免吸烟、空气污染、冷空气和强烈气味。

③运动：运动前热身，选择适合的运动。

（2）规范用药

①长期控制药物：如吸入性糖皮质激素，帮助控制哮喘。

②急救药物：如短效β2激动剂，缓解急性症状，随身携带。

（3）监测病情

①定期监测肺功能，使用峰流速仪评估状况，记录症状变化。

②教育家长识别早期症状，及时应对。

（4）教育和支持

①教育家庭了解哮喘，包括病因、症状和药物使用。

②定期与医生沟通，调整治疗方案。

（5）制定应急计划

①制定个性化的哮喘管理计划，包括日常管理和急性处理。

②确保家人和学校了解该计划，以便紧急时正确处理。

（6）心理支持

注意孩子的心理健康，必要时提供支持和咨询，帮助他们应对压力。

**问题23儿童哮喘可以治愈吗？**

儿童哮喘是一种常见的慢性病，目前无法完全治愈。尽管有些孩子的症状在青春期可能减轻，但这不代表哮喘消失。

管理方法

（1）长期药物：吸入性糖皮质激素（ICS）可减少气道炎症，控制症状。

（2）急救药物：短效β2-激动剂（SABA）可迅速缓解急性症状。

（3）避免诱因：识别并远离过敏原和刺激物，如烟雾和花粉。

（4）定期检查：孩子需定期就医，监测病情，调整治疗。

（5）教育和自我管理：家长和孩子要学习如何使用药物、识别症状和制定应急计划。

**问题24哮喘控制药物有哪几类？**

哮喘控制药物主要分为两类：长期控制药物和急救药物。

（1）长期控制药物：这些药物帮助减少哮喘症状，通常需要每天服用。

①吸入性糖皮质激素（ICS）：如氟氯噻吨，减轻气道炎症。

②长效β2-激动剂（LABA）：如沙美特罗，通常与ICS一起使用，帮助放松气道。

③白三烯受体拮抗剂：如孟鲁司特，减少气道炎症。

④抗IgE抗体：如奥马珠单抗，适合特定过敏性哮喘。

⑤抗IL-5生物制剂：如美克单抗，针对特定类型哮喘。

（2）急救药物：这些药物用于快速缓解突发的哮喘症状。

①短效β2-激动剂（SABA）：如沙丁胺醇，迅速舒张气道。

使用建议：长期控制药物需遵医嘱使用，定期评估效果。急救药物应随身携带，以应对突发情况。

**问题25哮喘缓解药物有哪几类？**

哮喘缓解药物主要有两类：短效β2-激动剂和抗胆碱药物。这些药物能迅速缓解哮喘症状，尤其是在发作时。

（1）短效β2-激动剂（SABAs）：

①作用：通过放松气道肌肉，快速缓解喘息和呼吸急促。

②常见药物：沙丁胺醇（Albuterol）、特布他林（Terbutaline）。

③使用：通常通过吸入器，效果几分钟内显现，持续约4-6小时。

（2）抗胆碱药物：

①作用：通过抑制气道神经，帮助放松支气管。

②常见药物：异丙托溴铵（Ipratropium bromide）。

③使用：也通过吸入器，主要用于急性发作的辅助治疗。

**问题26 ICS包括哪些药物？适应证是什么？有哪些不良反应？**

（1）常见的ICS药物有：①布地奈德（Budesonide）②氟替卡松（Fluticasone）③氟氯噻噻（Flunisolide）④美氟氯噻（Mometasone）⑤倍氯米松（Beclomethasone）

（2）ICS主要用于：

①长期控制哮喘：帮助减少哮喘发作的频率和严重性。

②减少气道炎症：减轻气道的炎症，提高肺功能。

③预防运动诱发的哮喘：运动前使用可防止运动后出现哮喘症状。

（3）ICS可能引起的副作用包括：

①局部反应：咽喉痛；口腔真菌感染

②全身反应（长期大剂量使用时）：生长速度减慢；骨密度降低；体重增加

③其他：情绪变化（如焦虑、抑郁）；视力问题或白内障风险增加。

**问题27长期ICS治疗对儿童生长发育有无影响？**

长期使用吸入性糖皮质激素（ICS）治疗儿童哮喘对生长的影响是一个重要问题。以下是一些关键点：

（1）生长速度：长期ICS可能会轻微影响儿童的生长速度，尤其是在使用高剂量时。初期可能会看到生长速度下降，但大多数孩子在停药后会恢复正常。
（2）剂量影响：使用低剂量ICS通常不会显著影响生长，而高剂量可能导致更明显的影响。因此，医生会根据孩子的情况选择合适的剂量，以控制哮喘并保护生长。
（3）监测生长：在ICS治疗期间，医生会定期检查孩子的身高和体重，以确保他们正常生长。如果发现问题，医生会调整治疗方案。
（4）治疗建议：许多临床指南建议在哮喘控制良好时使用最低有效剂量的ICS。这有助于减少对生长的影响。家长应与医生合作，确保孩子得到最佳的治疗和生长支持。

**问题28 β2受体激动剂包括哪些药物？适应证是什么？有哪些不良反应？**

β2受体激动剂是常用的哮喘药物，分为短效和长效两类。

（1）β2受体激动剂种类

①短效β2受体激动剂（SABA）

例子：沙丁胺醇（Salbutamol）、特布他林（Terbutaline）。

用途：快速缓解急性哮喘发作，预防运动引发的哮喘。

②长效β2受体激动剂（LABA）

例子：福莫特罗（Formoterol）、沙美特罗（Salmeterol）。

用途：长期控制哮喘症状，通常与吸入性皮质类固醇一起使用。

（2）不良反应

常见的不良反应包括：

①心血管问题：心跳加快、心悸。

②颤抖：手抖或肌肉颤动。

③头痛：部分患者可能会出现。

④低钾血症：长期使用可能导致血钾低。

⑤耐药性：长期使用短效药物可能效果减弱。

（3）管理建议

①监测：定期检查症状和副作用，必要时调整治疗。

②联合使用：建议与吸入性皮质类固醇一起使用LABA，以减少急性发作的风险。

③教育：患者和家属应了解何时使用短效药物，并知道何时寻求帮助。

**问题29抗胆碱能药物包括哪些药物？适应证是什么？有哪些不良反应？**

（1）抗胆碱能药物

抗胆碱能药物用于帮助扩张气道，主要包括：

①阿托品②溴化伊帕特罗③噻托溴铵④乌美溴铵

（2）适应证

这些药物适用于：

①慢性阻塞性肺病（COPD）

②哮喘：尤其在急性发作时。

③急性支气管痉挛

④某些呼吸道疾病，如肺炎合并症状时。

（3）不良反应

使用抗胆碱能药物可能出现的副作用包括：

①口干②喉咙不适③眼部不适（如瞳孔扩大）④心率加快⑤便秘⑥尿潴留

**问题30半胱氨酰LTRA适应证是什么？有哪些不良反应？**

（1）适应证

①支气管哮喘：适用于轻度到中度持续性哮喘，特别是对吸入型糖皮质激素（ICS）反应不佳的儿童。

②运动诱发性哮喘：可以预防运动时的哮喘发作。

③过敏性鼻炎：适合同时有哮喘和过敏性鼻炎的儿童。

④慢性咳嗽：用于因哮喘引起的持续性咳嗽。

（2）不良反应

①常见不良反应：

消化不适：腹痛、恶心、腹泻。

头痛、嗜睡或失眠。

皮肤反应：如皮疹和瘙痒。

②少见但严重的不良反应：

精神变化：情绪波动、焦虑或抑郁。

过敏反应：呼吸困难、喉咙肿胀、荨麻疹。

**问题31哪些哮喘患儿可以使用变应原特异性免疫治疗（allergen specific immune therapy，AIT）？**

（1）适合使用变应原特异性免疫治疗（AIT）的儿童哮喘患者

①过敏原检测：患者需确认对特定过敏原（如花粉、尘螨、动物皮屑等）有过敏反应。

②哮喘类型：适用于过敏性哮喘，特别是症状与特定过敏原相关的儿童。

③控制不佳的哮喘：如果常规药物（如吸入性皮质类固醇）不能有效控制症状，可以考虑AIT。

④年龄要求：通常适用于5岁及以上的儿童。

⑤意愿和遵从性：患者及家属需愿意遵守治疗计划，定期复诊。

（2）不适合使用AIT的儿童

①非过敏性哮喘：如果没有明确的过敏原，AIT通常不适用。

②严重健康问题：有严重心脏疾病或免疫缺陷的患者需要谨慎使用AIT。

③过敏反应史：有严重过敏反应史的儿童在使用AIT前需仔细评估。

**问题32哪些哮喘患儿可以使用单克隆抗体等生物治疗？**

儿童哮喘患者中，可以使用单克隆抗体等生物治疗的主要条件包括：

（1）过敏性哮喘：对于那些因过敏原（如尘螨、花粉、动物皮屑等）引起症状的儿童。

（2）中重度哮喘：症状频繁，使用常规药物（如吸入性糖皮质激素）仍难以控制的患者，尤其是需要口服糖皮质激素的儿童。

（3）生物标志物：如血液中的嗜酸细胞增多或IgE水平升高，表明可能适合使用生物治疗。

（4）伴随过敏疾病：如过敏性鼻炎或特应性皮炎，影响哮喘控制的儿童。

（5）年龄：通常适用于6岁及以上的儿童。

（6）治疗效果不佳：即便按医嘱用药，症状依然严重的患者。

在考虑生物治疗时，医生会根据病史、症状和检查结果进行评估。这种治疗能显著改善一些患者的情况，但也需注意副作用和费用。

**问题33哮喘发作时应首先使用哪些药物？如何选择给药方式？**

在哮喘发作时，首先使用短效β2-肾上腺素激动剂（SABA），如沙丁胺醇（Albuterol）。这些药物能迅速打开气道，缓解症状。

（1）药物选择

①短效β2-肾上腺素激动剂（SABA）：这是首选药物。

②吸入型糖皮质激素（ICS）：在某些情况下，重度发作时可考虑使用。

（2）给药方式

①吸入器（MDI）：最常见的方式，配合空间器使用能更好地将药物送入气道。

②干粉吸入器（DPI）：适合能配合使用的儿童。

③雾化器：适合小儿或重度发作患者，能持续提供药物。

（3）注意事项

①年龄和能力：小孩可能更适合雾化器，而较大儿童可用吸入器或干粉吸入器。

②病情严重程度：如发作严重，需根据医生的建议调整药物剂量。

③监测效果：发作后要观察症状改善情况，如无改善，请及时就医。

**问题34什么是吸入治疗？有何优、缺点？**

吸入治疗是通过呼吸器将药物直接送入肺部的一种方法。它常用于治疗哮喘。药物可以是减轻炎症的药物（如吸入性糖皮质激素）或帮助扩张气道的药物（如支气管扩张剂）。

（1）吸入治疗的优点

①直接作用：药物迅速到达肺部，快速缓解症状。

②副作用少：相对于口服药物，吸入药物的全身吸收较少，副作用较轻。

③快速效果：在急性发作时，支气管扩张剂能快速改善呼吸。

④剂量灵活：医生可以根据需要调整药物剂量。

（2）吸入治疗的缺点

①技术要求：患者需要掌握正确的使用方法，尤其是儿童可能会遇到困难。

②依赖性：需要定期使用，可能会让患者依赖药物。

③局部副作用：长期使用可能导致口腔感染或声音嘶哑。

④经济负担：某些设备和药物可能较贵，对家庭造成压力。

**问题35如何选择吸入装置？**

（1）吸入装置的类型

①喷雾吸入器（MDI）：适合大多数儿童，但需要良好的手眼协调。

②干粉吸入器（DPI）：适合能有效吸气的儿童，但通常不适合5岁以下的孩子。

③雾化器：适用于小孩或重症患者，提供较大剂量的药物。

（2）年龄和能力

①年龄：5岁以下的儿童可用MDI配合空间器或雾化器；6岁及以上的儿童可以试用DPI。

②能力：考虑儿童的手部协调和吸气能力。

（3）使用便利性

①易用性：选择简单的装置，方便家庭使用。

②维护：了解设备的清洁要求，选择容易维护的装置。

（4）药物兼容性

①确保吸入装置与所用药物相匹配。

（5）患者依从性

①教育：为患者和家属提供正确的使用培训。

②个体偏好：考虑儿童的喜好，增强他们的使用积极性。

（6）医生建议

①定期与医生沟通，评估吸入技巧和药物效果，并根据需要调整方案。

**问题36如何正确使用pMDI？**

（1）准备吸入器：

①轻轻摇动吸入器，确保药物混合均匀。

②新吸入器需首次使用时喷出几次。

（2）检查嘴巴：确保嘴巴清洁，最好在使用前刷牙和漱口。

（3）正确姿势：孩子坐直或站直，保持放松。

（4）使用空间舱：如果孩子难以直接吸入，可以使用空间舱（Spacer）来帮助吸入。

（5）吸入步骤：

①将吸入器放在嘴唇之间，形成密封。

②启动吸入器的同时深吸气。保持吸气5-10秒，然后慢慢呼出。

（6）重复使用：如果需要再次吸入，等30秒到1分钟再重复。

（7）清洁吸入器：定期按照说明书清洗吸入器，保持其正常工作。

注意事项

①观察反应：使用后注意孩子的症状变化。如果没有改善或加重，及时就医。

②定期检查：与医生沟通，确认使用方法和治疗效果。

③药物管理：检查药物有效期，及时补充。

**问题37如何正确使用储雾罐？**

（1）准备：

①清洗储雾罐，确保干净。检查药物是否在有效期内。

②新罐使用前，摇匀并喷一次测试。

（2）保持姿势：

①儿童应坐直或站立，以帮助药物顺利进入肺部。

②小孩可在成人帮助下使用。

（3）喷雾器放置：

①摇匀储雾罐，去掉保护盖。

②将喷嘴放在儿童嘴里，或使用面罩。

（4）喷药：

①吸气时按下储雾罐，释放药物。鼓励儿童深吸一口气。

②吸气保持5-10秒，然后慢慢呼气。

（5）等待：如果需要用其他药物，间隔至少30秒再用。

（6）清洁：使用后清洗喷嘴，并盖好保护盖。

（7）观察：注意儿童的呼吸情况，确认药物有效，留意任何副作用。

注意事项

①定期检查：确保储雾罐在有效期内，避免使用过期药物。

②教育和练习：教儿童正确使用储雾罐，帮助他们练习。

③遵医嘱：始终按医生指示使用药物。

**问题38如何正确使用准纳器？**

（1）准备：

①确保准纳器和药物在室温下，摇匀药瓶（如果需要）。

②新罐或长时间未用的罐，喷出1-2次以排气。

（2）位置：

①孩子坐或站立，头稍向后仰。

②将准纳器放在嘴唇间，嘴唇要紧闭。

（3）使用空间器（如有）：

①小孩使用空间器可以提高药物效果。

②确保空间器与准纳器连接好。

（4）吸入药物：

①按下准纳器，让药物喷出，同时让孩子慢慢深吸气。

②吸气时，保持约5秒，之后再呼气。

（5）重复（如需）：如果医生建议多喷，等待约30秒后再重复。

（6）清洁：每周用温水冲洗喷嘴，确保干燥。

注意事项

①观察效果：使用后注意孩子的症状变化，记录副作用。

②定期复查：定期与医生沟通，确认用药效果。

③教育孩子：让孩子了解哮喘管理的重要性，鼓励他们参与治疗。

**问题39如何正确使用都保？**

都保是一种用于儿童哮喘的吸入药物，包含抗炎成分布地奈德和扩张气道的福莫特罗。以下是正确使用都保的步骤和注意事项：

使用步骤：

（1）摇匀：在使用前轻轻摇晃罐子。

（2）准备吸入器：确保吸入器和喷嘴干净，无杂物。

（3）呼气：深吸一口气，排出肺部的空气。

（4）吸入药物：

①将喷嘴放入嘴中，嘴唇紧闭。

②按下喷雾器，深吸药物，尽量将药物吸入。

（5）屏住呼吸：吸入后屏住呼吸5-10秒。

（6）呼气：慢慢呼气。

（7）清洁吸入器：使用后按说明书清洁喷嘴。

注意事项：

①遵循医嘱：按照医生的处方使用，并定期复查。

②监测症状：记录哮喘症状，及时与医生沟通。

③避免误用：都保不适合急救，急性发作时请使用医生开的短效药物。

④了解副作用：注意喉咙痛、声音嘶哑等症状，若有异常应及时就医。

**问题40如何正确使用压缩雾化器？**

（1）准备设备：

①确保雾化器的所有部件干净、无损坏。

②检查药物的有效期，并根据医生的处方准备好药物。

（2）组装雾化器：按照说明书组装雾化器，确保各部分连接牢固。

（3）准备药物：将药物倒入雾化杯中，使用推荐剂量。如需稀释，可加入生理盐水，按医生建议进行。

（4）连接电源：将雾化器插入电源，确保开关关闭。

（5）佩戴面罩或口含器：选择适合儿童的面罩或口含器，确保紧贴面部，避免漏气。

（6）启动雾化器：打开雾化器，观察雾化杯是否产生雾气。儿童应保持安静，深呼吸药物。

（7）雾化过程：雾化通常持续5到15分钟。观察儿童，如感到不适，及时停止使用。

（8）结束后清洁：

①雾化结束后，关闭设备，拆卸各部件。

②按说明书清洗雾化杯和面罩，彻底晾干。

（9）定期检查设备：定期检查雾化器，确保其正常工作。如有问题，及时维修或更换。

注意事项

①使用频率：按医生指导使用，不要自行更改用药。

②监测效果：观察儿童的症状变化，及时与医生沟通。

③心理支持：鼓励儿童放松，可以通过玩具或音乐帮助他们。

**问题41吸入技术对哮喘治疗效果有哪些影响？**

（1）药物效果：吸入治疗是哮喘管理的常用方法，包括吸入性糖皮质激素（ICS）和支气管扩张剂。正确的吸入方法能让药物有效到达气道。

（2）药物吸收：不当的吸入技术会导致药物无法充分吸收，比如吸气过快或过慢。

（3）减少副作用：正确使用吸入器可以减少药物的副作用，提高患者的依从性。

（4）提高依从性：教育儿童及家长正确的吸入技术，可以增强他们的信心，从而更好地遵循治疗方案。

（5）监测效果：通过正确的吸入方法，医生可以更准确地评估药物效果，必要时调整治疗方案。

（6）个体化治疗：不同类型的吸入器适合不同的儿童。选择合适的吸入器并确保正确使用，可以提高治疗效果。

（7）教育和培训：医务人员应定期为患者及家属提供吸入技术培训，使用视频和示范可以提高学习效果。

**问题42常见的吸入技术错误有哪些？**

（1）未摇匀药物：气雾剂（MDI）在使用前需摇匀，未摇匀会导致剂量不准确。

（2）操作不当：儿童可能不知道如何正确使用吸入器，比如未在吸气时按压。

（3）吸气速度不对：使用干粉吸入器（DPI）时，吸气速度如果太慢，药物无法充分进入肺部。

（4）未屏住呼吸：吸入药物后，需屏住呼吸几秒钟，这样药物能更好地沉积在肺部。

（5）清洁不当：吸入器如果不定期清洁，可能影响药物的释放效果。

（6）未按时使用药物：未按照医生建议的时间使用药物，可能导致症状加重。

（7）选择不合适的吸入器：未根据儿童的需要选择合适的吸入器，可能导致药物效果不佳。

（8）缺少帮助：小孩在使用吸入器时，如果没有大人的指导，可能操作不当。

**问题43为什么要对哮喘患儿进行管理？**

（1）减少症状：良好的管理可以减少咳嗽、喘息和呼吸急促，让孩子的日常生活更舒适。

（2）预防急性发作：有效的管理能减少哮喘发作的次数和严重程度，降低急救和住院的需要。

（3）改善肺功能：适当的管理帮助维持孩子的肺部健康，防止长期损伤。

（4）教育与自我管理：管理过程可以让孩子和家长学习如何识别触发因素和正确使用药物，增强孩子的独立性。

（5）心理健康：控制哮喘症状有助于减轻孩子的焦虑和压力，让他们心理更健康。

（6）社会参与：良好的哮喘控制让孩子能参与学校和社交活动，增强自信心。

综上所述，哮喘管理不仅是控制症状，也是提升孩子生活质量的重要措施。通过家庭和医生的共同努力，可以实现更好的治疗效果。

**问题44哮喘患儿为什么要定期到医院复诊？多长时间复诊1次？**

（1）病情监测：定期复诊可以帮助医生了解患儿的哮喘控制情况，如症状的频率和严重程度。这有助于及时调整治疗。

（2）药物管理：医生会根据患儿的情况调整药物种类和剂量，确保治疗有效，避免副作用。

（3）健康教育：复诊时，医生会给家长和孩子提供哮喘管理知识，包括如何识别诱因和正确使用吸入药物。

（4）检查合并症：哮喘患儿可能有其他健康问题，复诊可以帮助医生识别并处理这些问题。

（5）预防发作：定期评估可以降低哮喘急性发作的风险，提高患儿的生活质量。

复诊频率：复诊的时间取决于患儿的病情。一般来说，病情控制良好的孩子每3-6个月复诊一次；病情不稳定的孩子可能需要每月复诊，以便及时调整治疗。

**问题45哮喘患儿复诊的主要内容有哪些？**

（1）病史回顾：询问最近的症状，如咳嗽、喘息、呼吸急促及夜间发作情况。

（2）用药情况评估：检查日常和急救药物的使用，确保患儿正确使用吸入器。

（3）肺功能检测：根据年龄进行肺功能测试，如峰流速测定，评估呼吸情况。

（4）过敏原检测：必要时进行过敏原检测，以找出可能的过敏源。

（5）环境评估：讨论家庭和学校中的哮喘触发因素，如烟雾、宠物和霉菌。

（6）教育与自我管理：

①提供哮喘管理知识，帮助患儿和家长识别症状和应对措施。

②强调制定个人哮喘行动计划。

（7）随访计划：根据病情确定下次复诊的时间，必要时调整治疗方案。

**问题46哮喘的早期预防措施有哪些？**

（1）环境管理

①避免过敏原：减少儿童接触花粉、尘螨、宠物皮屑和霉菌。

②空气质量：保持室内空气新鲜，避免烟草烟雾和污染。使用空气净化器有帮助。

③湿度控制：保持室内湿度在30%-50%，防止霉菌生长。

（2）健康教育

①了解哮喘：教育家长和孩子识别哮喘症状和诱因，学会正确使用吸入器。

②自我监测：定期使用峰流速仪，监测肺功能，及时发现问题。

（3）定期体检

①专业评估：定期看儿科或呼吸科医生，检查哮喘控制情况。

②疫苗接种：按时接种流感疫苗和肺炎疫苗，降低感染风险。

（4）药物管理

①长期控制药物：在医生指导下使用吸入性糖皮质激素等药物。

②急救药物：家庭中备有急救药物（如短效β2激动剂），并了解使用方法。

（5）生活方式调整

①健康饮食：鼓励均衡饮食，增加富含抗氧化物和Omega-3脂肪酸的食物。

②适度锻炼：根据医生建议进行锻炼，增强体质。

（6）心理支持

情绪管理：关注儿童情绪，提供心理支持，帮助缓解压力和焦虑。

**问题47哮喘常见的触发因素有哪些？**

（1）过敏原

①尘螨：小的尘螨生长在床上、地毯和家具上，常常是哮喘的触发因素。

②宠物皮屑：猫、狗等动物的皮屑和毛发也可能引发哮喘。

③花粉：花粉在春秋季节特别多，可能引发过敏性哮喘。

④霉菌：湿气重的地方容易滋生霉菌，霉菌孢子可以刺激呼吸道。

⑤蟑螂和昆虫排泄物：蟑螂等虫类的排泄物也是常见过敏原。

（2）呼吸道病毒：病毒感染，如感冒和流感病毒，会加重哮喘症状，特别是在感冒季节。

（3）环境因素

①空气污染：城市中的空气污染，包括汽车尾气，可能加剧哮喘症状。

②烟雾：香烟烟雾或其他烟雾对呼吸道有刺激作用。

③强烈气味：如香水、清洁剂等化学气味，可能引发哮喘。

（4）气候和温度变化：寒冷空气，冷空气和温度突然变化可能刺激呼吸道，引发哮喘。

（5）运动诱发性哮喘：剧烈运动，特别是在冷空气中，可能导致喘息和呼吸急促。

（6）情绪因素：情绪激动、焦虑或哭泣可能加重哮喘症状。

（7）药物：某些药物如阿司匹林和一些抗炎药，可能使哮喘症状变严重。

（8）食物和饮食

①食物过敏：奶制品、坚果或海鲜等食物可能导致哮喘发作。

②食物添加剂：如某些食品中的化学添加剂，也可能引发哮喘症状。

**问题48常见的过敏原有哪些？**

（1）尘螨：尘螨是哮喘的常见过敏原。它们生活在床垫、枕头、地毯等地方，尘螨的排泄物和尸体会引发过敏反应。

（2）宠物皮屑：宠物的皮屑（特别是猫狗）也是哮喘的常见过敏原。宠物的皮屑随着空气传播，容易被吸入。

（3）花粉：春秋季节，花粉是过敏原之一。树木、草和杂草的花粉会飘散在空气中，引发过敏。

（4）霉菌：霉菌生长在潮湿的地方，如地下室、浴室等。它们的孢子进入空气，也可能引发哮喘。

（5）昆虫过敏原：蟑螂、老鼠和其他昆虫的排泄物或尸体也能引起过敏反应，尤其在家里。

（6）食物过敏：牛奶、鸡蛋、坚果、花生等食物可能导致过敏，并使哮喘症状加重。

（7）烟草烟雾：吸烟或暴露在二手烟中，会使哮喘症状更严重，烟雾中的有害物质刺激气道。

（8）空气污染：汽车尾气和工厂排放的污染物会刺激呼吸道，诱发哮喘。

（9）寒冷空气：冷空气或干冷的天气变化也可能触发哮喘症状。

**问题49哮喘患儿为何需要进行过敏原检测？**

（1）找出过敏源：哮喘的发作常常是由于过敏原引起的，如尘螨、花粉、宠物皮屑和霉菌等。通过过敏原检测，医生可以帮助家长找出孩子可能对哪些物质过敏，从而减少孩子与这些过敏原接触，减少哮喘发作。

（2）制定个性化治疗方案：检测过敏原后，医生可以根据孩子的具体过敏情况，选择合适的治疗方法。例如，针对过敏原进行免疫治疗或调整药物治疗，使得治疗效果更加明显。

（3）提高病情控制：知道了孩子的过敏原后，可以更好地避免这些过敏源，帮助孩子保持病情稳定，减少哮喘急性发作的次数，降低住院的风险。

（4）便于家庭和学校支持：了解孩子的过敏原后，家庭成员和学校老师可以更好地帮助孩子。例如，避免孩子接触宠物、避免在过敏季节外出等措施。这些做法有助于控制哮喘症状，提高孩子的生活质量。

（5）增强家长的管理能力：过敏原检测能帮助家长了解可能引发哮喘的物质，让家长在日常生活中更加注意预防。这也让家长更有信心参与孩子的健康管理。

（6）减少不必要的药物：如果知道孩子的过敏源，医生可以避免使用一些不必要的药物，从而减少治疗中的误用和副作用。

**问题50过敏原检测的主要方法有哪些？**

（1）皮肤点刺试验（SPT）：这种方法通过将不同过敏原液体滴在皮肤上（一般在手臂或背部），然后轻轻刺破皮肤，观察是否有过敏反应。

①优点：操作简单，结果迅速，能检测多种常见过敏原。

②缺点：可能引起局部过敏反应，不适合皮肤敏感的孩子。

（2）血清特异性IgE检测：这项检查通过抽血检测血液中的过敏原特异性免疫球蛋白E（IgE）水平，帮助判断孩子是否对某些物质过敏。

①优点：无需皮肤接触，适合皮肤过敏较重的孩子。

②缺点：结果较慢，有时可能出现假阳性或假阴性。

（3）皮肤划痕试验：这种方法与皮肤点刺试验类似，只是用小刀轻划皮肤后涂上过敏原液，通常用于无法做点刺试验的患者。

①优点：可用于特殊情况。

②缺点：操作相对复杂，可能引起皮肤不适。

（4）鼻拭子过敏原检测：通过收集鼻腔内的分泌物，分析是否有过敏反应。这种方法通常用于过敏性鼻炎，不常用于哮喘。

①优点：无创，适合不愿意做皮肤测试的孩子。

②缺点：对哮喘的诊断效果较差。

（5）食物过敏原检测：如果怀疑某些食物可能导致哮喘症状，医生可能会推荐食物过敏原检测，通常使用点刺试验或IgE检测来判断。

①优点：能帮助发现食物过敏。

②缺点：需要综合多项测试结果来确认。

**问题51什么是螨虫？如何避免尘螨过敏？**

螨虫是一种肉眼看不见的小虫，常生活在床上、枕头、被子、地毯和窗帘等地方。它们喜欢温暖、潮湿的环境。虽然螨虫本身不会引起过敏，但它们的排泄物和死尸里有过敏原，容易引发过敏和哮喘。

如何避免尘螨过敏？

（1）定期清洗床上用品：每周用60°C以上的热水清洗床单、枕套、被罩，能有效杀死尘螨。使用防螨床垫和枕头套也有帮助。

（2）保持室内干燥：尘螨喜欢潮湿，使用除湿机或空调保持室内湿度在40%-50%之间。

（3）减少地毯和软家具：地毯和软家具容易积尘螨，尽量避免使用。如果使用，定期用有HEPA过滤器的吸尘器清理。

（4）使用空气净化器：使用空气净化器可以减少空气中的尘螨和其他过敏原。

（5）清洁积尘区域：定期清洁床头柜、窗帘和书架等地方，减少灰尘积累。

（6）保持儿童活动区域清洁：确保孩子的玩具、衣物、床铺等保持干净，避免带入尘土。

（7）做过敏原测试：如果怀疑尘螨过敏，可以做过敏原测试，并在医生指导下采取防护措施。

**问题52如何避免霉菌过敏？**

霉菌是常见的过敏源，尤其对于有哮喘的儿童，霉菌可能会引发过敏反应，加重哮喘症状。以下是一些有效的预防措施：

（1）保持室内干燥：霉菌喜欢潮湿环境。使用除湿器将室内湿度控制在30%-50%之间，避免潮湿空气滋生霉菌。

（2）定期清洁和通风：定期清洁容易积水的地方，如浴室、厨房和地下室，并保持室内通风。经常打开窗户，帮助减少霉菌生长。

（3）解决漏水问题：检查家中是否有漏水的地方，修理破损的水管和屋顶，防止水渍和湿气积聚。湿润的墙面和地板是霉菌生长的温床。

（4）去除霉菌：如果发现霉菌，可以用漂白水或专用霉菌清洁剂清洗。清洁时戴口罩和手套，避免吸入霉菌孢子。

（5）使用空气净化器：选择带HEPA滤网的空气净化器，有助于清除空气中的霉菌孢子，改善室内空气质量。

（6）避免室外霉菌

湿润的天气和环境容易滋生霉菌。尽量避免让孩子在潮湿的地方玩耍，特别是雨后和湿气较重时。

（7）保持床上用品干燥：床单、枕头和被褥是霉菌的滋生地。定期清洗并保持床上用品干燥，使用透气的床垫保护套，防止霉菌生长。

（8）清洁儿童玩具：定期清洁孩子的玩具和物品，确保它们保持干燥，避免潮湿环境中的霉菌滋生。

**问题53哪些宠物毛屑容易引起过敏？**

宠物毛屑是儿童哮喘过敏的常见诱因。以下是几种容易引发过敏的宠物毛屑：

（1）猫毛屑：猫的毛屑含有一种叫Fel d 1的过敏原，即使猫没有掉毛，这些过敏原也会在空气中传播。
（2）狗毛屑：狗的毛屑和皮肤屑含有Can f 1和Can f 2等过敏原，某些犬种容易产生更多过敏原。
（3）小动物毛屑：兔子、仓鼠和豚鼠等小动物也会释放过敏原，如Lep d 1，容易引发过敏反应。
（4）鸟类羽毛和粪便：鸟的羽屑和粪便含有Gal d 1等过敏原，也能引起过敏症状。

减少过敏反应的方法：

（1）定期清洁家里，减少毛屑堆积。
（2）给宠物洗澡，梳理毛发，减少毛屑。
（3）给宠物设立“禁区”，避免进入儿童卧室。
（4）使用空气净化器，过滤空气中的过敏原。
（5）外出回家后清洁衣物和身体，减少过敏原传播。

**问题54哪些植物与哮喘和过敏性鼻炎有关？**

哮喘和过敏性鼻炎通常与空气中的植物花粉有关。这些花粉会进入体内，引发过敏反应，导致症状加重。以下是一些常见的植物过敏源：

（1）草本植物

①紫花苜蓿（Ryegrass）：它的花粉常在春夏季节引发过敏。

②狗尾草（Timothy grass）：这类草的花粉也会引起哮喘和鼻炎，尤其在春夏。

③雷公藤（Fescue grass）：在湿润地区常见，花粉引发过敏反应。

（2）树木

①白杨树（Birch trees）：白杨树的花粉是春季常见的过敏源。

②橡树（Oak trees）：它的花粉也会引发哮喘和过敏性鼻炎。

③桦树（Willow trees）：这种树的花粉对很多人过敏。

（3）花卉

①菊花（Chrysanthemums）：菊花的花粉常导致过敏症状。

②罂粟（Poppy）：部分罂粟的花粉也会引发过敏。

（4）其他植物

①常春藤（Ivy）：常春藤的花粉可能引起过敏反应。

②仙人掌科植物：某些仙人掌的花粉也能引发过敏。

过敏反应是怎么发生的？

当这些植物的花粉进入呼吸道时，免疫系统错误地把它们当作有害物质。免疫系统反应会释放组胺等物质，导致气道发炎，进而引起哮喘或过敏性鼻炎的症状，如咳嗽、喘息、鼻塞等。

如何减少花粉的影响？

（1）避免接触：尽量避免在花粉高峰期外出，尤其是春秋季节。

（2）清洁室内空气：使用空气净化器，定期清洁家里，减少花粉积聚。

（3）注意个人卫生：外出后换衣服、洗手洗脸，去除身上的花粉。

（4）使用药物：根据医生的建议使用抗过敏药物，如抗组胺药和鼻喷雾剂。

临床注意事项

（1）过敏原检测：通过皮肤测试或血液检查确定孩子的过敏源。

（2）个性化治疗：根据孩子的过敏源和症状制定治疗方案，可能需要药物治疗或免疫治疗。

（3）家长教育：家长应了解过敏症状及其管理方法，确保孩子按时服药。

**问题55如何应对花粉过敏？**

（1）避免接触花粉

①减少外出：花粉浓度高时（早晨和傍晚），尽量让孩子待在室内，避免外出。

②关闭窗户：保持家中窗户关闭，尤其在花粉季节。使用空调时，确保过滤器清洁。

③避开花粉源：避免带孩子去公园或有很多花草的地方。

（2）清洁身体和衣物

①更换衣物：外出回家后，及时更换外衣，避免带回花粉。

②洗脸洗头：花粉可能附着在头发和皮肤上，回家后要及时洗脸和洗头。

（3）使用药物

①抗组胺药：这些药物可缓解鼻塞、打喷嚏等过敏症状。常见的有氯雷他定等。

②鼻喷类固醇：对于鼻塞严重的孩子，可以使用鼻用类固醇喷雾来减轻症状。

③支气管扩张剂：如出现喘息或呼吸困难时，使用支气管扩张剂（如沙丁胺醇）可以缓解症状。

④免疫治疗：对于长期过敏的孩子，医生可能建议通过免疫治疗逐步减少过敏反应。

（4）监控哮喘

①定期监测：使用峰流速计检查孩子的呼吸状况。如果发现下降，及时采取措施。

②遵循治疗计划：按时使用医生开具的预防性药物，如吸入类固醇，减少哮喘发作。

③及时就医：如果哮喘症状加重，及时联系医生。

（5）改善室内空气质量

①使用空气净化器：在家中使用HEPA过滤器的空气净化器，减少空气中的花粉。

②清洁家居：定期清洗床单、窗帘和地毯，减少过敏原。

（6）保持健康饮食

①均衡饮食：多吃水果和蔬菜，增强免疫力，减少过敏反应。

②补充益生菌：益生菌可能有助于调节免疫系统，减少过敏。

（7）了解症状：

观察过敏反应：如果孩子频繁打喷嚏、眼睛痒或呼吸急促，可能是花粉过敏引起的，及时采取措施预防哮喘加重。

**问题56如何应对蟑螂过敏？**

（1）减少蟑螂暴露

①清洁环境：定期打扫厨房、卫生间等地方，清除食物残渣和垃圾。

②封闭食物：把食物存放在密封容器中，避免吸引蟑螂。

③修补裂缝：检查家里的墙壁和地板，封住可能让蟑螂进入的裂缝。

④使用诱杀剂：可选择环保型蟑螂诱杀器，避免使用强毒性化学物质。

（2）避免过敏源接触

①过敏源检测：如果怀疑儿童对蟑螂过敏，可以通过血液或皮肤测试确认。

②减少接触：一旦确认过敏源，尽量避免儿童接触蟑螂及其排泄物。使用空气净化器有助于过滤空气中的过敏源。

（3）药物治疗

①抗组胺药：可以缓解流鼻涕、打喷嚏和眼睛痒等症状。

②鼻用类固醇：可以减轻鼻塞和流涕。

③哮喘药物：如果蟑螂过敏引发哮喘加重，需要根据医生建议使用吸入性激素和支气管扩张剂。

（4）家庭环境管理

①改善空气质量：使用空气净化器，保持通风良好，特别是在潮湿季节。

②控制湿度：保持家中湿度在30%-50%之间，避免潮湿环境吸引蟑螂。

（5）避免二手烟和污染物

①避免吸烟：二手烟会加重过敏和哮喘症状，应避免在家里吸烟。

②减少空气污染：尽量减少家周围的空气污染，尤其是交通和工厂排放物。

（6）寻求专业帮助：如果过敏症状持续，或哮喘控制困难，应尽早去看医生，进行治疗和调整药物方案。

**问题57什么是食物过敏？**

（1）食物过敏的常见症状

①皮肤问题：如红疹、瘙痒或肿胀。

②呼吸问题：如咳嗽、喘息或呼吸困难。

③消化问题：如呕吐、腹痛或腹泻。

④严重反应：如面部肿胀或过敏性休克（可能导致低血压和呼吸困难）。

（2）食物过敏与儿童哮喘的关系：患有哮喘的儿童，特别是那些有食物过敏的孩子，可能会发现食物过敏加重哮喘症状。常见的过敏食物包括牛奶、鸡蛋、坚果、海鲜等。

（3）如何管理食物过敏

①确认过敏源：通过医生的测试确定孩子对哪些食物过敏。

②避免过敏食物：了解并避免孩子食用含有过敏成分的食物，检查食物标签。

③用药治疗：出现过敏反应时，可以使用抗组胺药或肾上腺素注射剂。严重反应时，及时使用肾上腺素并寻求医疗帮助。

④应急准备：家长和孩子要了解过敏症状，并随时携带应急药物。学校和其他看护者也应该知道孩子的过敏情况。

（4）食物过敏的预防

①早期引入食物：研究显示，早期引入某些食物（如花生）可能有助于减少过敏的风险，但需要在医生指导下进行。

②母乳喂养：母乳喂养有助于增强免疫力，并可能降低食物过敏的风险。

**问题58什么是食物不耐受？**

食物不耐受是指吃了某些食物后，身体出现的不适反应。这种反应不是过敏，也不会引发严重的过敏症状。它通常表现为轻微的不适，但可以持续较长时间。

（1）常见的食物不耐受原因包括：

①消化问题：一些食物不能被完全消化（比如乳糖不耐受）。这通常是因为消化系统缺乏足够的酶来分解食物。

②化学物质反应：有些食物中的添加剂、色素或调味料会引起不适。

③天然化学物质：一些食物含有天然的化学物质（如巧克力、奶制品），可能导致不耐受反应。

（2）食物不耐受的常见症状：

①消化问题：如腹痛、腹胀、恶心、呕吐或腹泻。

②皮肤反应：如皮疹或湿疹。

③呼吸问题：如喉咙不适或轻微喘息。

④头痛：有些食物可能引发头痛或偏头痛。

（3）食物不耐受与儿童哮喘的关系

对于哮喘儿童，食物不耐受可能会加重哮喘症状。虽然食物不耐受本身不会引起哮喘发作，但它可能使症状更加严重，尤其是含有防腐剂或过多盐分的食物。

**问题59食物过敏的婴儿如何添加辅食？**

（1）咨询医生：在开始添加辅食前，最好咨询医生，了解哪些食物适合您的宝宝，哪些需要避免。

（2）选择低过敏性食物

①米粉：通常不含过敏成分。

②蔬菜泥：如胡萝卜泥、南瓜泥等。

③水果泥：如苹果泥、梨泥等。

（3）逐一添加新食物：每次只添加一种新食物，添加后观察宝宝的反应3至5天。如果没有过敏症状，可以再添加新的食物。

（4）避免常见过敏原，常见的食物过敏原包括：

①牛奶②鸡蛋③坚果（如花生）④海鲜

这些食物容易引起过敏反应，最好在婴儿有过敏风险时避免。

（5）观察过敏反应

添加新食物后，要注意宝宝是否出现以下症状：

①皮肤：如红疹、瘙痒或肿胀

②消化：如呕吐、腹泻或腹痛

③呼吸：如喘息或呼吸急促

如果宝宝出现这些症状，应停止喂食该食物，并及时就医。

（6）与过敏专家合作：如果宝宝有已知的食物过敏，最好定期去看过敏科医生，进行过敏测试，了解哪些食物是安全的。

**问题60如何管理食物过敏的儿童？**

（1）避免过敏食物：家长需要知道孩子对哪些食物过敏，并确保孩子远离这些食物。每次购买食物时，仔细阅读标签，避免含有过敏源的食品。

（2）药物准备：根据医生的建议，家长应准备常用的药物，如抗过敏药和肾上腺素自动注射器。外出时一定要随身携带，以防过敏反应发生。

（3）教育和培训：家长、老师和孩子的其他照顾者要了解如何应对过敏急症，并学会正确使用肾上腺素自动注射器。

（4）定期检查：定期带孩子去医院复查，了解过敏反应的变化。医生可能会安排过敏测试，帮助了解过敏原。

（5）替代食物：如果孩子不能吃某些食物，家长需要找到营养均衡的替代食品，确保孩子的饮食健康。

（6）通过这些措施，家长可以帮助孩子有效管理食物过敏，避免严重反应，确保孩子的生活更安全、更健康。

**问题61如何诊断食物过敏？**

（1）了解症状和病史

医生首先会询问孩子的症状和病史，了解以下信息：

①症状出现的时间：过敏症状通常在食用某种食物后很快出现，如皮肤发红、呼吸急促、呕吐等。

②家族过敏史：如果家里有过敏性疾病（如哮喘、湿疹等），孩子患过敏的风险会更高。

（2）皮肤点刺试验：皮肤点刺试验是通过在皮肤上轻微刺入食物过敏原，观察是否有红肿反应。它能帮助检测常见食物过敏源，如牛奶、鸡蛋、坚果等。

（3）血液检查：通过抽血检测IgE抗体水平，如果某种食物引起过敏反应，血液中相应的IgE抗体水平通常会升高。这个方法适用于无法做皮肤试验的患者。

（4）食物激发试验：当其他检测结果不明确时，医生可能建议在医院里做食物激发试验。患者在医生监督下食用疑似过敏食物，观察是否出现过敏反应。

（5）排除法：有时，医生会建议暂停食用疑似过敏的食物，观察症状是否减轻。这有助于确认过敏源。

（6）长期管理：诊断后，家长和医生需要制定避免过敏食物的计划，并了解如何使用急救药物，如肾上腺素注射器，防止发生严重过敏反应。

**问题62哮喘患儿能注射疫苗吗？**

是的，哮喘患儿通常可以接种大多数疫苗，这对他们预防疾病非常重要，因为某些感染可能加重哮喘。

以下是几项重要的注意事项：

（1）灭活疫苗：流感疫苗、肺炎疫苗等灭活疫苗对哮喘患儿安全，且建议按时接种，尤其是流感疫苗，因为流感会引发哮喘发作。

（2）减毒活疫苗：麻疹、腮腺炎、风疹等活疫苗一般也适合哮喘患儿。但如果孩子的哮喘控制不佳或在使用免疫抑制药物，接种前应咨询医生。

（3）哮喘发作期：如果孩子正在经历哮喘急性发作或症状不稳定，建议推迟接种，待哮喘控制后再接种。

（4）过敏史：如果孩子对疫苗成分过敏，特别是对鸡蛋过敏（流感疫苗有时含有鸡蛋成分），接种前要和医生确认。

（5）接种后的观察：接种后注意观察孩子的症状，虽然偶尔会有轻微呼吸道反应，但通常是短暂的。

**问题63哮喘发作与上呼吸道感染有什么关系？**

哮喘发作与上呼吸道感染（如感冒）有密切关系，特别是病毒感染常引发哮喘症状加重。以下是一些主要原因：

（1）炎症反应增加：上呼吸道感染会使呼吸道发炎，加重哮喘症状，导致气道收缩和呼吸困难。

（2）气道敏感性增加：病毒感染破坏呼吸道细胞，使气道对过敏原和环境刺激更敏感，更容易发生哮喘发作。

（3）免疫系统反应：感染激活免疫系统，释放一些化学物质，引起气道收缩和粘液增多，造成气道阻塞。

（4）触发过敏反应：病毒感染可能引发过敏性反应，进一步加重哮喘症状。

（5）影响药效：感染期间，气道充血和分泌物增多，影响哮喘药物的吸收效果，可能需要调整治疗方案。

管理建议

为了减少感染诱发的哮喘发作，家长可以：

（1）减少接触传染源：避免与感冒患者接触，特别是病毒传播高峰期。

（2）接种疫苗：按时接种流感疫苗等预防性疫苗。

（3）保持卫生习惯：勤洗手，避免手接触口鼻，减少感染机会。

（4）及时就医：孩子出现感冒且有哮喘病史时，及时就医，防止症状加重。

**问题64哮喘患儿可以运动吗？如何运动？**

哮喘患儿在哮喘控制良好的情况下可以运动。适量运动能增强体质，改善呼吸功能，但家长需要注意运动方式和环境，以防诱发症状。以下是运动建议：

（1）选择适合的运动

①低强度运动：散步、游泳、骑自行车等较轻松的运动更适合哮喘患儿。游泳尤其推荐，因为湿润的空气有助于保护气道。

②间歇性运动：如羽毛球、乒乓球等，可以中途休息，避免气道负担。

③空气质量好时户外运动：尽量选择空气清新、温暖的日子户外活动，避免雾霾和低温天气。

（2）运动前准备

①热身：运动前做10-15分钟轻度热身，让身体逐渐适应运动。

②哮喘药物准备：若医生建议，可以在运动前使用吸入型药物预防哮喘症状。

③避免过敏原：运动环境应避免花粉、尘螨和空气污染等刺激物。

（3）运动中注意事项

①观察症状：家长应观察孩子是否出现喘息、胸闷等症状。

②适时休息：如孩子感到不适，应立即休息或停止运动。

③保持节奏：避免过度激烈的运动，保持平稳的呼吸和适中的强度。

（4）运动后冷身

逐步放松：运动结束后要做5-10分钟的放松活动，帮助身体慢慢恢复。

（5）定期与医生沟通

①定期检查：定期评估哮喘控制情况，医生会根据情况调整运动建议。

②调整运动计划：如症状变化，及时告知医生以调整运动项目。

**问题65运动能诱发哮喘发作吗？**

运动可能诱发哮喘发作，尤其在儿童中。我们称这种情况为运动诱发性哮喘（EIA）或运动诱发支气管痉挛（EIB）。孩子们在运动后可能出现气短、喘息、胸闷等症状。这些症状通常在运动开始几分钟后出现，停止运动后仍会持续一段时间。

为什么运动会诱发哮喘？

运动期间，呼吸速度加快，气道接触到冷、干燥的空气，可能引发气道炎症和收缩，导致哮喘症状。尤其是跑步和游泳等需要长时间呼吸的运动更容易引发症状，而短时间的轻量运动风险较低。

（1）诱发哮喘的常见因素

①运动类型：耐力运动（如跑步）比短时活动更容易引发症状。

②环境因素：冷、干空气或空气污染会加重症状。

③呼吸方式：运动中用嘴呼吸减少了空气的湿润和加温，更易诱发哮喘。

（2）预防和管理方法

家长可以采取以下措施帮助孩子安全运动：

①用药预防：运动前15-30分钟按医嘱使用支气管扩张剂（如沙丁胺醇）能有效预防哮喘发作。

②热身运动：运动前的热身有助于身体适应并减少症状。

③选择适合的运动：温和运动如游泳、步行在温湿度适宜的环境中较安全。

④控制环境：避免在寒冷、干燥或空气污染严重的地方运动。

（3）家长的支持

家长可以通过观察孩子的症状、记录发作情况、按时给药、鼓励适量运动来帮助孩子。运动对健康有益，不应完全避免，但需在安全范围内进行。如果孩子在运动后症状加重，应咨询医生调整治疗方案。

运动诱发性哮喘并不等于不能运动。合理管理和适量运动可以增强肺功能，并提高孩子的健康水平。

**问题66如何预防运动诱发的哮喘发作？**

（1）使用哮喘药物

①运动前用药：在运动前10-15分钟，按医生建议使用哮喘急救药，如沙丁胺醇，以防发作。

②长期用药：如果哮喘反复发作，应和医生讨论是否需要长期控制药物，如吸入类固醇。

（2）选择合适的运动

①低风险运动：游泳、骑车和步行较适合哮喘患者，呼吸负担较小。

②避免剧烈运动：尽量避免长时间跑步或足球，尤其是在冷天或干燥环境下。

（3）热身和冷身

①运动前热身：活动前进行10-15分钟的轻度热身（如慢跑）能帮助身体适应。

②逐渐冷身：运动结束后逐步放松，可以减少突然呼吸变化带来的哮喘风险。

（4）注意周围环境

①避免冷空气和干燥：冷空气和干燥易引发哮喘，可以戴围巾保暖或选择湿润的环境。

②选择空气质量好的时间：空气污染会加重哮喘，建议在空气较好时户外运动。

（5）教育和应急准备

①教育儿童和家长：帮助孩子认识哮喘症状，教会他们自我管理。

②准备应急方案：让教练或老师知情，并随身携带哮喘药物。

（6）定期复查：随着孩子成长，哮喘可能变化，定期复查可确保治疗适当。

**问题67情绪波动能诱发哮喘发作吗？如何预防？**

情绪波动确实可能诱发哮喘。紧张、焦虑、愤怒等情绪会引起身体的应激反应，使气道收缩、呼吸加快，从而加重哮喘症状。儿童的情绪调节能力较弱，情绪波动更容易影响哮喘病情。

（1）预防措施

①情绪管理：教孩子识别和调节情绪。简单的深呼吸或冥想练习可以帮助他们放松，减少情绪对身体的影响。

②家庭支持：家人要关注孩子的情绪变化，保持耐心，营造平和的家庭氛围，避免给孩子过多压力。

③认知训练：通过简单的情绪管理训练，帮助孩子理解情绪和哮喘的关系，学习控制情绪的技巧。

④适度运动：适量的轻度运动可以增强心肺功能，帮助孩子更好地应对情绪波动。但要选择合适的运动强度，避免运动诱发哮喘。

⑤规律生活：保持良好的作息习惯，保证充足睡眠和健康饮食，这样可以减少情绪波动的机会，降低哮喘风险。

（2）药物支持：

对容易因情绪诱发哮喘的孩子，医生可能会建议适量使用短效支气管扩张剂（如沙丁胺醇）作为预防手段。在情绪波动较大时，可在医生指导下提前使用，但要避免依赖药物。

**问题68哮喘对患儿及家长会产生哪些心理行为影响，如何干预？**

1.对儿童的影响

（1）焦虑和害怕：哮喘发作突然，孩子会担心呼吸困难或再次发作，导致焦虑和害怕。

（2）自信心下降：因为哮喘限制了活动，孩子可能会觉得自己不如其他同龄人，产生自卑感。

（3）情绪和学习问题：哮喘症状和治疗可能影响孩子的学习表现和情绪，导致注意力分散和情绪波动。

2. 对家长的影响

（1）焦虑和压力：家长担心孩子的健康，常常感到焦虑和无助，特别是在哮喘发作时。

（2）家庭生活压力：照顾哮喘儿童需要时间和精力，可能让家庭生活变得紧张。

（3）过度保护：有些家长会因为担心哮喘发作而限制孩子的活动，可能不利于孩子的独立性发展。

3.干预措施

以下方法有助于减轻哮喘对心理的负面影响：

（1）心理支持和教育

①心理辅导：提供心理咨询，帮助孩子处理焦虑和恐惧，增强自信。

②家长培训：教育家长如何管理哮喘和支持孩子的情绪，帮助减少他们的焦虑。

（2）哮喘管理知识培训

①药物使用指导：教会孩子和家长如何正确使用药物，提高管理哮喘的信心。

②发作预防教育：教导识别哮喘的早期症状，及时采取措施。

③生活方式建议：提供适合的运动和饮食指导，帮助孩子保持健康的生活方式。

（3）支持小组和活动

①同伴支持：加入哮喘支持小组，与其他家庭分享经验和支持。

②团体活动：参与健康活动，提高孩子的自信心和社交能力。

（4）学校和社区支持

①学校支持：与学校沟通，确保孩子能得到适当的帮助。

②社区资源：利用社区服务和健康组织的支持，为家庭提供更多资源。

**问题69空气污染能诱发哮喘发作吗？如何防护？**

空气污染确实会引发儿童哮喘发作。空气中的污染物，如灰尘、臭氧和有害气体，会刺激呼吸道，引起气道发炎和收缩，加重哮喘症状。这些污染物可能让孩子的咳嗽、气喘和呼吸急促更严重，甚至引发哮喘急性发作。以下是一些有效的防护措施：

（1）减少外出活动：在空气污染严重时，尽量避免孩子在户外活动，尤其是跑步、跳绳等剧烈运动。家长可以查看空气质量指数（AQI），通常AQI超过100时应避免户外活动。

（2）使用空气净化器：在室内使用空气净化器，尤其在睡觉时，可以减少孩子吸入的有害物质，保护呼吸系统。

（3）关闭门窗：空气质量差时，尽量关上门窗，减少污染物进入室内。但也要定期开窗，保持空气流通。

（4）佩戴口罩：如果必须外出，建议孩子戴口罩（如N95），以减少吸入污染物。确保口罩大小合适，不影响呼吸。

（5）关注空气质量预报：家长可以查看空气质量预报，提前安排孩子的活动。空气污染高的天数，尽量安排室内活动。

（6）远离污染源：避免在交通繁忙的地方或高峰时间出行，减少接触尾气等污染物。

（7）咨询医生：在空气污染高的日子，如果孩子的哮喘症状加重，建议与医生沟通，调整哮喘药物。

（8）增强免疫力：合理饮食、足够睡眠和适量运动有助于增强孩子的免疫力，减少空气污染的影响。

**问题70哪些刺激性气味容易诱发哮喘发作？如何预防？**

1.容易引发哮喘的气味

（1）烟雾：香烟、雪茄的烟雾，甚至衣物或家具上残留的烟味都会刺激呼吸道，引发哮喘。

（2）香水和空气清新剂：一些香水和空气清新剂中的化学成分容易让呼吸道不适。

（3）清洁剂和化学品：漂白剂、消毒剂等清洁产品的气味可能会诱发哮喘。

（4）烹饪油烟：尤其是高温煎炸时产生的烟雾含有微小颗粒，会刺激呼吸系统。

（5）油漆和溶剂：油漆、胶水、稀释剂等产品含有挥发性化学物质，易引发哮喘反应。

（6）花粉和植物气味：某些花粉浓度高的植物可能引发过敏和哮喘。

2.预防措施

（1）保持室内通风：在烹饪或使用清洁剂时保持通风，使用排气扇或打开窗户。

（2）避免接触烟草：保持居住环境无烟，避免儿童接触吸烟者的衣物或物品。

（3）减少香水和清新剂使用：少用香水、空气清新剂等产品，选择无香的日常用品。

（4）使用温和清洁产品：避免强刺激成分，选择温和、天然的清洁替代品（如醋或小苏打）。

（5）减少花粉接触：在花粉高发季节减少户外活动，使用空气净化器过滤室内空气。

（6）定期更换过滤设备：空调和空气净化器的滤芯要定期清洁或更换。

**问题71哪些气候变化容易诱发哮喘发作？如何预防**？

1.哪些气候变化容易诱发哮喘？

（1）季节变化和温度波动：春秋两季温差大，花粉和过敏原增多，容易引发哮喘。

（2）湿度的快速变化：湿度过高（如在夏季和雨季）或过低（如冬季）会刺激气道，增加哮喘风险。

（3）空气污染增加：热天气和空气污染会提高颗粒物和臭氧的浓度，特别是在城市中，对儿童哮喘患者尤其不利。

（4）冷空气刺激：冬季的冷空气可能收缩气道，引发哮喘症状。

2.如何预防哮喘发作？

（1）关注天气：在温差大或湿度变化明显的日子，尽量减少户外活动。

（2）保持室内清洁：定期清洁，减少尘螨和霉菌，必要时使用空气净化器。

（3）避免高污染天气出行：雾霾天减少外出，确保室内空气清洁。

（4）寒冷天气防护：出门时让孩子戴口罩和围巾，避免冷空气直接刺激气道。

（5）药物管理：哮喘儿童需在医生指导下合理用药，随身携带急救药物，特别是气候剧烈变化时。

**问题72香烟暴露对哮喘患儿有什么不良影响？如何预防？**

香烟暴露会对哮喘儿童造成严重的健康影响。接触香烟烟雾（二手烟）会使哮喘症状加重，增加急性发作的次数，并可能损伤肺部发育。香烟烟雾中的化学物质会刺激气道，使患儿更容易出现喘息、咳嗽和呼吸困难。长期暴露还会导致肺功能下降，甚至增加药物依赖。

1.香烟暴露对哮喘患儿的主要影响

（1）增加哮喘发作：香烟烟雾刺激气道炎症，使哮喘发作更频繁。

（2）加重症状：烟雾会引起气道黏膜反应，导致喘息、咳嗽等症状加剧。

（3）影响肺功能：长期暴露可能阻碍肺部正常发育，影响孩子未来的呼吸健康。

（4）增加药物需求：需要更高剂量药物控制哮喘，增加副作用风险。

2.如何预防香烟暴露

（1）家庭禁烟：家人避免在家中和车内吸烟，防止孩子接触二手烟。

（2）远离烟雾：带孩子外出时，避开吸烟者或允许吸烟的场所。

（3）告知亲友：提醒亲友在孩子周围避免吸烟，可在户外指定吸烟区。

（4）支持无烟环境：鼓励学校和社区推广无烟环境，减少暴露机会。

（5）帮助家人戒烟：如果家庭成员吸烟，建议寻求戒烟帮助，以减少孩子的健康风险。

**问题73哮喘与肥胖有关吗？如何预防？**

1.哮喘与肥胖的关系

哮喘和肥胖确实有一定关系。肥胖会增加患哮喘的风险，并可能加重症状。肥胖导致体内慢性炎症，影响肺部功能，使呼吸更困难。肥胖儿童的气道反应更敏感，容易引发哮喘。

2.预防措施

（1）健康饮食：为孩子提供营养均衡的饮食，避免高糖、高脂肪的食物。多吃水果、蔬菜、全谷物，有助于维持健康体重。

（2）适量运动：定期运动可以控制体重，增强心肺功能。游泳、步行等轻度活动适合哮喘儿童，有助于提高耐力。

（3）体重控制：保持适合的体重范围。超重儿童可在医生和营养师的指导下进行健康减重。

（4）减少过敏原：避免灰尘、花粉、宠物毛发等过敏源。远离烟雾和污染，减少对呼吸道的刺激。

（5）定期检查：哮喘高风险儿童需定期监测肺功能、体重等。通过随访，及时调整健康方案。

（6）心理支持：肥胖和哮喘可能带来心理压力。家长应关注孩子的情绪，给予支持，必要时寻求心理帮助。

**问题74外出活动或旅游时，有哪些因素容易导致哮喘急性发作？**

（1）过敏原：不同地区有不同的过敏原，如花粉、尘螨、霉菌等。特别是在春秋季节或高花粉地区，这些物质容易诱发哮喘。

（2）空气污染：空气质量差的地方，如大城市或工业区，污染物会刺激呼吸道，增加哮喘风险。家长可以提前查看目的地的空气质量指数（AQI）。

（3）气候变化：冷空气或湿度较高的环境可能刺激气道，导致哮喘发作。潮湿地区霉菌增多也会增加风险。

（4）活动量：户外活动过多，尤其是在寒冷空气中，可能导致气道收缩，诱发哮喘。建议适量活动，避免剧烈运动。

（5）情绪紧张：旅行中的紧张、兴奋或焦虑可能使敏感的孩子更容易发作。帮助孩子保持轻松的心情，减少应激。

（6）食物和药物过敏：接触新的食物或药物可能导致过敏反应。出行时要小心选择食品，并带好抗过敏药物。

（7）空气干燥：冬季或空调房间的干燥空气可能引起气道干涩，导致哮喘。保持适当湿度有助于预防。

建议：

（1）了解目的地的环境，随身带好药物。

（2）避免在高污染或温差大的地方做剧烈活动。

（3）注意饮食，保持情绪稳定。

**问题75哮喘急性发作或加重有时间特点吗？**

（1）夜间和清晨：哮喘症状常在夜里或清晨加重。夜间，体内激素水平较低，气道更敏感，容易引起呼吸困难。这就是很多哮喘患者夜间症状更重的原因。

（2）季节变化：春季和秋季是哮喘发作的高峰期，因为空气中的花粉或霉菌增多，可能引发过敏，从而导致哮喘症状恶化。对过敏性哮喘的孩子，这些季节尤其要注意防护。

（3）空气污染和气温变化：空气污染严重的日子，或气温急剧变化时，哮喘更易发作。特别是在秋冬季节，冷空气会刺激气道，引发急性发作。家长应在污染严重的日子减少孩子户外活动，或出门时给孩子戴口罩。

（4）运动后：剧烈运动或户外活动后也容易触发哮喘。这种“运动诱发型哮喘”在儿童中较常见，家长可在运动前使用缓解药物或随身携带急救药物。

**问题76哪些表现可以预示儿童哮喘的急性发作？**

（1）呼吸急促或困难：孩子在平静状态下呼吸变快或变得困难，尤其是在夜间或清晨时。

（2）频繁咳嗽：特别是夜间、清晨或运动后出现的持续咳嗽，可能表明哮喘加重。

（3）胸闷或胸痛：孩子可能抱怨胸口紧，感觉“喘不过气”。

（4）喘息音：呼吸时发出高音的“哨声”，特别是呼气时，更可能是急性发作的信号。

（5）疲倦或食欲下降：哮喘急性发作会影响身体供氧，孩子可能表现出异常的疲惫或食欲减退。

（6）活动减少：如果孩子因为喘不过气而不愿跑跳或游戏，家长要警惕哮喘可能在加重。

（7）情绪波动：一些孩子在发作前会变得焦虑或易怒，可能因为不适或缺氧。

这些症状并不一定全部出现，但其中几项的突然出现或加重，可能提示哮喘急性发作。家长一旦发现这些变化，应考虑使用急救吸入剂或立即就医。

**问题77如何判断儿童哮喘急性发作的严重程度？**

（1）呼吸频率和困难程度

①轻度：呼吸稍快，孩子没有明显的喘息和困难。

②中度：呼吸明显增快，有轻微的喘息，孩子讲话会停顿。

③重度：呼吸急促，呼吸困难，讲话或哭泣时断断续续甚至难以完成。

（2）喘息声音（哮鸣音）

①轻度：仅在呼气时有轻微喘息声。

②中度：吸气和呼气时都有喘息声。

③重度：喘息声很明显，即使静止也能听见，或逐渐减弱（可能因气道严重阻塞）。

（3）辅助呼吸肌肉的使用

①轻度：无辅助呼吸肌肉使用。

②中度：使用胸肌或腹肌辅助呼吸，吸气时胸廓有轻微下陷。

③重度：明显使用辅助肌肉，呼吸节律不均匀。

（4）血氧水平

①轻度：血氧水平（SpO₂）高于95%。

②中度：血氧水平在92%-95%之间。

③重度：血氧低于92%，应立即就医。

（5）其他症状

①轻度：轻微咳嗽，日常活动不受影响。

②中度：咳嗽加剧，胸闷，活动受限，孩子可能焦虑。

③重度：严重胸闷，难以说话或哭泣，可能出现嘴唇或指甲发紫。

（6）哮喘控制测试或峰流速计（如有）

①轻度：峰流速下降不超过20%。

②中度：峰流速下降20%-50%。

③重度：峰流速下降超过50%。

紧急处理建议

①轻度：可用缓解药（如短效β2受体激动剂），观察症状是否缓解。

②中度和重度：立即用缓解药，并尽快就医。

③极重度（如呼吸停止或意识模糊）：立刻拨打急救电话，进行急救。

**问题78因急性发作住院治疗的哮喘患儿，其出院后的注意事项有哪些？**

（1）按时用药

①遵医嘱使用药物，特别是维持用药，如吸入性糖皮质激素，每天按时使用。

②随身携带急救药物（如短效β2受体激动剂）以应对突发症状，并确保家属和患儿都熟悉药物的正确使用方法。

（2）定期复诊

①出院后建议1-2周内初次复诊，之后每3-6个月复查，以评估病情、调整用药。

②复诊时，医生可能会进行肺功能测试，检查治疗效果。

（3）监测症状和避免诱因

①家属要留意患儿是否有咳嗽、喘息、胸闷等症状，并记录变化情况。

②避免触发因素，如花粉、灰尘、二手烟和宠物毛发；保持室内清洁，防止霉菌滋生。

（4）培养健康生活习惯

①饮食：饮食均衡，避免刺激性食物，如海鲜、坚果等。

②睡眠和运动：保持规律作息，适当运动增强体质，但要避免剧烈运动，以防诱发哮喘。

（5）加强家庭健康教育

①了解哮喘的基本知识，熟悉药物使用和急性发作时的处理方法。

②制定家庭应急预案，以应对突发情况。

（6）注意季节变化：冷天或过敏季节注意防寒保暖，防止呼吸道感染，必要时提前进行抗过敏治疗，降低哮喘发作风险。

**问题79如何判断与识别处于哮喘行动计划绿区的哮喘患儿？**

（1）症状控制情况

绿区代表哮喘症状得到良好控制，包括：

①没有呼吸急促：日常活动中，孩子没有呼吸困难。

②无咳嗽、喘息或胸闷：尤其在早晨和晚上，这些症状很少或没有。

③能正常活动：孩子可以运动或玩耍，不会出现哮喘症状。

（2）峰流速监测：使用峰流速仪测量孩子的呼吸能力。绿区的峰流速一般应达到个人最佳值的80%或以上。如无家庭设备，可在医生处定期检测。

（3）不依赖急救药物：在绿区，孩子一般不需要频繁使用快速缓解药物（如沙丁胺醇）。如果每周使用急救药物不超过一次，且无症状突发，可能在绿区。

（4）夜间睡眠正常：夜间哮喘症状少见，孩子能安稳入睡，不会因咳嗽或胸闷醒来。如果夜间睡眠良好，孩子可能处于绿区。

（5）日常观察：家长应关注孩子的日常表现，记录症状并定期与医生沟通。在绿区，孩子应能正常生活，参与户外活动而不出现症状。

**问题80对处于哮喘行动计划绿区的哮喘患儿，为何仍需制定长期控制治疗方案？**

（1）预防症状复发：哮喘是一种长期的炎症性疾病，即使症状得到控制，气道仍可能有轻微炎症。持续治疗能帮助维持稳定，减少症状复发。

（2）保护气道健康：反复的炎症会使气道变得敏感，甚至受损。长期控制治疗可以减少炎症，保护气道不受长期损害，帮助患儿呼吸更顺畅。

（3）减少急救药的使用：控制治疗能降低急性发作的风险，从而减少“急救”药物的使用频率。这样可以降低因哮喘突然发作而带来的不便和压力。

（4）提高生活质量：有效的长期控制帮助儿童自由参加活动，减少因哮喘带来的生活限制，让他们享有正常的生活和运动。

（5）培养疾病管理意识：长期治疗计划能帮助家长和孩子更好地理解和应对哮喘。当症状轻微时仍保持治疗，可以增强自我管理的信心和能力。

**问题81对处于哮喘行动计划绿区的哮喘患儿，如何降级治疗？**

对于处于哮喘行动计划绿区的儿童（即症状控制良好，能正常活动、睡眠，没有哮喘症状）的降级治疗，可以逐步减少药物剂量，前提是至少3至6个月内病情稳定。降级步骤包括：

（1）确认病情稳定：确保孩子症状稳定，且近期无发作，方可考虑降级。

（2）逐步减少药物：在医生指导下，逐步降低吸入性激素剂量。如果已在低剂量，医生可能会建议停用长效药物，保留低剂量吸入性激素。最终，可能只在需要时用短效缓解药（如沙丁胺醇）。

（3）密切观察：降级后，家长需留意孩子的任何症状变化，如咳嗽或胸闷。如果症状复发，应立即联系医生，调整治疗。

（4）家庭教育：家长要学习哮喘管理的基础知识，了解应急处理，确保能及时识别症状变化。继续定期随访，保持病情监控。

**问题82如何管理哮喘患儿，使其长期稳定在绿区？**

（1）制定哮喘管理计划

①个性化方案：医生会根据患儿的情况制定管理计划，包括每天的控制药物和应急药物，帮助缓解气道炎症并及时应对发作。

②药物剂量和时间表：遵医嘱使用药物，控制在最低有效剂量，减少长期副作用。

（2）定期监测与复查

①定期复诊：每隔1到3个月复诊，检查患儿的肺功能和症状控制情况。

②日常监测：家长可以用峰流速计（PEF）检测患儿的呼吸情况，发现异常，早做应对。

③记录症状：记录哮喘发作的频率和严重度，有助于医生调整治疗。

（3）识别和避免触发因素

①过敏原：减少接触如尘螨、花粉等常见过敏原，保持室内清洁。

②空气污染：避免空气差的天气外出，不使用刺激性气味产品。

③预防感染：避免感冒等感染，及时接种流感疫苗。

④管理情绪：避免焦虑等情绪波动，以减少哮喘诱发。

（4）提高用药依从性

①用药教育：教患儿和家长正确使用药物和吸入装置，确保药效。

②家庭支持：家长应督促患儿按时用药，并提供情感支持，帮助他们建立健康习惯。

③学校沟通：向老师说明患儿病情，让他们在学校也得到适当照顾。

（5）设立应急预案

①急性发作应对：家长需在哮喘发作时及时使用急救药物，必要时拨打急救电话。

②早期识别症状：教患儿辨别发作的早期信号，及时采取措施。

**问题83如何判断与识别处于哮喘行动计划黄区的哮喘患儿？**

在哮喘管理中，哮喘行动计划分为绿区、黄区和红区。黄区意味着哮喘症状加重但不至于紧急。识别哮喘黄区的主要方法包括：

（1）观察症状

①轻中度症状：患儿可能频繁咳嗽、轻微喘息或胸闷。日常活动可能变得困难，尤其是在运动时。

②夜间症状：夜间咳嗽或气促可能影响患儿的睡眠。

（2）使用峰值流量计

峰值呼气流量：如果患儿的呼气流量在个人最佳的50%-80%之间，则可能处于黄区。这可以通过峰值流量计测量。

（3）支气管扩张剂的使用频率

药物需求：如果患儿需要比平时更多的短效支气管扩张剂（如沙丁胺醇）来缓解症状，说明哮喘控制不稳定，可能进入黄区。

（4）活动耐力

体力下降：如果患儿在日常活动中更易疲劳或喘息，表明哮喘可能处于黄区。

黄区应对方法

①增加药物使用：按医生建议增加短效支气管扩张剂的使用。

②观察病情：密切观察症状、峰值流量和药物效果。如果症状在24小时内没有缓解，应及时就医。

**问题84如何应用PEF监测处于黄区的哮喘患儿？**

（1）黄区定义和PEF作用

①黄区指患儿的PEF值在最佳值的50-80%之间，表示症状加重但未到严重发作。

②使用PEF监测可以帮助家长和医生追踪病情变化，及时调整治疗。

（2）PEF监测步骤

①测量频率：早晚各一次，记录每次PEF值，了解呼吸情况的变化。

②记录数据：记录PEF值、时间和症状，方便医生后续了解病情。

③注意环境：测量时保持安静，保证准确性。

④对比症状：观察PEF数据和症状变化，评估咳嗽、气促等是否加重。

（3）调整措施

①药物调整：根据医生建议，可增加缓解药物剂量，如短效β2激动剂，帮助症状缓解。

②避免诱因：避免接触如过敏原、烟雾、冷空气等容易诱发哮喘的因素。

③病情观察：若PEF值持续低于80%，应联系医生。

（4）警惕加重信号：如果PEF值接近50%或症状突然恶化（如持续咳嗽、呼吸急促），请尽快就医。

（5）教育患儿和家长

①正确使用PEF仪器：教会家长和孩子如何正确测量。

②行动计划：家长需了解医生提供的哮喘行动计划，及时采取措施。

**问题85处于黄区的哮喘患儿应首先使用哪些药物？如何使用？**

处于黄区的哮喘患儿症状较轻微，但有恶化的风险。此时需要适当用药以防止病情加重，建议以下用药：

（1）快速缓解药物（SABA）

①推荐药物：沙丁胺醇（如万托林）或特布他林喷雾。

②用法：使用储雾罐，将药物喷入孩子的嘴里，每次1-2喷，4-6小时用一次，直到症状缓解。

（2）吸入性激素（ICS）增量

①推荐药物：如果孩子平时已在用低剂量吸入性激素（如布地奈德、氟替卡松），在黄区状态下可短期增加剂量，比如布地奈德的剂量增加到平时的两倍，用7-10天。

②用法：和日常使用方法一样，确保药物吸入到位。

（3）口服激素

如果快速缓解药和吸入激素增量后症状仍未缓解，医生可能会建议短期使用口服激素（如泼尼松），但需遵医嘱，不建议自行使用。

使用提示

①父母需熟悉喷雾器、储雾罐的正确使用方法。

②观察孩子的症状缓解情况，如呼吸是否顺畅，胸闷是否改善。

③如果症状没有缓解或有恶化迹象，请立即联系医生或就诊。

**问题86如何判断黄区患儿的病情得到有效缓解？**

（1）症状减轻：咳嗽、喘息和胸闷等症状逐渐减少，尤其是夜间症状不再频繁。

（2）肺功能恢复：通过医生指导使用峰值流速仪。如果峰值呼气流速接近最佳值的80%或更高，说明肺功能有改善。

（3）减少用药需求：如果患儿减少了短效缓解药的使用，仅靠日常维持药物即可控制症状，则表示病情在好转。

（4）活动恢复正常：患儿可以正常活动，不会因为哮喘症状而中断或减少活动量。

（5）医生复诊确认：定期到医院复查，医生的评估可以帮助判断病情是否恢复到“绿区”状态。

**问题87黄区的患儿在什么情况下必须立即去医院就诊？**

（1）药物无效：使用短效支气管扩张剂（如沙丁胺醇）后，症状未缓解，表示气道仍然狭窄。

（2）症状加重：按医嘱用药后，咳嗽、喘息或呼吸急促症状继续加重，可能有更严重的发作风险。

（3）呼吸困难：患儿呼吸频率明显加快，伴随吸气时胸部明显凹陷，表明可能缺氧。

（4）口唇或指端发紫：这些症状提示氧气不足，需紧急处理。

（5）无法正常饮食：患儿因喘息或呼吸困难不能进食或喝水，可能导致脱水或营养不足。

（6）神志不清或过度困倦：如果患儿变得特别焦虑或反应迟钝，可能是缺氧或严重哮喘的表现。

（7）夜间症状明显加重：若夜间症状严重到影响睡眠，表明哮喘不稳定。

**问题88对于处在哮喘行动计划黄区的哮喘患儿，如何升级治疗？**

（1）确认哮喘症状

①识别症状：黄区症状包括咳嗽、喘息、呼吸困难或胸闷加重。症状可能无法通过日常药物控制。

②测量峰流速：如果孩子可以配合，使用峰流速仪测量。如果数值在最佳水平的50%-80%，说明需要加强治疗。

（2）增加快速缓解药物的使用

①增加剂量或频率：在医生指导下，增加短效β2激动剂（如沙丁胺醇）的使用，通常每4小时一次，但不要超过1-2天。

②观察效果：若药物能缓解症状，则可继续观察；若无效或症状加重，应尽快联系医生。

（3）考虑抗炎药物的调整

①增加吸入激素剂量：如症状持续，医生可能建议短期增加吸入激素剂量，或短时间使用口服激素。

②避免长期使用高剂量：长期高剂量可能带来副作用，必须在医生指导下使用。

（4）联系医生

①及时咨询：症状未改善或恶化时，家属应联系医生，可能需要进一步治疗甚至住院。

②调整行动计划：在医生帮助下，及时更新哮喘行动计划以适应孩子当前的症状。

（5）家属教育与支持

①了解哮喘管理方法：学习如何正确使用药物，识别症状恶化，便于及时处理。

②提供情感支持：哮喘加重可能让孩子焦虑，家属的支持可以提高他们的依从性和安全感。

（6）后续复查

①复查评估：症状缓解后，需复查药物剂量，看是否能恢复至日常剂量或进一步调整长期治疗。

②记录症状：记录孩子的症状和药物使用情况，以帮助医生调整治疗方案。

**问题89如何避免过度使用速效β_2_受体激动剂？**

（1）明确药物作用：速效β2受体激动剂能迅速缓解哮喘急性症状，但并不能长期控制哮喘。家属需要了解它的作用是短期缓解，而不是根治哮喘。如果使用过多，可能说明哮喘控制不佳。

（2）遵守哮喘管理计划：在医生帮助下制定哮喘管理计划。计划中会规定控制药物和速效药物的使用方法。按计划使用药物，遇到症状变化及时与医生联系。

（3）使用长期控制药物：按时使用医生开的长期控制药物（如吸入性糖皮质激素）能减少急性发作的风险。这样可以少用速效药，控制哮喘的整体状况。

（4）记录症状和用药情况：用哮喘日记记录每天的症状和用药次数。若速效药每周使用超过2次，说明哮喘未被有效控制，需要联系医生调整方案。

（5）避免诱发因素：避免哮喘诱发因素如尘螨、宠物皮屑、空气污染等。减少接触这些因素，有助于控制哮喘，减少急性发作。

（6）学习正确的吸入技巧：确保患儿掌握正确的吸入方法，定期检查吸入技巧是否正确。这样可以让药物发挥最佳效果。

（7）定期随访：定期复诊，让医生检查哮喘控制情况，调整药物。通过肺功能测试等检查，可以更好地监测哮喘病情，减少速效药的使用。

**问题90如何判断与识别处于哮喘行动计划红区的哮喘患儿？**

（1）呼吸困难

红区的哮喘患儿会出现严重的呼吸困难，即使在休息时也不易缓解。典型表现包括：

①呼吸急促且费力，胸部或肋骨间凹陷明显。

②患儿说话、进食或活动时呼吸更为困难。

（2）喘息声变重：严重的喘息声是哮喘加重的信号。红区患儿的喘息声通常在整个肺部都能听到，且急救药物效果不明显。

（3）皮肤或口唇发紫：缺氧的表现之一是嘴唇、指尖和脸色发紫或苍白，这表明哮喘已到危急阶段，需要立即干预。

（4）吸入药无效：如果使用短效的哮喘缓解药（如沙丁胺醇）后，症状没有好转或继续恶化，这可能意味着进入了红区。

（5）其他症状

其他需要注意的症状包括：

①胸部疼痛或压迫感

②情绪异常，如极度疲惫或烦躁

③无法进行日常活动

**应对措施**

如果孩子符合上述多个症状，应：

（1）立即使用急救吸入药，并观察症状变化。

（2）若症状未缓解或恶化，应立刻求医或去急诊。

**哮喘红区的定义**

红区代表哮喘严重发作，需要紧急处理。家长应确保孩子随时有急救药物，并遵循医生建议。

**问题91如何避免哮喘急性严重发作？**

（1）按时服药：遵循医生的用药方案，按时按量服药，避免擅自停药或更改剂量。这可以帮助长期控制哮喘。

（2）避免诱发因素

尽量避免接触会引起哮喘的东西，比如：

①过敏源：如尘螨、花粉、宠物毛发和霉菌。

②空气污染：减少暴露在空气污染严重的地方，外出时可戴口罩。

③烟雾和刺激气体：避免二手烟、香烟和其他刺激物。

④感染：避免病毒感染（如感冒），保持卫生。

（3）定期检测呼吸

使用峰流速仪定期检测孩子的呼吸情况。峰流速值降低时，可能是哮喘恶化的信号，便于提前采取措施。

（4）识别早期症状：学会识别哮喘加重的早期信号，如咳嗽、气短或胸闷，尤其是夜间症状。早期用药可以阻止病情恶化。

（5）遵循哮喘管理计划：医生会提供个性化的哮喘管理计划，包括日常用药和急性发作时的处理方法。家长应熟悉计划内容，并在发作时严格执行。

（6）接种疫苗：按时接种流感疫苗和肺炎疫苗，这可以减少呼吸道感染的机会，从而降低哮喘发作风险。

（7）定期复查:即使症状控制得很好，也应定期复查，医生会根据复查情况调整用药。

（8）学会用急救药物：家长和孩子应熟悉急救药物的使用方法，比如吸入器或雾化器，并随身携带，以备急需。

**问题92哮喘患儿家中或身边应常备哪些急救药物？**

（1）短效β₂激动剂 (SABA) 气雾剂：如沙丁胺醇（Ventolin）或特布他林（Bricanyl）。这是哮喘发作时的首选药物，可快速放松气道，帮助患儿更容易呼吸。建议小儿童使用时搭配储雾罐（spacer），提高药效。

（2）口服或注射类固醇药物：如泼尼松（Prednisone）或地塞米松（Dexamethasone）。在哮喘严重发作时，这些药物可缓解气道炎症，但只在医生指导下短期使用。

（3）日常控制药物的备用：如吸入性糖皮质激素。若哮喘加重，发作期间应继续每日使用控制药，并配合短效气雾剂。注意不要随意增减剂量。

（4）抗过敏药（视情况备用）：若患儿有过敏性哮喘，可备用抗组胺药，但这些药不能用于哮喘急救，仅能辅助缓解过敏症状。

注意事项

（1）检查药物有效期，尤其是急救药，确保都在有效期内。

（2）急救方案和用药指南：家长应学习正确使用气雾剂的方法，与医生一起制定哮喘急救方案，了解何时用药、何时寻求帮助。

（3）定期复查：哮喘状况会随时间变化，需定期复诊，调整药物和治疗方案，确保有效控制病情。

**问题93如何对处于红区的哮喘患儿进行家庭急救？**

（1）立刻使用急救药物

①使用快速缓解药（如沙丁胺醇气雾剂）：每20分钟使用一次，最多3次。此药可以帮助缓解气道收缩，让孩子更容易呼吸。

②正确使用吸入装置：建议使用带面罩的储雾罐来帮助药物更好地进入孩子的气道。

（2）观察孩子的症状：如果药物使用后，孩子的症状没有好转，或出现呼吸急促、嘴唇发青等严重症状，请立刻前往急诊。家属需密切观察孩子的反应，以确保情况不会恶化。

（3）保持冷静，帮助孩子放松：哮喘发作可能让孩子紧张，进而加重症状。家属应保持冷静，并引导孩子放松，尽量深呼吸，帮助减轻焦虑。

（4）让孩子坐直，保持气道通畅：可以让孩子坐在椅子上，身体稍微前倾。避免平躺，因为可能会让呼吸更困难。

（5）避免剧烈活动：在急性发作时，确保孩子休息，不要进行任何剧烈活动，以防加重症状。

（6）如有需要，迅速求助：若急救药物无效或症状加重，必须立即拨打急救电话。等候时，可以按医嘱继续使用急救药。

**问题94红区哮喘患儿急诊就医过程中有哪些注意事项？**

（1）尽快就医，保持冷静

①立刻前往急诊：症状严重、呼吸困难时要立即去急诊，不要耽搁。

②保持冷静：家长情绪稳定可以让孩子更放松，避免加重症状。

（2）使用急救药物

①速效药物：在医生指导下，可用速效支气管扩张剂（如沙丁胺醇）减轻症状。

②正确吸入：确保药物正确吸入，提升效果。

（3）记录症状变化

①观察呼吸：注意孩子呼吸频率和胸部起伏的变化，为医生提供详细信息。

②记录用药：记下药物使用的时间和效果，方便医生判断。

（4）确保空气流通

①保持通风：在安全情况下增加新鲜空气，有助于呼吸。

②远离刺激物：避免烟雾、强烈气味和其他可能诱发哮喘的因素。

（5）携带病历和药物清单

①随身带病历：带上孩子的病历和药物清单，以帮助医生了解病史。

②备用药物：随时备好急救药物，以备不时之需。

（6）避免饮食

不吃东西：急性发作时尽量避免进食，防止呛咳加重。

（7）告知过敏史

及时告知医生：让医生知道孩子的过敏情况，避免误用药物。

（8）与医生保持沟通

①清楚描述病情：简单清晰地告诉医生孩子的症状变化和用药效果。

②遵医嘱：按医生建议进行治疗，不自行更改药物剂量。

（9）急诊后观察并复查

①持续观察：急诊后，家长要继续观察孩子的症状，防止复发。

②安排复查：按医嘱进行复查，调整后续治疗，预防再次发作。

**问题95红区哮喘患儿使用全身性糖皮质激素有哪些注意事项？**

（1）用药时机与剂量

①及时启动：当患儿的喘息症状严重，支气管扩张剂（如沙丁胺醇）效果不佳时，医生可能会建议使用全身性糖皮质激素。

②合理控制剂量：剂量需要根据孩子的体重和症状调整。使用最低有效剂量，以减少副作用风险。

（2）疗程控制

①短期使用：通常只用3-5天，如果症状缓解，可能不再需要继续使用；如症状持续，应按医生建议调整用药。

②逐渐停药：如果用药超过一周，应在医生指导下逐步减少剂量，而不是突然停药，以避免激素反应。

（3）副作用监测

①短期副作用：孩子可能会出现胃肠不适、情绪波动、睡眠障碍或血糖变化。家属应留意孩子的表现，并减少高糖食物摄入。

②长期副作用：虽然一般是短期用药，但频繁使用激素可能影响生长和免疫力。每次使用后，建议记录孩子的体重、身高等，方便医生随访。

（4）药物相互作用

避免其他药物冲突：全身性糖皮质激素可能与其他药物（如非甾体抗炎药）发生相互作用。用药期间请避免自行服用其他药，尤其是非处方药。

（5）随访与家属教育

①按时复诊：用药后，家长应带孩子定期复诊，记录症状变化和用药效果，帮助医生判断治疗效果。

②理解药物作用：家属应了解激素的作用和常见副作用，遵从医生建议按疗程用药，并了解停药方法，避免误用。

**问题96对于反复处于红区的哮喘患儿，如何进行治疗方案的调整及长期管理？**

1.重新评估现有治疗

（1）检查当前用药：了解是否基础药物不足或使用不规律，药物剂量可能需要加强。

（2）识别诱因：避免接触已知的过敏原或诱因，如尘螨、烟雾、寒冷空气等。

检查吸入技术：确保吸入药物的正确使用，药物才能有效作用。

2. 加强日常用药管理

（1）增加激素药物剂量：若低剂量无效，可调整为中或高剂量吸入性糖皮质激素（ICS），或在其基础上加入长效β2激动剂（LABA）。

（2）急性期短期用药：若哮喘急性发作，短期内加大ICS剂量或口服糖皮质激素。短期治疗通常不超过5-7天。

3. 控制和预防诱发因素

（1）减少过敏原暴露：如保持家居清洁、使用防螨床上用品、定期通风。

（2）避免烟雾和污染：保持无烟环境，并减少接触空气污染。

（3）预防感染：如接种流感疫苗，防止呼吸道感染诱发哮喘。

4. 监测病情变化

（1）使用峰流速计：在家中监测患儿的呼气流速（PEF），每日记录可帮助识别病情变化。

（2）定期随访：每3-6个月随访一次病情。若控制不佳，可提高随访频率，直到病情稳定。

5. 制定哮喘管理计划

（1）红区、黄区、绿区行动指南：帮助家长了解不同情况的处理方法。

（2）明确药物用法：包括维持药物和急救药物的剂量和使用频率。

（3）紧急情况处理：如急性发作时用沙丁胺醇，并明确何时需要就医。

6. 关注心理健康和家长教育

（1）为家长提供教育：帮助家长了解哮喘的基本知识、药物的作用和使用方法，增强他们的信心。

（2）心理支持：帮助患儿减轻对哮喘的恐惧，避免焦虑影响病情。

7. 监控生长发育：长期使用糖皮质激素可能影响儿童的身高和体重，因此每次随访应监测患儿的成长情况，必要时由医生调整治疗，平衡哮喘控制和健康发育。

8. 转诊至专科：如果加强管理后患儿仍频繁进入红区，建议转诊至专科医生，进一步检查是否有其他潜在问题，并通过专业检测优化治疗。

**问题97如何在医院开展儿童哮喘的健康教育、推行CCAAP？**

1.建立有效的健康教育计划

（1）组建团队：医生、护士、呼吸治疗师等组成团队，共同负责教育计划的设计和执行，确保内容专业、全面。

（2）按阶段分步骤教育：根据儿童年龄和病情，把教育内容分为基础知识、病情管理、药物使用等不同阶段，逐步引导家长和孩子掌握哮喘管理技巧。

（3）个性化教育：每个孩子的哮喘情况不同。需要根据个体的药物敏感性、过敏原、生活环境等调整教育内容，使建议更具针对性。

2.健康教育的主要内容

（1）哮喘知识普及：

①解释哮喘的基本原理，如气道炎症、呼吸道反应过度等。

②帮助家长和孩子识别哮喘症状，如咳嗽、喘息、气短等。

（2）触发因素管理：

①常见触发因素包括过敏原（尘螨、花粉等）、气温变化和情绪波动。

②教家长如何减少孩子接触这些触发因素，如保持室内清洁、避免吸烟环境等。

（3）药物管理与使用技巧：

①介绍哮喘的两种药物：控制药物（如吸入类固醇）和缓解药物（如短效β₂受体激动剂），说明它们的作用。

②教授正确使用吸入装置，如雾化器和吸入器，确保药物效果。

（4）自我监测和管理：

①家长和孩子可用峰流速仪来监测哮喘控制情况，并根据结果调整管理措施。

②教家长了解哮喘的绿、黄、红灯区判断方法，以便及时识别和应对症状变化。

（5）急性发作处理方案：

为每个孩子制定应急处理计划，包括使用缓解药的具体方法和必要时的就医指引，确保家长在发作时知道该怎么办。

3.实施CCAAP的措施

（1）提供哮喘行动计划：为家长提供纸质和电子版的CCAAP，详细列出管理和应急措施，供日常参考。

（2）定期随访：建立哮喘管理档案，定期随访评估孩子的哮喘控制情况，在随访中调整教育内容和管理计划。

（3）推广教育资源：

①在医院设立哮喘教育区，提供相关资料和视频课程。

②提供在线平台或APP，方便家长随时获取哮喘知识和专家建议。

（4）家庭和学校支持：为家长、学校教师和校医提供哮喘管理知识和应急处理方法，确保孩子在校内也能得到有效支持。

4.效果评估与反馈

①效果评估：定期检查哮喘教育和CCAAP实施后的效果，比如哮喘控制情况和急诊次数，判断教育是否有效。

②反馈与调整：收集家长和孩子的意见，分析实际问题，适时改进教育内容和管理方案，使其更具实用性。

5.常见问题的纠正

纠正家长和孩子常见的误区，例如依赖缓解药物或错误的吸入方法。通过重复讲解，纠正错误观念，增强他们的管理信心。

**问题98如何在社区开展儿童哮喘的健康教育及防治工作？**

1. 提高家长和孩子对哮喘的认识

①普及哮喘知识：通过社区讲座、宣传手册和海报，介绍哮喘的原因、症状和诱发因素，帮助家长了解哮喘的慢性特点和管理重要性。

②教会识别哮喘症状：教家长识别哮喘的常见症状，如喘息、咳嗽和胸闷，尤其是夜间症状。帮助家长学会处理哮喘发作，如保持冷静、按医嘱使用缓解药物等。

2. 教育哮喘管理方法

①制定个性化管理计划：为每个患儿制定哮喘管理计划，包括日常管理、急性发作时的处理方案、用药计划等。

②指导正确用药：向家长和孩子解释控制药物（如吸入性糖皮质激素）和缓解药物的作用，并教正确的吸入器使用方法。

3. 环境控制和过敏原管理

①保持室内清洁：建议家庭定期清洁，以减少尘螨、霉菌和宠物毛等常见过敏原，避免二手烟和刺激性气味。

②减少户外刺激：天气不好或空气污染时，减少户外活动，出门戴口罩。避免儿童剧烈运动或暴露在冷空气中。

4. 建立定期随访和应急预案

①定期复诊：鼓励家长带孩子到社区卫生中心定期复查，监测症状、检查肺功能和评估用药情况。

②应急联系网络：社区内建立应急网络，方便医生、家长、学校和社区工作人员在紧急情况时及时协助。

5. 与学校合作

①在学校支持管理：与学校沟通，为教师和校医提供哮喘管理培训，确保孩子在校期间的安全，尤其是体育课期间。

②定期健康教育：定期组织患儿及家长的哮喘知识课堂，教孩子认识和管理哮喘，减少对哮喘的心理压力。

6. 使用健康管理工具

①智能工具记录症状：利用手机应用记录孩子的哮喘症状、用药情况和肺功能数据，方便家长和医务人员了解病情变化。

②远程指导：社区卫生中心可以通过电话或网络，为家长提供哮喘管理的远程指导，解答疑问，提醒定期复诊。

7. 建立家长互助小组

经验交流与支持：在社区内组织哮喘患儿家长的支持小组，分享管理经验，增强家长的信心，减轻孤独感。

**问题99如何在校园做好儿童哮喘的健康教育及防治工作？**

1. 加强哮喘知识教育

（1）定期讲座：学校可为学生、家长和教师举办哮喘讲座，介绍哮喘是什么、有哪些症状、怎么应对。帮助大家了解哮喘发作的表现和处理办法。

（2）分发宣传材料：提供简明的哮喘小册子和卡片，介绍诱因、症状和急救措施，确保学生和家长能在发作时正确应对。

（3）利用校园媒介：通过校园广播、公告栏或微信公众号传播哮喘知识，让大家随时了解哮喘的基本常识。

2. 建立个人哮喘管理计划

（1）个性化健康档案：校医室为每位哮喘患儿建立档案，记录发病史、触发因素和常用药物等信息，方便急救时参考。

（2）日常用药提醒：按患儿医生的建议，帮助学生合理安排用药，提醒他们在发作风险高时按需用药。

（3）吸入器使用培训：教患儿正确使用吸入器，确保他们在没有家长陪伴时也能安全用药。

3. 营造无刺激的校园环境

（1）减少诱发因素：避免在教室中使用刺激性化学品，少用空气清新剂，避免种植易引发过敏的植物，减少哮喘诱因。

（2）监测空气质量：在空气差的日子里减少户外活动。学校可考虑安装空气质量监测设备，帮助管理活动安排。

（3）加强通风清洁：定期清洁校园环境，保持良好通风，减少尘螨和霉菌积累，降低过敏源。

4. 建立应急处理流程

（1）制定应急预案：明确哮喘发作的处理步骤，包括识别症状、施用急救药物、紧急呼救等，确保教师和校医会处理急发情况。

（2）备足急救药物：校医室应常备应急药物如短效吸入剂，必要时供患儿使用。教师也应了解基本用药流程。

（3）急救技能培训：为教职工和高年级学生提供急救培训，帮助大家掌握基础急救技能，以便在医护人员到来前提供帮助。

5. 加强家校医院协作

（1）及时沟通：学校应与家长定期联系，了解患儿的健康状况，发现问题及时沟通，避免忽视病情。

（2）三方协作管理：学校、家庭和医院共同制定哮喘管理方案，定期检查和调整措施，确保在校内外一体化管理。

（3）提高患儿自我管理意识：帮助年长患儿学习哮喘管理技能，包括避开诱因和急救方法，逐步提升他们的自我管理能力。

6. 鼓励适当的体育活动和健康生活习惯

（1）合理安排运动：学校在空气良好的日子里安排适当运动，并提醒患儿按医嘱在运动前使用预防药物，避免过度剧烈运动。

（2）健康生活教育：倡导良好的饮食、睡眠和运动习惯，以增强体质，提高免疫力，有助于减少哮喘发作的风险。

**问题100如何利用互联网平台做好儿童哮喘的健康教育工作？**

1. 在线健康教育课程

（1）平台选择：家长可以通过微信公众号、健康管理App、视频网站等学习儿童哮喘的相关知识。课程内容包括哮喘症状、药物使用、避免过敏原和急救措施等。

（2）互动功能：这些平台常有问答和互动环节，家长可以随时向医生提问，解决疑虑。

2. 定期发布科普文章和短视频

（1）文章和视频：平台可以定期发布简短的文章和视频，讲解儿童哮喘的基础知识、药物使用方法等。这些内容容易理解，适合家长在空闲时查看。

（2）多媒体内容：通过图文、动画、视频等形式展示知识，使内容更生动易懂。

3. 在线健康监测与数据共享

（1）健康管理App：一些专门的App可以帮助家长记录孩子的症状、药物使用情况等。平台会根据这些数据提醒家长按时给孩子用药或检查健康状态。

（2）医生远程指导：家长通过平台与医生沟通，得到个性化的健康管理建议。

4. 线上支持小组与社交平台

（1）家长交流群：建立线上小组，家长可以分享经验和互相支持，解决育儿中的困惑。

（2）专家答疑：通过平台定期举办在线讲座，邀请专家为家长解答问题，提高哮喘管理水平。

5. 远程跟踪和智能干预

（1）远程监测：通过智能设备，医生可以远程监控孩子的病情变化，及时调整治疗方案。

（2）智能提醒：平台可设置提醒，帮助家长按时给孩子用药、复诊或避免过敏原。

6. 个性化健康教育内容

（1）定制内容：平台根据孩子的健康情况，推送个性化的教育内容。例如，针对过敏性哮喘的孩子，平台会提供过敏原管理的具体建议。

（2）行为指导：平台根据数据分析，帮助家长改善孩子的生活习惯，如饮食、睡眠等。

7. 公益活动和线上宣传

（1）公益项目：通过互联网平台推动一些公益活动，例如“哮喘知识日”，邀请专家通过直播向家长普及知识。

（2）线上宣传：利用社交媒体发布儿童哮喘相关的科普内容，提高公众对疾病的认知。
