# Supplementary material for: Rethinking Pediatric Asthma Education Through Large Language Model Generation and Simplification: Randomized Double-Blind Study
Source: J Med Internet Res. 2026 Aug 26;28:e98118. doi: 10.2196/98118 (PMC13514414; doi:10.2196/98118)
Supplement: Supplementary file 2 [file jmir-v28-e98118-s002.docx]

**Pre-Evaluation Screening of the AI Response**

**Table S1. Appropriateness Assessment Questionnaire**

**Items**

1. How would you rate the accuracy of the response to this question?

① Very inaccurate ② Inaccurate ③ Neutral ④ Generally accurate ⑤ Very accurate

2. How would you rate the comprehensiveness of the response to this question?

① Very incomplete ② Incomplete ③ Neutral ④ Generally comprehensive ⑤ Very comprehensive

3. How would you rate the clarity of the response to this question?

① Very unclear ② Unclear ③ Neutral ④ Generally clear ⑤ Very clear

**Note: A 5-point Likert scale was used. A response was considered inappropriate if it received a score below 2 on any of the three assessment criteria.*

**Table S2. Pre-Evaluation Screening Results**

| **Question No.** | **AI-GRs** | | | **AI-SRs** | | |
| --- | --- | --- | --- | --- | --- | --- |
|  | accuracy | comprehensiveness | clarity | accuracy | comprehensiveness | clarity |
| **1** | 5 | 5 | 5 | 2 | 2 | 3 |
| **2** | 3 | 4 | 5 | 3 | 4 | 5 |
| **3** | 4 | 4 | 5 | 4 | 4 | 3 |
| **4** | 5 | 5 | 5 | 4 | 4 | 4 |
| **5** | 2 | 2 | 2 | 2 | 1 | 2 |
| **6** | 4 | 4 | 5 | 2 | 2 | 2 |
| **7** | 5 | 5 | 5 | 4 | 4 | 4 |
| **8** | 3 | 3 | 4 | 3 | 2 | 3 |
| **9** | 4 | 4 | 4 | 4 | 4 | 4 |
| **10** | 5 | 5 | 5 | 4 | 4 | 4 |
| **11** | 1 | 1 | 3 | 1 | 2 | 3 |
| **12** | 4 | 4 | 4 | 4 | 4 | 3 |
| **13** | 5 | 5 | 5 | 4 | 4 | 4 |
| **14** | 5 | 5 | 5 | 5 | 5 | 5 |
| **15** | 5 | 5 | 5 | 4 | 3 | 3 |
| **16** | 5 | 5 | 5 | 5 | 5 | 5 |
| **17** | 5 | 5 | 5 | 4 | 4 | 4 |
| **18** | 5 | 5 | 5 | 5 | 5 | 5 |
| **19** | 5 | 5 | 5 | 2 | 2 | 4 |
| **20** | 4 | 4 | 5 | 4 | 2 | 4 |
| **21** | 4 | 2 | 4 | 4 | 1 | 4 |
| **22** | 5 | 5 | 5 | 5 | 5 | 5 |
| **23** | 5 | 5 | 5 | 4 | 2 | 4 |
| **24** | 4 | 2 | 4 | 3 | 1 | 4 |
| **25** | 5 | 5 | 4 | 4 | 4 | 4 |
| **26** | 1 | 2 | 2 | 1 | 1 | 2 |
| **27** | 5 | 5 | 5 | 5 | 5 | 5 |
| **28** | 5 | 5 | 5 | 4 | 4 | 4 |
| **29** | 2 | 1 | 2 | 2 | 1 | 1 |
| **30** | 3 | 3 | 4 | 5 | 5 | 5 |
| **31** | 2 | 2 | 4 | 1 | 1 | 3 |
| **32** | 3 | 3 | 5 | 2 | 3 | 3 |
| **33** | 5 | 5 | 5 | 4 | 3 | 3 |
| **34** | 5 | 5 | 5 | 2 | 3 | 3 |
| **35** | 4 | 5 | 5 | 3 | 4 | 4 |
| **36** | 4 | 4 | 5 | 4 | 4 | 4 |
| **37** | 1 | 2 | 4 | 1 | 2 | 2 |
| **38** | 1 | 1 | 2 | 1 | 1 | 2 |
| **39** | 1 | 1 | 3 | 1 | 1 | 2 |
| **40** | 5 | 5 | 5 | 4 | 5 | 4 |
| **41** | 5 | 5 | 5 | 4 | 4 | 4 |
| **42** | 5 | 5 | 5 | 4 | 4 | 4 |
| **43** | 5 | 5 | 5 | 5 | 5 | 5 |
| **44** | 5 | 5 | 5 | 5 | 5 | 5 |
| **45** | 5 | 5 | 5 | 5 | 5 | 5 |
| **46** | 5 | 5 | 5 | 4 | 4 | 5 |
| **47** | 5 | 5 | 5 | 5 | 5 | 5 |
| **48** | 5 | 5 | 5 | 5 | 5 | 5 |
| **49** | 5 | 5 | 5 | 5 | 5 | 5 |
| **50** | 2 | 4 | 4 | 1 | 4 | 4 |
| **51** | 5 | 5 | 5 | 5 | 5 | 5 |
| **52** | 5 | 5 | 5 | 5 | 5 | 5 |
| **53** | 5 | 5 | 5 | 5 | 5 | 5 |
| **54** | 4 | 4 | 5 | 5 | 5 | 5 |
| **55** | 5 | 5 | 5 | 5 | 5 | 5 |
| **56** | 5 | 5 | 5 | 5 | 5 | 5 |
| **57** | 5 | 5 | 5 | 5 | 5 | 5 |
| **58** | 1 | 1 | 2 | 1 | 1 | 2 |
| **59** | 5 | 5 | 5 | 5 | 5 | 5 |
| **60** | 5 | 5 | 5 | 5 | 5 | 5 |
| **61** | 5 | 5 | 5 | 4 | 4 | 5 |
| **62** | 3 | 4 | 4 | 2 | 3 | 2 |
| **63** | 5 | 5 | 5 | 5 | 5 | 5 |
| **64** | 5 | 5 | 5 | 5 | 5 | 5 |
| **65** | 5 | 5 | 5 | 5 | 5 | 5 |
| **66** | 5 | 5 | 5 | 5 | 5 | 5 |
| **67** | 5 | 5 | 5 | 5 | 5 | 5 |
| **68** | 5 | 5 | 5 | 5 | 5 | 5 |
| **69** | 5 | 5 | 5 | 5 | 5 | 5 |
| **70** | 5 | 5 | 5 | 5 | 5 | 5 |
| **71** | 5 | 5 | 5 | 5 | 5 | 5 |
| **72** | 5 | 5 | 5 | 5 | 5 | 5 |
| **73** | 5 | 5 | 5 | 5 | 5 | 5 |
| **74** | 5 | 5 | 5 | 5 | 5 | 5 |
| **75** | 5 | 5 | 5 | 4 | 5 | 5 |
| **76** | 5 | 5 | 5 | 5 | 5 | 5 |
| **77** | 5 | 5 | 5 | 2 | 2 | 4 |
| **78** | 5 | 5 | 5 | 5 | 5 | 5 |
| **79** | 5 | 5 | 5 | 5 | 5 | 5 |
| **80** | 5 | 5 | 5 | 5 | 5 | 5 |
| **81** | 4 | 3 | 4 | 5 | 5 | 5 |
| **82** | 5 | 5 | 5 | 5 | 5 | 5 |
| **83** | 5 | 5 | 5 | 5 | 5 | 5 |
| **84** | 5 | 5 | 5 | 4 | 5 | 5 |
| **85** | 5 | 5 | 5 | 5 | 5 | 5 |
| **86** | 5 | 5 | 5 | 5 | 5 | 5 |
| **87** | 5 | 5 | 5 | 5 | 4 | 5 |
| **88** | 4 | 4 | 5 | 5 | 5 | 5 |
| **89** | 5 | 5 | 5 | 4 | 4 | 5 |
| **90** | 5 | 5 | 5 | 5 | 5 | 5 |
| **91** | 5 | 5 | 5 | 5 | 5 | 5 |
| **92** | 2 | 2 | 3 | 1 | 2 | 2 |
| **93** | 5 | 5 | 5 | 5 | 5 | 5 |
| **94** | 5 | 5 | 5 | 5 | 5 | 5 |
| **95** | 5 | 5 | 5 | 5 | 5 | 5 |
| **96** | 5 | 5 | 5 | 5 | 5 | 5 |
| **97** | 5 | 5 | 5 | 5 | 5 | 5 |
| **98** | 5 | 5 | 5 | 5 | 5 | 5 |
| **99** | 5 | 5 | 5 | 5 | 5 | 5 |
| **100** | 5 | 5 | 5 | 5 | 5 | 5 |

**Note: Questions highlighted in grey were identified as having inappropriate responses. In the actual study, each such question and its corresponding responses were simultaneously excluded from all three response banks (PEARs, AI-GRs, and AI-SRs).*
